# High-depth African genomes inform human migration and health

**DOI:** 10.1038/s41586-020-2859-7

**Published:** 2020-10-28

**Authors:** Ananyo Choudhury, Shaun Aron, Laura R. Botigué, Dhriti Sengupta, Gerrit Botha, Taoufik Bensellak, Gordon Wells, Judit Kumuthini, Daniel Shriner, Yasmina J. Fakim, Anisah W. Ghoorah, Eileen Dareng, Trust Odia, Oluwadamilare Falola, Ezekiel Adebiyi, Scott Hazelhurst, Gaston Mazandu, Oscar A. Nyangiri, Mamana Mbiyavanga, Alia Benkahla, Samar K. Kassim, Nicola Mulder, Sally N. Adebamowo, Emile R. Chimusa, Donna Muzny, Ginger Metcalf, Richard A. Gibbs, TrypanoGEN Research Group, Enock Matovu, Bruno Bucheton, Christiane Hertz-Fowler, Mathurin Koffi, Annette Macleod, Dieudonne Mumba-Ngoyi, Harry Noyes, Oscar A. Nyangiri, Gustave Simo, Martin Simuunza, Charles Rotimi, Michèle Ramsay, H3Africa Consortium, Ananyo Choudhury, Shaun Aron, Laura Botigué, Dhriti Sengupta, Gerrit Botha, Taoufik Bensellak, Gordon Wells, Judit Kumuthini, Daniel Shriner, Yasmina J. Fakim, Anisah W. Ghoorah, Eileen Dareng, Trust Odia, Oluwadamilare Falola, Ezekiel Adebiyi, Scott Hazelhurst, Gaston Mazandu, Oscar A. Nyangiri, Mamana Mbiyavanga, Alia Benkahla, Samar K. Kassim, Nicola Mulder, Sally N. Adebamowo, Emile R. Chimusa, Charles Rotimi, Michèle Ramsay, Adebowale A. Adeyemo, Zané Lombard, Neil A. Hanchard, Clement Adebamowo, Godfred Agongo, Romuald P. Boua, Abraham Oduro, Hermann Sorgho, Guida Landouré, Lassana Cissé, Salimata Diarra, Oumar Samassékou, Gabriel Anabwani, Mogomotsi Matshaba, Moses Joloba, Adeodata Kekitiinwa, Graeme Mardon, Sununguko W. Mpoloka, Samuel Kyobe, Busisiwe Mlotshwa, Savannah Mwesigwa, Gaone Retshabile, Lesedi Williams, Ambroise Wonkam, Ahmed Moussa, Dwomoa Adu, Akinlolu Ojo, David Burke, Babatunde O. Salako, Enock Matovu, Bruno Bucheton, Christiane Hertz-Fowler, Mathurin Koffi, Annette Macleod, Dieudonne Mumba-Ngoyi, Harry Noyes, Oscar A. Nyangiri, Gustave Simo, Martin Simuunza, Philip Awadalla, Vanessa Bruat, Elias Gbeha, Adebowale A. Adeyemo, Zané Lombard, Neil A. Hanchard

**Affiliations:** 1 Sydney Brenner Institute for Molecular Bioscience, Faculty of Health Sciences, University of the Witwatersrand, Johannesburg, South Africa; 2 Center for Research in Agricultural Genomics (CRAG), Plant and Animal Genomics Program, CSIC-IRTA-UAB-UB, Barcelona, Spain; 3 Computational Biology Division and H3ABioNet, Department of Integrative Biomedical Sciences, IDM, University of Cape Town, Cape Town, South Africa; 4 System and Data Engineering Team, Abdelmalek Essaadi University, ENSA, Tangier, Morocco; 5 Centre for Proteomic and Genomic Research (CPGR), Cape Town, South Africa; 6 South African National Bioinformatics Network, University of the Western Cape, Bellville, South Africa; 7 Center for Research on Genomics and Global Health, National Human Genome Research Institute, National Institutes of Health, Bethesda, MD USA; 8 Department of Agriculture and Food Science, Faculty of Agriculture, University of Mauritius, Reduit, Mauritius; 9 Department of Digital Technologies,Faculty of Information, Communication & Digital Technologies, University of Mauritius, Reduit, Mauritius; 10 Department of Public Health and Primary Care, University of Cambridge, Cambridge, UK; 11 Institute of Human Virology Nigeria, Abuja, Nigeria; 12 Covenant University Bioinformatics Research (CUBRe), Covenant University, Ota, Nigeria; 13 Department of Computer and Information Sciences, Covenant University, Ota, Nigeria; 14 School of Electrical and Information Engineering, University of the Witwatersrand, Johannesburg, South Africa; 15 College of Veterinary Medicine, Animal Resources and Biosecurity, Makerere University, Kampala, Uganda; 16 Laboratory of Bioinformatics, Biomathematics and Biostatistics (BIMS), Institute Pasteur of Tunis, Tunis, Tunisia; 17 Medical Biochemistry and Molecular Biology Department, Faculty of Medicine, Ain Shams University, Abbaseya, Cairo, Egypt; 18 Department of Epidemiology and Public Health, University of Maryland School of Medicine, University of Maryland Baltimore, Baltimore, MD USA; 19 University of Maryland Greenebaum Comprehensive Cancer Center, University of Maryland School of Medicine, University of Maryland Baltimore, Baltimore, MD USA; 20 Division of Human Genetics, Department of Pathology, Faculty of Health Sciences, Institute for Infectious, Disease and Molecular Medicine, University of Cape Town, Cape Town, South Africa; 21 Human Genome Sequencing Center, Baylor College of Medicine, Houston, TX USA; 22 Department of Molecular and Human Genetics, Baylor College of Medicine, Houston, Texas USA; 23 Division of Human Genetics, National Health Laboratory Service, and School of Pathology, Faculty of Health Sciences, University of the Witwatersrand, Johannesburg, South Africa; 24 Institute of Human Virology and Greenebaum Cancer Center, University of Maryland School of Medicine, Baltimore, MD USA; 25 Navrongo Health Research Centre, Navrongo, Ghana; 26 Clinical Research Unit of Nanoro, Institut de Recherche en Sciences de la Sante, Bobo-Dioulasso, Burkina Faso; 27 Faculty of Medicine and Odontostomatology, University of Science, Techniques and Technologies of Bamako (USTTB), Bamako, Mali; 28 Service de Neurologie, Centre Hospitalier Universitaire du Point “G”, Bamako, Mali; 29 Neurogenetics Branch, National Institute of Neurological Disorders and Stroke, National Institutes of Health, Bethesda, MD USA; 30 Botswana-Baylor Children’s Clinical Centre of Excellence, Gaborone, Botswana; 31 Medical Microbiology, College of Health Sciences, Makerere University, Kampala, Uganda; 32 Baylor College of Medicine Children’s Foundation, Kampala, Uganda; 33 Department of Pathology and Immunology, Baylor College of Medicine, Houston, TX USA; 34 Department of Biological Sciences, University of Botswana, Gaborone, Botswana; 35 University of Ghana Medical School, Accra, Ghana; 36 The University of Kansas School of Medicine, Kansas City, KS USA; 37 Department of Human Genetics, University of Michigan Medical School, Ann Arbor, MI USA; 38 College of Medicine, University of Ibadan, Ibadan, Nigeria; 39 Institut de Recherche pour le Développement, Montpellier, France; 40 Institute of Integrative Biology, University of Liverpool, Liverpool, UK; 41 Wellcome Trust, London, UK; 42 Jean Lorougnon Guede University, Daloa, Côte d’Ivoire; 43 Institute of Biodiversity Animal Health and Comparative Medicine, University of Glasgow, Glasgow, UK; 44 Institut National de Recherche Biomedicale, Kinshasa, Democratic Republic of Congo; 45 Faculty of Science, University of Dschang, Dschang, Cameroon; 46 School of Veterinary Medicine, University of Zambia, Lusaka, Zambia; 47 Department of Molecular Genetics, University of Toronto, Toronto, Ontario Canada; 48 Ontario Institute for Cancer Research, Toronto, Ontario Canada

**Keywords:** Evolutionary genetics, Genetic variation, Next-generation sequencing, Genetics research

## Abstract

The African continent is regarded as the cradle of modern humans and African genomes contain more genetic variation than those from any other continent, yet only a fraction of the genetic diversity among African individuals has been surveyed^1^. Here we performed whole-genome sequencing analyses of 426 individuals—comprising 50 ethnolinguistic groups, including previously unsampled populations—to explore the breadth of genomic diversity across Africa. We uncovered more than 3 million previously undescribed variants, most of which were found among individuals from newly sampled ethnolinguistic groups, as well as 62 previously unreported loci that are under strong selection, which were predominantly found in genes that are involved in viral immunity, DNA repair and metabolism. We observed complex patterns of ancestral admixture and putative-damaging and novel variation, both within and between populations, alongside evidence that Zambia was a likely intermediate site along the routes of expansion of Bantu-speaking populations. Pathogenic variants in genes that are currently characterized as medically relevant were uncommon—but in other genes, variants denoted as ‘likely pathogenic’ in the ClinVar database were commonly observed. Collectively, these findings refine our current understanding of continental migration, identify gene flow and the response to human disease as strong drivers of genome-level population variation, and underscore the scientific imperative for a broader characterization of the genomic diversity of African individuals to understand human ancestry and improve health.

## Abstract (web summary)

Whole-genome sequencing analyses of African populations provide insights into continental migration, gene flow and the response to human disease, highlighting the importance of including diverse populations in genomic analyses to understand human ancestry and improve health.

## Main

Advances in genomics have empowered the interrogation of the human genome across global populations^2^, with the resulting studies demonstrating that Africa harbours the most genetic variation and diversity^3,4^. These studies provided insights into medically relevant genetic loci and aided in the interpretation of the pathogenicity of genetic variants^5^, advancing precision medicine for all populations^6^. To date, only a limited number of the around 2,000 African ethnolinguistic groups have been genetically characterized, predominantly using genotyping arrays, which contained a limited number of variants that are common in European populations. The population distribution of novel, rare and medically relevant variation among African individuals thus remains largely unknown, which adversely affects our understanding of the genetic contributions to Mendelian and complex diseases^7,8^.

Classically, sub-Saharan African populations have been described in the context of four major language families: Afro-Asiatic (AA), Nilo-Saharan (NS), Niger–Congo (NC)—which includes the Bantu language family—and the Khoe and San (KS) languages^3^. This broad classification remains as a framework, although several language families contain independent groups; the Khoe and San families, for example, are contestedly grouped together as KhoeSan in the literature despite having distinct histories (Supplementary Note 1.1). The Bantu languages are the most widely spoken in sub-Saharan Africa, and this broad dispersion has been ascribed to a series of migrations across the continent over the past 5,000 years^9^. These migration events and subsequent admixture with resident populations have had a pivotal role in shaping the genomic landscape of Africa, as it involved adaptations to new exposures and experiences. The signatures of these adaptations are evident in patterns of allelic variation associated with key physiological traits or prevalent communicable diseases, exemplified by variations in *HBB*^10^, *LCT*^11^, *APOL1*^12^ and *G6PD*^13^. Recent studies have identified signatures of selection that reflect the importance of new variation, introduced by admixture, to traits as diverse as diet, height, blood pressure and skin pigmentation^4,9,14–16^.

**Fig. 1:**
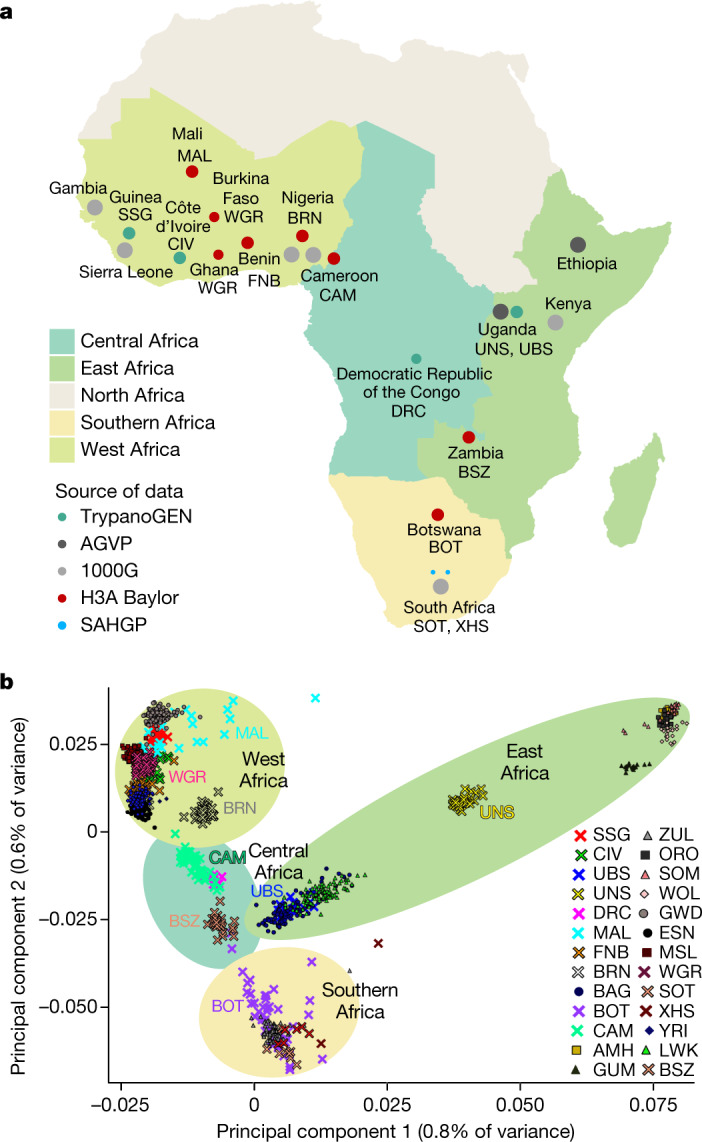
H3Africa WGS data. **a**, Geographical regions and populations of origin for H3Africa WGS data. The size of the circles indicates the relative number of sequenced samples from each population group (before quality control; Supplementary Methods Table 1). Samples with WGS data from the 1000 Genomes Project and the African Genome Variation Project are included for comparison (grey circles). CAM includes 25 individuals who are homozygous for the sickle mutation (HbSS); MAL includes unaffected individuals with a family history of neurological disease; BOT comprises children who are HIV-positive; BRN included only female participants. 1000G, 1000 Genome Project; AGVP, African Genome Variation Project. Maps were created using R^43^. **b**, Principal component analysis of African WGS data showing the first two principal components. New populations used in this study are indicated by crosses. Population abbreviations are as described in the 1000 Genomes and H3Africa Projects as provided in Supplementary Methods Table 1 and Supplementary Table 22. Shaded background elipses relate to the geographical regions as shown in **a**.

Studying the genomes of peoples across the breadth of Africa presents unique opportunities for understanding the population demography of human disease. The Human Heredity and Health in Africa (H3Africa) Consortium was conceived to redress the dearth of genomics research in Africa^8^, and, to date, supports 48 projects across 34 countries. An important mandate of H3Africa is to characterize genetic diversity across Africa to facilitate a framewok for genomic research. To this end, we analysed whole-genome sequencing (WGS) data generated in 426 individuals from ongoing H3Africa studies, including 314 high-depth (average depth of coverage, 30×) and 112 medium-depth (average depth of coverage, 10×) whole-genome sequences, encompassing 50 ethnolinguistic groups from 13 countries across Africa (Fig. 1a and Supplementary Methods Table 1). Some of these groups are studied here for the first time, providing a unique overview of the diverse landscape of African genomic variation.

Our analyses focused on single-nucleotide variants (SNVs) in samples from three African resources: the H3Africa Consortium (here H3A-Baylor; http://www.h3africa.org/)^8^, the Southern African Human Genome Programme (SAHGP)^17^ and the Trypanosomiasis Genomics Network of the H3Africa Consortium (TryopanoGEN)^18^ (Methods). High depth of coverage WGS data from H3A-Baylor encompassing 314 individuals from west (Burkina Faso, Ghana, Mali, Nigeria, Benin and Cameroon), central (Zambia) and south (Botswana) African countries (Supplementary Methods Table 1) were used for analyses of rare and novel variation, and to identify selective sweeps. The full dataset was used for analyses of population ancestry and admixture, and to assess medically relevant variation. A total of 41,645,936 high-quality SNVs were identified across all groups; of these, 31,160,639 were found in the dataset that had a high depth of coverage (HC-WGS dataset).

## Insights into migration and admixture

A major focus of our survey was to bridge the gaps in African population-scale WGS data by including samples from understudied geographical regions. To examine this, we first contextualized our understudied populations alongside previous African WGS efforts^2,4,17^ using principal component analysis (Fig. 1b and Supplementary Fig. 1). As previously observed^4^, the first principal component separated the NS, AA, and to some extent, east African NC speakers (Bantu-speaking individuals from Uganda (UBS)) from other NC speakers. The second principal component placed the remaining NC speakers along a cline from west to south (Fig. 1b). Individuals from Mali (MAL), which include some non-NC-speaking groups, were the notable exception, showing more dispersion between individuals. West African populations—the Fon from Benin (FNB), Gur speakers from west Africa (WGR), Soussou from Guinea (SSG), people living in Côte d’Ivoire (CIV) and MAL—projected in proximity to, but often independently of, other west African NC speakers such as the Yoruba (YRI) and Esan (ESN) from Nigeria^2^. Eastern (UBS) and southern African (Botswana (BOT)) NC speakers clustered with previously studied populations from their respective geographical regions.

Five population groups—the Berom of Nigeria (BRN), individals from CAM, individuals from the Democratic Republic of the Congo (DRC), Bantu speakers from Zambia (BSZ) and NS speakers from Uganda (UNS)—showed distinctive principal component localization. BRN samples localized independently of other west African populations, tending towards east African populations (Fig. 1b). Individuals from CAM and the DRC, consistent with the geographical proximity of these two countries, localized together, forming an independent central-west African group (Fig. 1b). UNS individuals localized independently of another NS population (Gumuz (GUZ)^4^) (Fig. 1b). Similarly, BSZ were found to be largely separate from other NC-speaking groups (Fig. 1b). Even in the context of a much wider representation of African populations from array-based data (Supplementary Fig. 2), some of these populations retained their distinctive localization patterns, demonstrating the extensive genomic diversity of the sampled populations. Accordingly, we observed a statistically significant positive correlation between geographical and genetic distances of NC-speaking populations (*R* = 0.96; *t*-statistic; *P* < 1 × 10^−9^) (Methods and Supplementary Fig. 3).

**Fig. 2:**
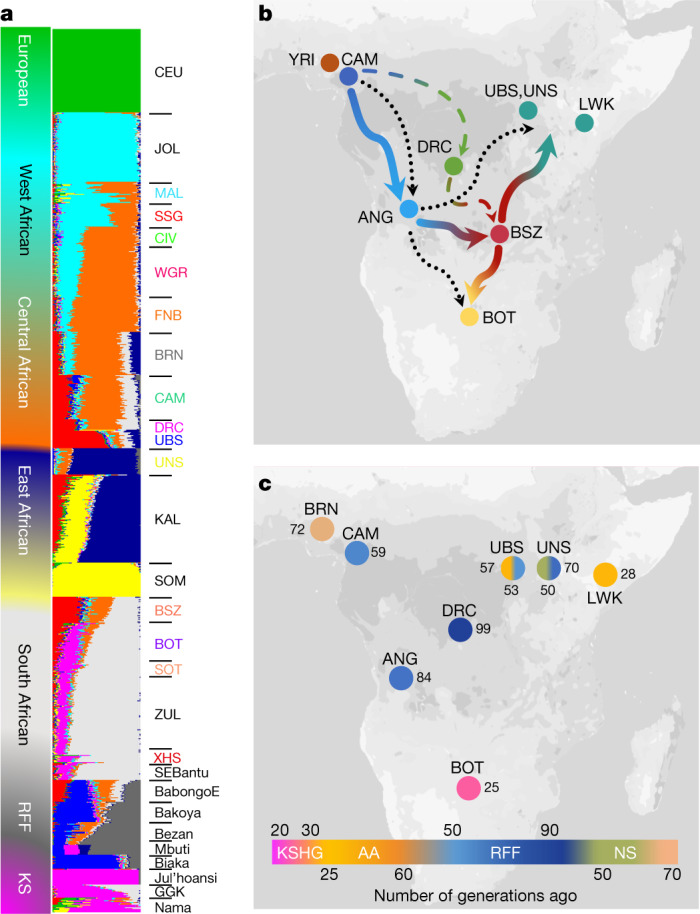
Population admixture and genetic ancestry among African populations. **a**, Admixture plot showing select African populations based on WGS and array data for *K* = 10. **b**, Proposed movement during the Bantu migration, showing the populations that were used for inference. Blue line shows the migration patterns inferred by genetic distance estimates with Zambia (BSZ) as an intermediate staging ground for Bantu migrations further east (red–teal arrow) and south (red–yellow arrow). The dotted black line shows the previously proposed late-split route^9^; the dotted blue–green line through the DRC indicates an alternative model of migration. GGK, Gǀwi, Gǁana and baKgalagadi. **c**, Key admixture dates (in generations) in populations of interest based on MALDER results. The colour of each circle represents the admixture date for NC components in each population group (KS, AA, RFF and NS). Dates are shown in terms of number of generations (1 generation = 29 years). Maps were created using R^43^.

**Extended Data Fig. 1:**
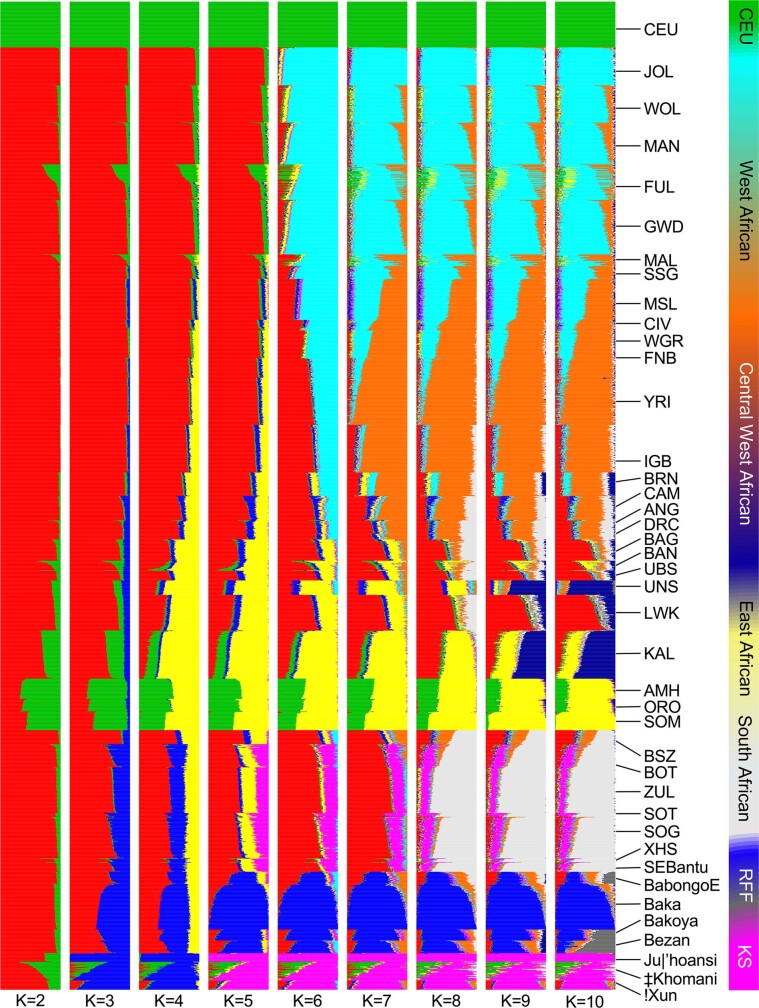
ADMIXTURE clustering analysis of H3A-WGS samples. Existing African datasets from AGVP^4^, 1000 Genomes project^2^, SAHGP^17^ and previously published studies^9,14^ and a representative European population (CEU) from the 1000 Genomes Project are included as reference panels. *K* values from 2 to 10 are shown. See Supplementary Table 22 for definitions of abbreviations.

We used an unsupervised clustering approach implemented in ADMIXTURE^19^ to evaluate whether gene flow from non-NC speakers differentiated our study populations (Fig. 2a, Extended Data Fig. 1 and Supplementary Fig. 4). Admixture events were then evaluated further using *f3*-statistics^20^ (Supplementary Table 1). Results were consistent with our current understanding of admixture patterns across the continent, showing KS gene flow in BOT; AA speaker gene flow in UBS, and varying degrees of gene flow from rainforest foragers (RFF; ethnolinguistically diverse peoples distributed across the forested regions of central Africa) in central-west African populations (DRC and CAM) (Fig. 2a and Extended Data Fig. 1). In addition, these analyses also revealed two interesting and unreported admixture events—RFF gene flow in UNS and gene flow from NS speakers in the BRN (Fig. 2a and Extended Data Fig. 1). Further analyses using additional population datasets (Supplementary Fig. 5 and Supplementary Note 1.2) suggested that the distinction between UNS and other NS-speaker populations could be due to increased gene flow from RFF as well as the absence of AA admixture. Traces of east African ancestry, originating from waves of trans-Sahelian migrations in the past few thousand years, have been reported in other populations, including the Hausa of Nigeria^3^; however, the observation of east African gene flow, possibly from Chad (Supplementary Fig. 6, Supplementary Table 2 and Supplementary Note 1.2), in the largest autochthonous central Nigerian population (BRN) is highly unique.

Analyses based on masking of non-NC ancestry in BOT highlighted the contribution of non-NC gene flow to the genetic distance between populations (Supplementary Note 1.3 and Supplementary Fig. 7). By contrast, BSZ, unlike their geographical neighbours from Angola, the DRC and Botswana, did not show evidence for any major gene flow from non-NC speakers, such as the RFF or KS groups (Fig. 2a and Extended Data Fig. 1). Similarly low levels of local group admixture have been noted for Bantu speakers from Malawi^16^ and Mozambique^21^. A recent attempt to reconstruct the route of Bantu migration across central Africa concluded that populations from Angola were the best source of Bantu speakers for east and south African Bantu speakers, and suggested a westerly route of Bantu-speaker migration via Angola^9^. Inclusion of the DRC and BSZ populations in our dataset, therefore, enabled us to further investigate this route (Fig. 2b). Principal component analysis and identity-by-descent sharing demonstrated that BSZ is genetically closer to both UBS and BOT compared to other central African populations (Supplementary Fig. 8). Moreover, formal admixture tests supported BSZ as the most likely central African source population for Bantu-speaker ancestries in east and south Africa (Supplementary Table 1). Furthermore, the degree of identity-by-descent sharing between population groups suggested that populations from Angola were the closest central or central-west African population to BSZ (Supplementary Fig. 8). Taken together, these estimates lead us to posit that Zambia was an intermediate site in the likely route of Bantu migration to both east and south Africa (Fig. 2b). An orthogonal approach using admixture graphing also supported this hypothesis (Supplementary Note 1.4).

Our attempts to estimate the dates of some key admixture events^22^ showed that KS gene flow in southern Africa and RFF gene flow in CAM were largely in agreement with previous studies^4,23^ (Fig. 2c and Supplementary Table 2). The date range for RFF admixture in UNS was similar to that for CAM and consistent with previous surveys^23^, hinting at a possibility for range alteration of RFF populations both east and west of the central rainforest around 60–70 generations ago (Fig. 2c). Previous studies on trans-Sahelian migration to west Africa have suggested two distinct waves of migration: one more than 100 generations ago (2,900 years ago) and a more-recent wave in the last 35 generations (1,015 years ago)^24^. On the basis of a variety of east African proxy populations, we estimated that admixture in BRN occurred approximately 50 to 70 generations ago (1,500–2,000 years ago) (Fig. 2c). These distinct dates are suggestive of a previously unknown demographic event, either at the local level, or possibly at a wider geographical scale (Supplementary Note 1.5).

Additional distinctive trends in the demographic history of some of these populations were observed, such as extensive variation in inter- and intra-ethnolinguistic groups within the defined geopolitical boundaries of Botswana, Cameroon and Mali (Supplementary Note 1.6, Supplementary Table 3 and Supplementary Fig. 9), and distinctively long segments of runs of homozygosity among MAL individuals (Supplementary Note 1.7 and Supplementary Figs. 10, 11). Analyses of uniparental markers (mitochondria and Y chromosome) identified a predominance of certain uniparental haplogroups in BOT (L0d), BRN (L3) and MAL (E1b1b) (Supplementary Note 1.8 and Supplementary Fig. 12) that further underlies the complex ancestral contributions to these groups.

## Revealing further genomic variation

In general, SNV discovery in the H3A-Baylor populations correlated with sample size, with between 12 and 20 million SNVs identified per population. Variant calling also revealed a total of 190,555 potentially multiallelic sites (Methods), most of which (more than 90%, *n* = 189,900) included three alleles in the dataset; the remainder of sites (*n* = 655) were biallelic in the dataset, but had a third allele in the reference genome (GRCh37). Multiallelic sites can provide unique insights into human migration and disease; however, more consistent and accurate annotation of such sites is required to capitalize on this potential^25^.

**Fig. 3:**
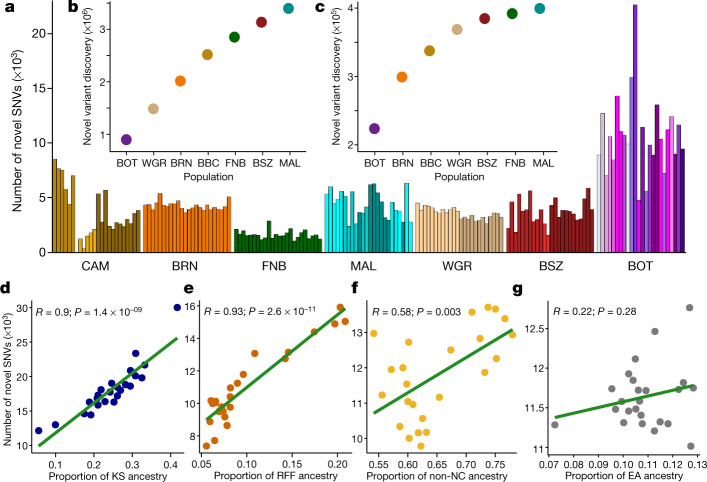
Novel variation in the H3Africa dataset. **a**, Novel variants per individual in each population (*n* = 24 biological independent samples randomly chosen from each group to match the smallest used dataset). Shading within a population reflects self-identified ethnolinguistic affiliations (Supplementary Table 3). **b**, **c**, The number of additional total (**b**) and common (**c**) variants discovered in each population starting with those identified in BOT. **d**–**g**, Correlation (Pearson, line of best fit is shown in green) between the number of novel SNVs and proportion of KS in BOT (**d**), RFF in CAM (**e**), non-NC in MAL (**f**) and east African (EA) ancestry in BRN (**g**).

In total, around 3.4 million SNVs in the H3A-Baylor dataset had not been previously reported (Methods and Supplementary Table 4). These novel SNVs accounted for 2–5% of all SNVs in each population, and, at the single-population level, the vast majority (88%) occurred once. Given the modest per-population sample sizes, however, some of these singleton variants are likely to be common at the population level, a view supported by the observation that 9–20% of population-singleton SNVs were shared with at least one other population (Supplementary Fig. 13a). Individuals from CAM had the fewest novel SNVs, whereas individuals from BOT and MAL had the most, both in absolute number and when normalized to the fewest number of sampled individuals (Fig. 3a).

To determine whether the discovery of novel variants in our dataset was saturated, we plotted the cumulative number of novel variants discovered, using BOT as our starting population. With each additional population, novel variant discovery did not reach a definite plateau (Fig. 3b), even after removing singleton novel SNVs—more than 6,000 novel SNVs were still observed between the last two populations (FNB and MAL; Fig. 3c). Given the current overrepresentation of individuals with central and west African ancestry (for example, YRI) in publicly available genomic databases, we also assessed whether novel variant discovery might be improved in ancestries that are not as well-represented. We found a strong correlation between variant discovery and the proportion of non-central-west African ancestry among our populations, particularly KS (*R* = 0.9, *P* = 1.4 × 10^−9^) and RFF (*R* = 0.93, *P* = 2.6 × 10^−11^) ancestries (Fig. 3d–g).

## Identifying new signatures of selection

**Fig. 4:**
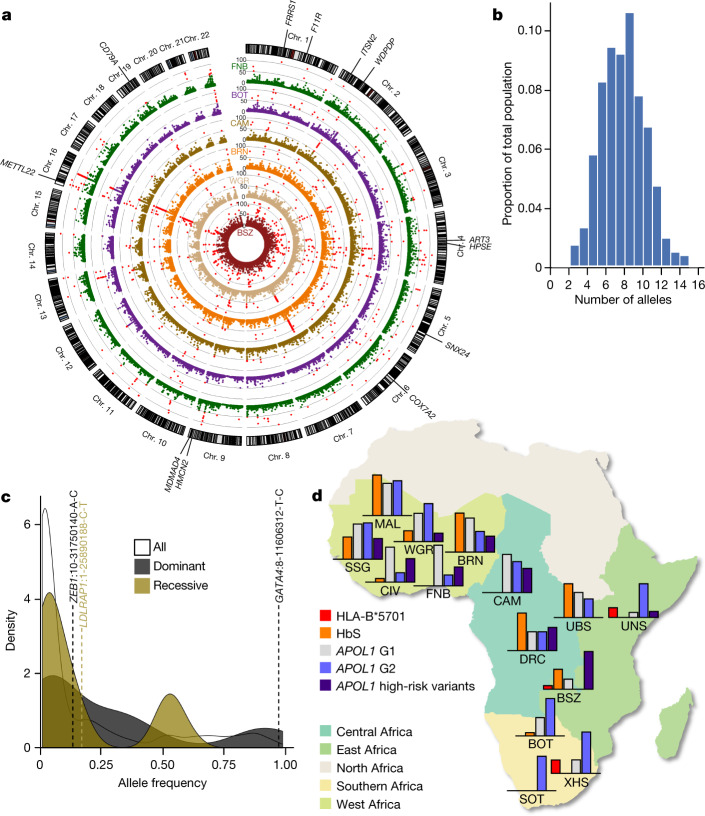
Selection and medically relevant variants in African populations. **a**, Circular Manhattan plot showing the CLR score distribution in 10-kb windows in the six HC-WGS populations (Supplementary Tables 5, 6). Loci with CLR scores > 49.5 (corresponding to a *P* < 0.001) are shown as red dots. Genes within regions with significant outlier scores in four or more groups (*FRRS1*, *ITSN2*, *WDPCP*, *SNX24*, *METTL22* and *HMCN2*) or two or fewer groups (*ART3*, *F11R*, *CD79A*, *COX7A2*, *HPSE* and *MAMDC4*) are highlighted. **b**, Burden of pathogenic (class 5) ClinVar SNVs in H3Africa cohort. **c**, Density plot of frequencies of pathogenic and likely pathogenic ClinVar SNVs (*n* = 262) differentiated by the most commonly associated inheritance pattern of the monogenic disease gene in cases in which a gene has been implicated; three variants with allele frequency > 5% are shown, illustrated as gene name:chromosome-base pair position-reference allele-variant allele. **d**, Distribution of disease alleles common to Africa across H3Africa populations. The map was created using R^43^. In each population, the corresponding bar graphs show the relative proportions of the specific disease-associated alleles (Supplementary Table 21). HbS in CAM and FNB are omitted as they include individuals with homozygous sickle cell disease (HbSS).

**Extended Data Fig. 2:**
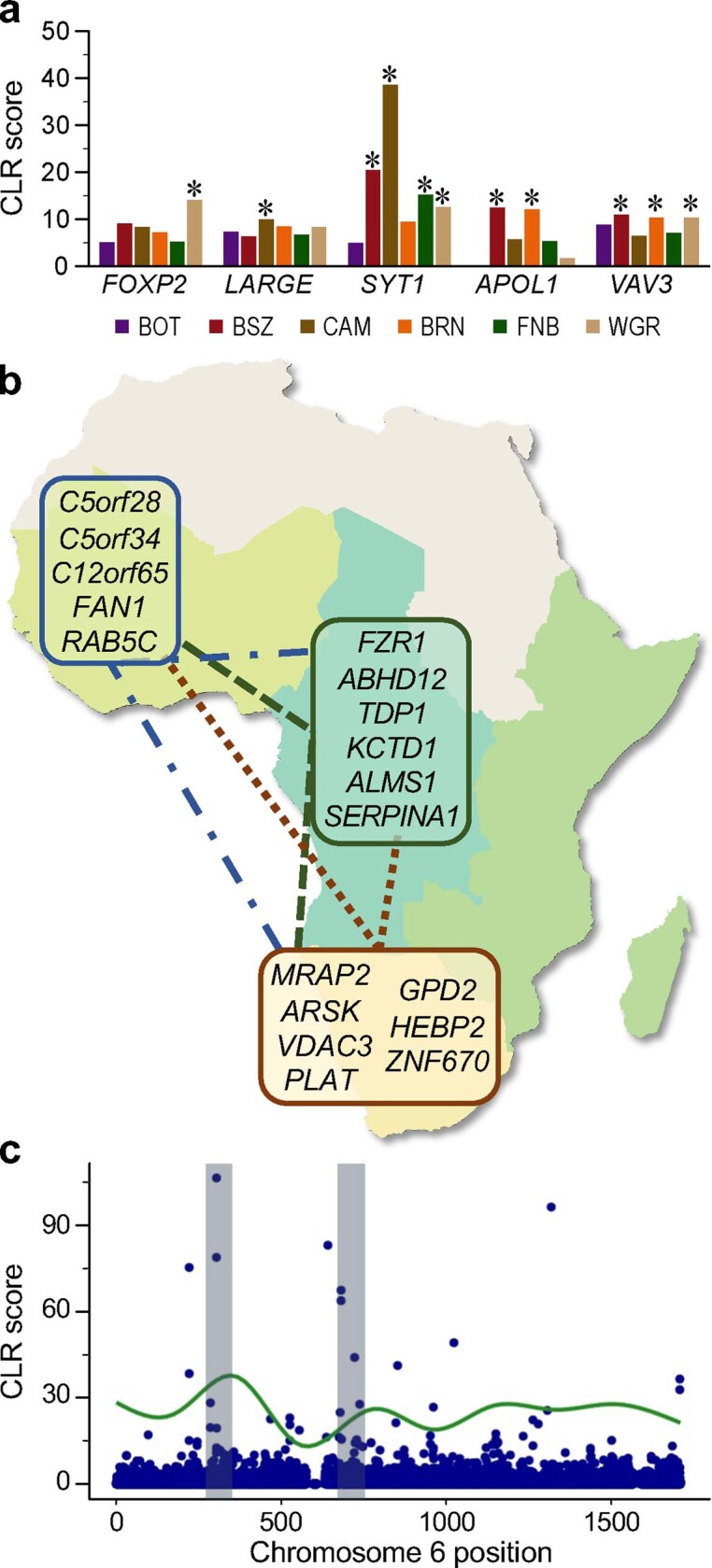
Characteristics of known and regional selected loci. **a**, CLR score distributions in known selected genes (significant population-specific outlier scores (that is, with *P* < 0.01) for the window overlapping the gene are indicated by an asterisk). **b**, Summary of PBS comparisons. Genes with longer branch lengths in WGR compared to BOT and CAM are circled in blue; longer branch lengths in BOT and CAM in comparison to the other two populations are encircled in brown and dark green, respectively. **c**, Overlap between the proportion of KS ancestry (%) and CLR score across chromosome 6 in BOT.

Adaptive selection of genomic loci in response to dietary, environmental and infectious-disease exposures, have been well-described across the continent^26^. The distribution of composite likelihood ratio (CLR)^27^ statistic scores for 10-kb windows across the genome in the six populations is summarized in Fig. 4a. Outlier windows (CLR score > 49.5; *P* < 0.001; Supplementary Table 5), which are suggestive of recent selection, were detected in each of the six HC-WGS populations. Collectively, these regions mapped to 107 genes, of which 62 (58%) were novel, and 45 were identified previously^4,9^ (Supplementary Tables 5–7). Almost half of these novel selected loci were outliers in two or more populations, which is perhaps a result of ascertaining predominantly NC populations (Supplementary Tables 5–7). However, there was still discernible heterogeneity in selective pressure between populations—only 13 loci were detected as outliers in more than four of the six populations (Fig. 4a), and some signals (for example, signals that overlap with *ART3* and *MAMDC4*; Fig. 4a) were only detected as outliers in one or two of the sampled populations. Functional annotation of putatively selected genes revealed that these genes were predominantly associated with immune-related functions (Supplementary Table 8), inclusive of genes such as *C5AR1* and *MYH10* (bacterial infection); *ARHGEF1*, *ERCC2* and *TRAF2* (viral infection) and *IFNGR2* (both viral and bacterial infection). In addition, some of the previously characterized selection signals, such as *APOL1* and *LARGE1*, were observed at a more liberal threshold of *P* < 0.01 (Extended Data Fig. 2a).

We also analysed the non-coding regions detected as CLR outliers (Supplementary Note 2.2), focusing on long contiguous stretches of signals (Supplementary Table 5), outliers that coincided with genome-wide association study (GWAS) signals (Supplementary Table 9 and Supplementary Fig. 14), and signals that overlapped with expression quantitative trait loci (Supplementary Figs. 15–17). We detected evidence for signatures of selection at several regulatory loci, including signals that are linked to traits such as chronic kidney disease, uterine fibroids and blood cell indices (Supplementary Note 2.2).

Different methods of detecting selection will identify different types and timescales of selection. When we compared regions with outlier CLR sores with those detected using an orthogonal method^28^ based on the integrated haplotype homozygosity score—which detects more-recent sweeps^29^—we found that around 20% of the CLR-identified genes were also detected in the analysis based on the integrated haplotype homozygosity score, including the immune genes *ERCC2*, *C5AR1* and *TRAF2* (Supplementary Table 10). Loci detected using both approaches included 10 previously reported genes and 11 genes for which selective sweeps are, to our knowledge, reported for the first time in this study (Supplementary Table 10).

To identify selection signals that are unique to southern African (proxied by BOT), central African (proxied by CAM) and west African (proxied by WGR) populations, we used an approach based on the population branch statistic (PBS)^30^. This analysis identified three genes that are involved in metabolism (*MRAP2*, *ARSK* and *GPD2*) among those uniquely selected in BOT (Extended Data Fig. 2b, Supplementary Note 2.3 and Supplementary Tables 11, 12) and genes that are involved in DNA maintenance among those unique to WGR (*C12orf65* and *FAN1*; Extended Data Fig. 2b and Supplementary Table 13) and CAM (*FZR1*, *TDP1* and *KCTD1*; Supplementary Note 2.3 and Supplementary Table 14).

We also found evidence for preferential gene flow from KS among the selection signals in BOT (Extended Data Fig. 2c and Supplementary Table 15, 16). CLR selection outliers *GNL1*, *MYH10* and *SMC1B*, as well as *TIGD3* and *VDAC3*—both of which were identified in the PBS-based scan—all had KS ancestry that was substantially higher than the mean genome-wide distribution of KS ancestry (+3 s.d.). Although we were unable to detect any major differences in KS ancestry at the gene-set level for either CLR- or PBS-based outlier genes, the high KS ancestry in the aforementioned genes bolsters reports of adaptive introgression in southern African selection signals^9^.

## Highly differentiated variants in African genomes

**Extended Data Fig. 3:**
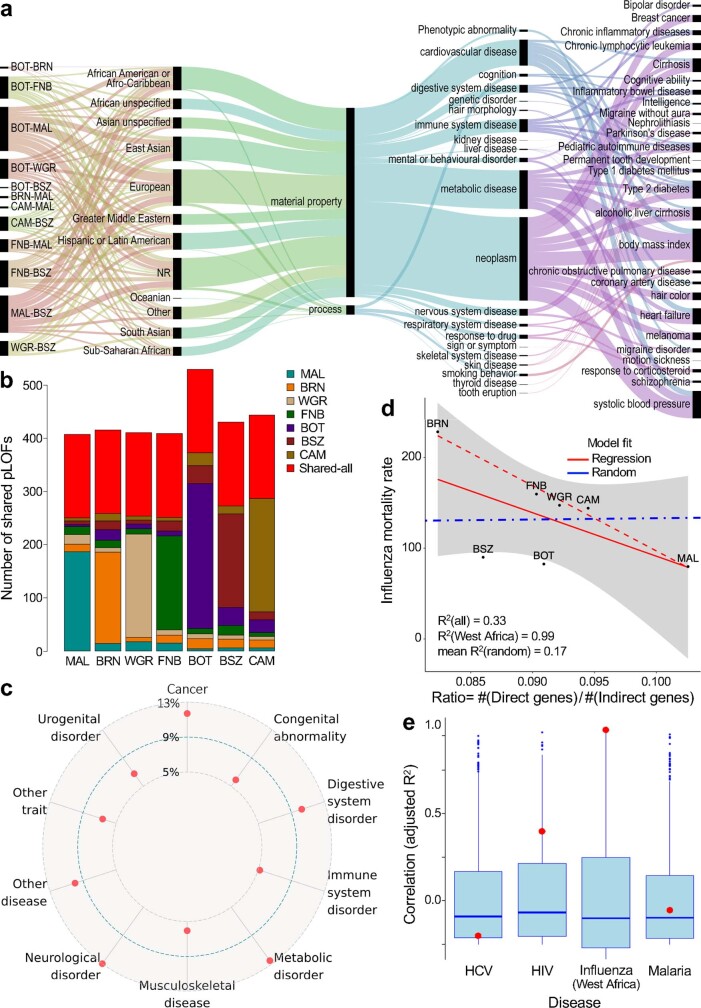
Highly divergent and putative LOF variants. **a**, EFO traits from the GWAS catalogue reflected by highly divergent SNVs within 50 kb of GWAS hits. From left to right, ribbons illustrate the relative representation of variants across pairwise population comparisons, GWAS ancestry, EFO top label, EFO trait or disease label, and disease or traits mapped to the EFO label. **b**, Distribution and sharing of common (MAF > 5%) putative LOF variants between two or more populations (coloured bars) and between all populations surveyed (red bars). **c**, Specific disease classes to which 5% or more genes with putative LOF variants shared between all populations were mapped. **d**, Correlation (Pearson) between WHO mortality rates for influenza and ratio of putative LOF variants in direct (*n* = 181) compared with indirect (*n* = 1842) influenza-associated genes (red solid line, all populations; red dotted line, west African populations). The blue dotted line represents the mean correlation for the same correlations generated using 1,000 permutations of random genes; the s.e.m. for all populations is shown in grey. **e**, Correlation statistics (adjusted *R*^2^) for the putative LOF ratio for genes related to hepatitis C (HCV, *n* = 190 direct genes, *n* = 1837 indirect genes), HIV(*n* = 724 direct genes, *n* = 1351 indirect genes), influenza in west African countries (CAM, MAL, FNB and BRN), and malaria (*n* = 484 direct genes, *n* = 1554 indirect genes) are shown as red dots against the box plot distributions of correlation statistics (adjusted *R*^2^) generated using 1,000 permutations of random genes (Supplementary Table 18). Box plots show the median value (centre line), whiskers indicate the limits of the highest (fourth) and lowest (first) quartiles of the data; distribution outliers are shown as dots.

The complex population structures and variable selection pressures observed in our dataset are known to promote differentiation in allele frequencies between populations. We therefore sought to identify highly differentiated variants (HDVs) with substantially different (more than 40%) allele frequencies between H3A-Baylor populations (Methods). For this analysis, HDVs across the β-globin gene cluster (chromosome 11) and the HLA region (chromosome 6), both of which are known to have extensive linkage disequilibrium between markers, were masked (Methods). Among the remaining HDVs (*n* = 2,497), more than 40% (*n* = 1,106) were observed between BOT (southern Africa) and MAL (northwest Africa) (Supplementary Table 17), and this geographical separation also generated the most divergent allele frequencies. Some of these HDVs probably also reflect the high proportion and historically deeper KS ancestry among BOT^31^. Empirically, 275 HDVs were located within 50 kb of genome-wide significant single-nucleotide polymorphisms (*P* < 5 × 10^−8^) in the NHGRI-EBI GWAS Catalog, and these primarily mapped to genes implicated in cardiometabolic traits, such as systolic blood pressure and type 2 diabetes (Methods and Extended Data Fig. 3a). Even though the vast majority of GWAS have been conducted in populations of European ancestry, GWAS hits proximal to HDVs were from studies that included participants of diverse ancestries (Extended Data Fig. 3a).

The site frequency spectra of variants that are predicted to be damaging and or likely to be benign in our populations (Supplementary Fig. 18) were consistent with expectations of purifying selection, but also revealed a substantive number of shared and common putative loss-of-function (LOF) variants (Extended Data Fig. 3b and Supplementary Fig. 18) of likely relevance to variant curation efforts (Extended Data Fig. 3c, Supplementary Fig. 19 and Supplementary Note 3). In addition to its role in rare Mendelian disease, putative protein-damaging variation has also been associated with common multifactorial diseases^32^. Given the burden of infectious diseases on the African continent, we explored the relationship between putative LOF variation in genes implicated in specific infectious diseases and regional differences in disease mortality as an available proxy for disease outcome. We calculated a putative LOF burden ratio for each population using putative LOF variants in genes designated as ‘directly’ (*n* = 181) and ‘indirectly’ (*n* = 1,842) implicated in influenza (Methods and Supplementary Table 18), and plotted this against the 2016 country-specific influenza mortality rates from the World Health Organization (WHO)^33^. We observed a modest inverse correlation (*R*^2^ = 0.33, Pearson’s correlation) between the putative LOF burden ratio and country-specific influenza mortality rates (Extended Data Fig. 3d) that was significantly different from random (mean *R*^2^ = 0.165, s.e.m. = 0.006, Pearson; *P* = 1.45 × 10^−102^, Wilcoxon signed-rank test; Extended Data Fig. 3d). Among west African groups, the resulting correlation was even more striking (*R*^2^ = 0.99; random iteration mean *R*^2^ = 0.235, s.e.m. = 0.008; *P* = 9.33 × 10^−302^, Wilcoxon signed-rank test; Extended Data Fig. 3d, e). A strong correlation was also seen with mortality associated with infection with human immunodeficiency virus (HIV) (*R*^2^ = 0.501, random mean *R*^2^ = 0.187, s.e.m. = 0.006; *P* = 6.86 × 10^−209^, Wilcoxon signed-rank test), but not with infection with malaria (*R*^2^ = 0.120; random mean *R*^2^ = 0.165, s.e.m. = 0.006; *P* = 0.99, Wilcoxon signed-rank test) or hepatitis C virus (HCV) (*R*^2^ = 0.0002; random mean *R*^2^ = 0.174, s.e.m. = 0.006; *P* = 1.00, Wilcoxon signed-rank test) (Extended Data Fig. 3e). Although environmental and socioeconomic factors remain important contributors to country-reported mortality, these data provide further support for a host genetic contribution to outcomes in some infectious diseases.

## Context for medically relevant variation

**Extended Data Fig. 4:**
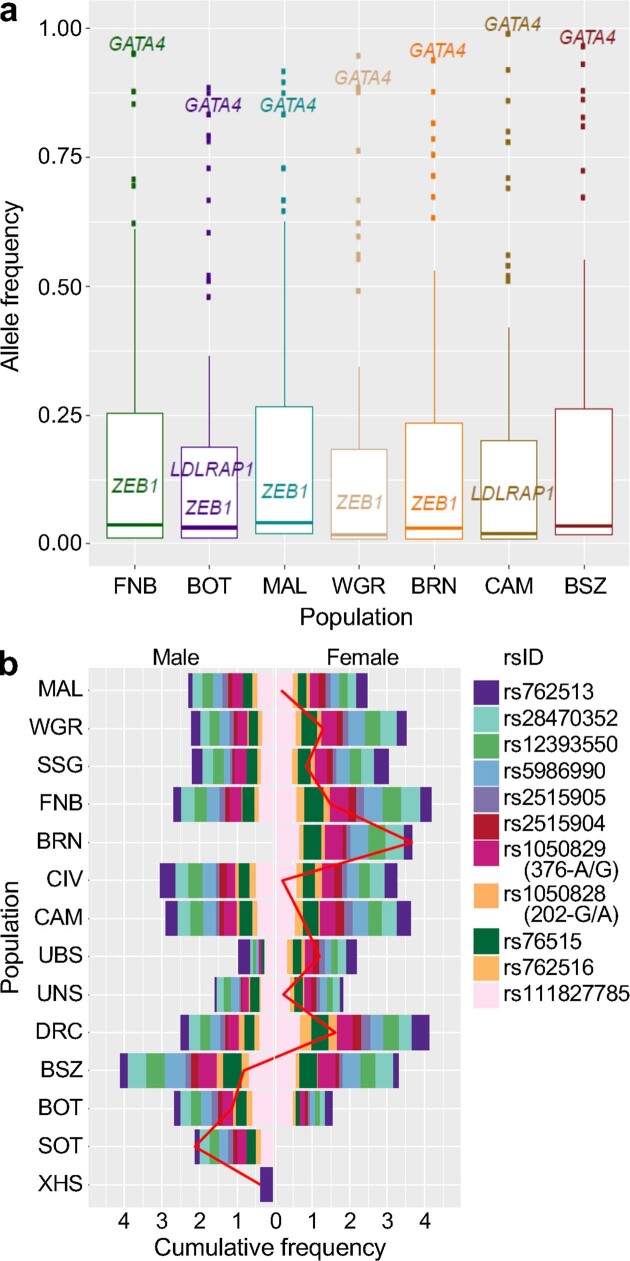
Distribution of *G6PD* variants and ClinVar pathogenic variants across H3Africa populations. **a**, Frequency distribution of pathogenic and likely pathogenic variants (*n* = 287) in H3Africa HC-WGS populations. Disease genes with variants that had an allele frequency > 5% across multiple populations (shown in Fig. 4c) are highlighted. Box plots show the median value (centre line), whiskers indicate the limits of the highest (fourth) and lowest (first) quartiles of the data; distribution outliers are shown as dots. **b**, Relative frequencies of 11 *G6PD* deficiency-associated alleles within each population separated by sex. *G6PD* A− 202A and 376G refer to the A-deficiency associated with either rs1050828 (c.202G>A) or rs1050829 (c.376A>G) (MIM 305900).

To provide a more global context for medically relevant genetic variation, we annotated our dataset with the American College of Medical Genetics and Genomics (ACMG) Secondary Findings gene panel (ACMG 2.0) of reportable variants in 59 genes^34^. Only eight individuals carried any reportable ACMG variants, and these were limited to singleton variants (one per participant) in *TINF2*, *KCNQ1* and *RYR1* (associated with dominantly inherited disorders), as well as *ATP7B* and *PKP2* (associated with recessively inherited disorders). By contrast, almost everyone in our HC-WGS cohort carried at least one variant designated as ‘pathogenic’ (level 5) in the ClinVar Database (v.20181028) (http://www.ncbi.nlm.nih.gov/clinvar/), with each person carrying a median of 7 (range, 2–14) alleles (Fig. 4b and Supplementary Table 19). Among the 262 unique variants annotated as pathogenic or likely pathogenic (level 4), around 21% (54 out of 262) had a minor allele frequency (MAF) > 0.05 in at least one HC-WGS population (Fig. 4c and Supplementary Table 20) and, of these, 13 (4.9%) had a MAF < 0.05 across all population groups in gnomAD (Extended Data Fig. 4a and Supplementary Note 4). We interpret this finding as most suggestive of variant misclassifications in ClinVar and other databases.

Finally, we surveyed the frequency of select, well-described, disease-associated alleles commonly found in populations of African descent (Methods). Consistent with expectations for a locus that protects against malaria mortality, estimates of the *G6PD* A− 202A and 376G alleles were generally consistent with the distribution of endemic malaria across the continent. Eleven single-nucleotide polymorphisms in *G6PD* associated with protection against severe malaria in heterozygotic female participants^35^ also showed similarly divergent frequencies across our populations (Extended Data Fig. 4b). Similarly, the common sickle cell disease mutation (rs334; HbS; MIM 603903) was found at typically high allele frequencies in malaria-endemic west and east African populations^36^ (Fig. 4d and Supplementary Table 21). HbS frequencies ranged from 10% (BRN) to 19% (SSG), but was nearly absent from south African (BOT, XHS and SOT) genomes. Notably, HbS showed widely divergent allele frequencies in the two populations from Uganda, with the Bantu-speaking (UBS) population having one of the highest frequencies (20%) whereas the allele was not observed in the UNS population.

*APOL1* G1 and G2 alleles in the homozygous or compound heterozygous state (G1/G1, G1/G2 or G2/G2) confer protection against *Trypanosoma brucei gambiense* infection but also increase susceptibility to nephropathy in non-trypanosomiasis endemic areas, especially in the presence of HIV infection^12^. G1 and G2 allele frequencies varied widely across our populations, and were the highest among west African groups (for example, the G1 frequency was 43% in CIV and 34% in FNB), but were significantly lower elsewhere on the continent (Fig. 4d). This is consistent with previous reports of geographical correlation with the prevalence of sleeping sickness^12^. The frequency of G1 was also highly differentiated between the two Uganda population groups (UBS, 14%; UNS, 2%)^37^, and this was in contrast to the frequencies of G2 in the two groups (UBS, 10.6%; UNS, 11.5%). The overall frequency of the *APOL1* risk genotype (that is, the recessive state for the diplotype: G1/G1, G1/G2 or G2/G2) varied substantially among the groups in which it was present (Fig. 4d and Supplementary Table 21), suggesting that the risk of *APOL1*-associated nephropathy is appreciably high across the continent, with the highest burden in west Africa.

Previously, the Maasai in Kinyawa, Kenya^38^ were the only African population noted to have an appreciable frequency of HLA-B*5701, which mediates hypersensitivity to the antiretroviral drug abacavir. In our expanded dataset, the allele was absent from west African populations, but observed in BSZ (1.5%), UNS (3.3%) and XHS (6.3%) (Supplementary Table 21), at frequencies typically observed in populations of European or Asian ancestry.

## Discussion

This study represents one of the most-extensive studies of high-depth-sequenced African genomes to date. We deliberately focused on SNVs—which could be confidently inferred—but a similar wealth of diversity and novelty is likely to be found within other variant classes. Despite drawing individuals from ongoing genomics studies on the continent, care was taken to avoid possible systematic biases and the patterns of variation are thus expected to be largely representative of the respective groups.

Our results reveal a genetic continuum of NC speaker populations across the continent and extend our current understanding of the routes, timing and extent of the Bantu migration—the defining demographic event of African genetic diversity. The proposed route overlaps with the spread of the Kalundu pottery tradition, which has also been associated with the Bantu expansion in these regions^39,40^. However, the estimated dates for the spread of Kalundu pottery predate our admixture dates, and the association between this tradition and Bantu migration has been questioned, leaving the proposed parallels between the archaeological and genetic migrations unresolved.

Nigeria, in keeping with its tremendous linguistic diversity, is currently the best-represented African country in terms of genomic data. Our observations of substantial NS admixture in the Berom, and both HDVs and novel variation in both NC and non-NC speakers, suggest that Nigerian populations in existing public databases are not only likely to underrepresent the genomic diversity of Nigeria, but are almost certainly poor general proxies of African continental groups. Additional deep sequencing in multiple African populations will be needed to provide a more-comprehensive compendium of variation across the continent.

Viral epidemics, including outbreaks of HIV, Ebola and Lassa fever, have been reported across Africa. Against this backdrop, our observations of selected loci that overlap genes that are important to viral infection support the potential for a hitherto undescribed role for resistance and/or susceptibility to viral infections in shaping the genomes of human populations across Africa. This was partially bolstered by strong correlations between putative LOF variants in genes implicated in influenza and HIV and their respective disease mortalities, although the latter observations require replication and confirmatory analyses in cohorts of individuals with the diseases. Alongside immune genes, we also observed positive selection in genes associated with DNA repair, reproduction, and carbohydrate and lipid metabolism, as well as geographical-region-specific positive selection in genes such as *PLAT* and *SERPINA1* within NC speakers.

The combined effect of ancestral events and exposure to infectious agents on the diversity and variation of African genomes was perhaps best exemplified by the stark allele frequency differences observed between the UNS and UBS groups from Uganda. Despite their shared geography, the two groups varied significantly in the frequencies of three of the four medically relevant variants surveyed, including HbS and *G6PD* alleles. These two loci are known to be protective against severe malaria, which is endemic in Uganda. Historically, differences in HbS frequencies between these groups were attributed to the relatively recent spread of malaria to NS populations; however, our results, and other recent findings^41^, suggest that recent migration from northern regions, where malaria is less common (UNS), as opposed to from malaria-endemic western regions (UBS) is another plausible explanation^42^. A similar argument can be made for the *APOL1* high-risk G1 and G2 alleles, which have been shown to provide protection against specific *Trypanosoma* species^12^. These alleles are commonly observed in trypanosomiasis-endemic regions such as Uganda and west Africa (UBS), but less so in northeastern geographies including Sudan (UNS). Similarly, HLA-B*5701 was previously only observed among northeast African populations; its high frequency among the UNS, yet absence from the UBS, probably also reflects this ancestral divergence.

Our findings indicate that the implementation and use of genome-level sequence data in Africa will require a broadly ascertained and comprehensive compendium of variation, alongside high-level curation of variants. African genome variation is likely to be a better representation of variant distribution for both African diaspora and global populations, and, therefore, a full repertoire of African genomic variation could provide a better genomic reference for both medical and population genetics. The data generated have facilitated the development of a microarray genotyping chip and imputation panel, and are being made available to researchers in the field (see Methods, ‘Data availability’) as important springboards for future studies of demography, migration, ancestry and genetic variation in Africa.

## Methods

### Data reporting

No statistical methods were used to predetermine sample size. The experiments were not randomized and the investigators were not blinded to allocation during experiments and outcome assessment.

### Samples, datasets and sequencing platforms

The primary analysis datasets were derived from three sources: The H3Africa Consortium (referred to as H3A-Baylor)^8^, The TrypanoGEN Collaborative Centre of the H3Africa Consortium (TrypanoGEN)^18^ and the SAHGP^17^. All samples were collected after appropriate approvals had been obtained from local Ethics Boards and Committees in each of the represented countries, and participants gave informed consent (see Supplementary Note 5 for details). Sequencing was performed on Illumina platforms and—after alignment and baseline quality control to account for the different platforms and coverage depths (Supplementary Methods Table 2)—data were combined to create a single merged file (Supplementary Methods Fig. 1) to facilitate downstream analyses.

#### The H3Africa Consortium

The principal investigators of each of the 19 projects funded during the first 5-year funding cycle of H3Africa were invited to submit samples for WGS, provided the existing consent for recruited individuals included the broad use of samples for WGS, and the project had existing ethics approval for such a study. In addition, a broader request was made to consortium researchers with other samples from African populations that would be similarly eligible for inclusion, even if they were not recruited through a formal H3Africa project. A total of 519 samples from 8 projects were submitted for consideration. Submitted samples were predominantly ascertained from control datasets recruited from the respective studies, with the exception of samples from case-only studies in Cameroon, Botswana, Mali and Benin (Fig. 1 and Supplementary Methods Table 1). These samples were prioritized to include population and ethnolinguistic groups that had not previously been sequenced. Samples were shipped to the Human Genome Sequencing Center at Baylor College of Medicine, Houston, USA, under signed material transfer agreements from each project. A total of 348 samples were prepared using the TruSeq Nano DNA Library Prep Kits and underwent WGS on an Illumina X-Ten to a minimum depth of coverage of 30×. The resulting dataset was labelled H3A-Baylor (Supplementary Methods Table 2).

#### The TrypanoGEN Collaborative Centre of the H3Africa Consortium

The TrypanoGEN project is a collaborative centre funded by the Wellcome Trust to study the host and parasite genetics and genomics of trypanosomiasis across Africa^18^. Ethical approval for the use of samples for genomic studies of trypanosomiasis was obtained in participating countries and informed consent for genomic studies and sharing of data with researchers working on other diseases was obtained from participating individuals. A total of 300 individuals were recruited from five countries (Uganda, Zambia, the DRC, Cameroon and Côte d’Ivoire) of which 200 had been sequenced for inclusion in this project (Supplementary Methods Table 2). DNA was extracted from blood samples in the respective countries of collection with the exception of samples from Guinea and Côte d’Ivoire, for which DNA extraction was performed at CIRDES in Burkina Faso. WGS was subsequently performed on an Illumina Hiseq 2500 to an average depth of 10× using the Illumina TruSeq PCR-free kit at the Centre for Genomic Research, University of Liverpool, UK.

#### SAHGP

SAHGP is a multi-ethnic project to investigate the genomic diversity of the peoples of Southern Africa and build genomics capacity in that region (https://sahgp.sanbi.ac.za/)^17^. The use of SAHGP samples for this study was approved by the Human Research Ethics Committee (HRECΞMedical) of the University of the Witwatersrand, Johannesburg (protocol number: M120223). Three groups of participants were enrolled and venous blood was collected using EDTA tubes. Inclusion criteria were as follows: male, over the age of 18 years, four grandparents who speak the same language as the participant, not known to be related to the other participants in the study and willing to provide broad informed consent (including consent to share data and DNA for future studies approved by the HREC (Medical)). Two main Bantu-speaking ethnolinguistic groups were included: the Sotho (Sotho–Tswana-speaking individuals; *n* = 8) were recruited from in and around the town of Ventersburg in the Free State Province and the Xhosa-speaking individuals (Nguni language; *n* = 7) were recruited from the Eastern Cape Province. One individual spoke Zulu (Nguni language) and was from Johannesburg. The DNA samples were normalized to around 60 ng μl^−1^ and approximately 5 μg DNA was submitted to the Illumina Service Centre in San Diego, USA, for sequencing on the Illumina HiSeq 2000 instrument (around 100-bp paired-end reads, about 314-bp insert size) with a minimum read depth of coverage of 30×.

### Data processing and merging

See Supplementary Table 23 for a full list of references for databases and software used.

#### Alignment and pre-processing of reads

Raw FASTQ reads generated by sequencing were mapped to the human reference genome GRCh37 (also known as hs37d5) using the BWA-MEM algorithm of the BWA software package^44^. Optical and PCR duplicate reads were marked with Picard MarkDuplicates on a per-sample basis^45^ and reads were sorted by coordinate using SAMtools v.0.1.19^46^.

#### Quality control before variant calling and BAM file augmentation

Before variant calling, the percentage of aligned reads was found to be 90% or greater in each sample. The resulting BAM files were merged on a per-sample basis and these sample-level BAM files were recalibrated using GATK^45^. This process consisted of a per-sample realignment of reads around known and discovered insertions and deletions (indels) using the known indels from the gold datasets of the Mills Devine and 1000 Genomes Project (1000G), as well as the low-coverage dataset of 1000G phase 1. GATK (v.3.3-0) RealignerTargetCreator and IndelRealigner were used for alignment, in addition to base quality score recalibration with GATK BaseRecalibrator and PrintReads (using known variant sites from dbSNP v.138 and the same indels used in local realignment). SAMtools was used to generate a base quality score and MD tag (that is, a string describing the mismatching positions of a read to the reference used for reference-free SNV and indel calling), which helped to improve calling quality. Additional quality checks for cross-sample contamination were performed using VerifyBamID from 1000G Omni2.5 VCF, requiring that the calculated FREEMIX was less than 0.05. One H3A-Baylor sample did not pass these quality controls and was not included in the downstream analysis.

#### Variant annotation

Variant annotations were obtained using SnpEff (version 4.3-3) (Supplementary Table 23) with human genome build GRCh37.75 (October 2016). We used the SnpEff default parameters including the -lof argument to annotate for LOF and nonsense-mediated decay predictions. We also included annotations for variant labels from dbSNP (v.150), for clinical importance from ClinVar Database (v.20181028) and for GWAS hits from the GWAS catalogue (v.2019-10-14) (Supplementary Table 23). In addition to functional annotations, variants were also annotated for allele frequencies in the 1000G, ExAC (r.2.0.1), gnomAD (v.2.0.2), African Genome Variation Project (AGVP)^4^, SAHGP and TryopanoGEN datasets.

#### Variant discovery

The choice of software for variant calling of both low- and high-depth of coverage WGS data was based on the evaluations of the AGVP^4^. We used HaplotypeCaller (Supplementary Table 23) to call per-sample SNVs and indels from SAHGP, H3A-Baylor and TrypanoGEN datasets in gVCF mode. As the TrypanoGEN dataset had a lower depth of coverage (around 10×), a minimum confidence threshold at which variants were called and included was set to 10; for both of the datasets with a high depth of coverage (SAHGP and H3A-Baylor), a minimum confidence threshold at which variants were called and included was set to 30. Joint variant calling was done on each dataset using GenotypeGVCFs in GATK.

#### Variant filtering of autosomal genes

Variant quality score recalibration (VQSR) was performed for each dataset separately. SNVs were filtered using VariantRecalibrator and ApplyRecalibration in GATK. For SNVs, we used overlapping sites from HapMap III and 1000G phase 1 Omni2.5 sites as truth and training sets (prior probabilities of 15 and 12, respectively for HapMap III and 1000G phase 1 Omni2.5 sites). High-confidence 1000G phase 1 SNVs were used as an additional training set (prior probability of 10). dbSNP v.138 was used as a set of known sites (prior probability of 2). To build the VQSR Gaussian mixture model, we used annotations at each site related to coverage (QD (QualByDepth) and DP—where DP is the approximate read depth after filtering reads with poor mapping quality and bad mates and QD is the variant confidence normalized by the unfiltered depth for the variant allele); strand bias (FS (FisherStrand) and SOR (StrandOddsRatio)—where FS is a Phred-scaled *P* value using Fisher’s exact test and SOR is the odds ratio of a 2 × 2 contingency table of positive/negative strand and reference/alternative allele), mapping quality (MQ, MQRankSum and ReadPosRankSum—where MQ is the root mean square of the mapping qualities), which serves to average across reads and samples; MQRankSum is the *Z*-score from a Wilcoxon rank-sum test of alternative versus reference mapping qualities; and ReadPosRankSum is the *Z*-score from a Wilcoxon rank-sum test of alternative versus reference read-position biases) and likelihood-based Hardy–Weinberg equilibrium tests (InbreedingCoeff). The resulting receiver operating characteristic curves were filtered by the variant quality score log odds ratios calculated by VariantRecalibrator, and all SNVs below the VQSLOD threshold of 99.5% were removed. To facilitate faster joint calling between high- and low-coverage datasets, a union of all of the high-coverage SNV sites of each individual dataset was created. As SNVs in the individual datasets were already ‘known’ from previous alignments, joint variant calling was done across all the datasets only at the union using Genotype gVCF in GATK to create a merged final dataset (Supplementary Methods Fig. 1). For the sex and mitochondrial chromosomes, the X chromosome followed the same VQSR filter approach as the autosomes; Y and mitochondrial chromosomes, however, were not filtered using the VQSR model. The pseudoautosomal regions on the X chromosome were called as diploid and other X chromosome regions were called haploid in female participants. The Y chromosome pseudoautosomal regions were called diploid and the remaining regions were called haploid in male participants. The mitochondrial chromosomes were called as diploid for ease of processing.

#### Multiallelic variants and haplotype phasing

After curation of individual (unphased) VCF files from each dataset and the subsequent merged dataset, data for each chromosome were independently phased to provide two haplotypes per individual. We first opted to decompose multiallelic variants in the VCF file before phasing as follows: (1) decomposing the VCF file such that variants with multiple alleles were expanded into distinct variant records—one record for each reference/alternative allele combination; and (2) normalizing the decomposed VCF file so that variants were represented using the most-parsimonious alleles from the human genome reference (GRCh37).

Combining (1) and (2) resulted in some genotypes being split over two VCF records, such as missing/alt1, missing/alt2 or ref/alt1 and ref/alt2. To improve the accuracy of low-pass and low-coverage whole-genome data, we leveraged population linkage disequilibrium, haplotype information and genotype likelihoods from initial calls using Marvin^47^ (Supplementary Table 23), with default parameters to perform the genotype refinement at sites shared by multiple individuals. This approach has been used for the 1000G^2^. We conducted further quality control on autosomal chromosomes by removing individuals and sites with high missingness (>5%); this resulted in the removal of 17 samples and around 500,000 sites. Checks for heterozygosity or relatedness were left for downstream analysis. Owing to the unavailability of haplotype scaffold panels, we independently phased and inferred haplotypes without reference haplotypes using both Eagle2.0^48^ and SHAPEIT2^47^(Supplementary Table 23). We also enabled SHAPEIT2 to produce the graph structures on which to generate the final phased haplotypes; this resulted in a pair of phased haplotypes per dataset. For each pair of haplotypes, we compared sites discordant between haplotypes generated by SHAPEIT2 (91.2%) versus Eagle2.0 (98.6%) and the VCF file before phasing. Because the estimated switch error in phasing was lower in Eagle (0.26%) than SHAPEIT2 (0.71%), we opted to use the Eagle phase panel as the default for downstream analyses.

All downstream analyses were carried out on biallelic sites only, but we did investigate multiallelic sites to gain an appreciation of their relative abundances and patterns of variation. Multiallelic variation is a largely unexplored topic in genome surveys, in part because it is difficult to discern between true multiallelic sites and sequencing errors. For this reason, we imposed fairly stringent measures to conservatively call such sites. We focused on multiallelic sites from the high coverage H3A-Baylor dataset, and discarded multiallelic sites embedded in repetitive regions or regions adjacent to known copy-number variation, as well as those for which the third allele was observed in fewer than six reads.

### Data analyses

The resulting ‘clean’ dataset was analysed by teams arranged around four main study areas: (1) population structure and admixture; (2) signatures of selection; (3) rare variation; and (4) medically relevant genes and variants. Each of these study areas are described in more detail below. Studies of selection and rare variation were limited to the 314 individuals in the H3A-Baylor dataset that had sequencing data with a high depth of coverage to improve calling confidence for rare sequence variants. The full dataset was used for the remaining analyses (population structure and admixture, and medically relevant variants). See Supplementary Table 23 for a full list of references for databases and software used.

### Population structure and admixture

#### Primary datasets

The primary datasets for the population structure and admixture analyses included WGS data generated from the merged H3A-Baylor–TryopanoGEN–SAHGP dataset (hereafter referred to as the joint dataset; Supplementary Table 22).

#### Sample-level quality control

The initial dataset consisted of 564 individuals, and these were subjected to additional quality control: we excluded individuals with >1% missing data (*n* = 86—all from the TrypanoGEN dataset) and identified duplicate samples and familial-related samples using the identity-by-descent (IBD) approach in PLINK (v.1.90, http://www.cog-genomics.org/plink/1.9/)^49^. IBD was calculated after removing SNVs in strong linkage disequilibrium by pruning SNVs with *r*^2^ > 0.15 within a window of 1,000 bp. A total of 93 individuals with PiHAT > 0.25 with at least one other sample (Mali, 57; Uganda, 2; Zambia, 19; Mossi, 3; Fon, 12) were identified; a subset of 51 unrelated individuals were randomly selected for inclusion from the pairs of related individuals (Mali, 26; Uganda, 2; Zambia, 12; Mossi, 2; Fon, 9).

#### SNV level quality control

Of the initial 41,645,936 SNVs, we removed 1,801,483 with call rate <99%; 84,758 SNVs with a significant deviation from Hardy–Weinberg equilibrium (*P* < 1 × 10^−6^); 25,352,806 SNVs with MAF < 0.01; and all A/T and C/G (ambiguous) SNVs, to facilitate merging with additional datasets (see below). After sample- and SNV-level quality control, a total of 426 individuals and 14,406,889 SNVs were available for analyses.

#### Additional datasets

The cleaned joint dataset was merged with data from four additional African datasets (Supplementary Table 22). We applied the same individual and SNV quality control parameters as above to each of the additional datasets, retaining SNVs that were common to all datasets in subsequent merging. To maximize the number of SNVs available for each of the subsequent analyses, we merged a variety of different populations for the various studies outlined below.

#### Principal component analysis

For the initial principal component analysis (PCA), we merged all currently available WGS data for African populations regardless of depth of coverage (Supplementary Table 22). We performed linkage disequilibrium pruning on our merged dataset using PLINK (v.1.90)^49^ to remove correlated (*r*^2^ > 0.15) single nucleotide polymorphisms (SNPs) in a 1,000-SNP window, advancing by 10 SNPs at a time. The pruned dataset contained 1,013,758 SNPs and 1,253 individuals with a genotype call rate of 99.9%. We used the smartPCA program from EIGENSOFT^50^ to perform PCA on the pruned dataset and the Genesis software^51^ for PCA visualization (Supplementary Table 23).

#### Admixture analysis

For the admixture analysis, in addition to the African WGS data, we merged our joint dataset with existing African genotyping array datasets (Supplementary Table 22). We carried out quality control and pruning of the merged dataset as described above. We ran ADMIXTURE (v.1.3.0)^19^ 50 times with a random seed for each value of *K* from 2 to 15. We generated ADMIXTURE cross-validation error estimates to determine the optimal value of *K*. Admixture runs were merged and summarized using both the FullSearch and Greedy algorithm and G pairwise similarity statistic in CLUMPP (v.1.1.2)^52^. The results of the greedy algorithm are shown. The Genesis software was used for PCA and admixture visualization of this dataset.

#### Procrustes and *F*_ST_ analyses

We used *F*_ST_ to estimate the pairwise distance between the various African populations (Supplementary Fig. 3), and then implemented PROCRUSTES^53^ using an in-house-generated script (see ‘Code availability’) to evaluate the correlation between geographical distances and *F*_ST_-based genetic distances. We used the function distVincentyEllipsoid in the R package geosphere (v.1.5-7) to estimate great circle distances between the geographical midpoints of those countries for which individuals were recruited from across the country, and regional or city midpoints for recruitments that were limited to a specific geographical location. *F*_ST_ was estimated using smartPCA for the joint dataset in EIGENSOFT^50^, principal coordinate analysis was performed on the geographical and genetic distance matrices, and then the test statistic was constructed as described previously^53^. Finally, we established the null distribution by randomly permuting the labels on the *F*_ST_ matrix. For the test of all populations, we generated 1 x 10^7^ permutations; for the test of only NC speakers, we generated 1 × 10^9^ permutations. Permuted distributions were used to derive empirical *P* values.

#### Testing for the presence of admixture

We next tested for the presence of admixture. To further explore and corroborate the admixture events observed in the previous approaches we used a formal test for admixture using the *f*_3_-statistic. As we were interested in exploring admixture events on a regional scale, and to maintain the highest resolution in terms of the number of SNPs, we generated four datasets for this analysis. The datasets represented population groups from west, central-west, east and south Africa; *f*_3_-statistics were generated for all possible combinations of populations using both TreeMix threepop (v.1.13)^54^ and Admixtools qp3pop (v.1.0)^20^. In both cases, a negative *f*_3_-statistic coupled with a corresponding high negative *z*-score was considered to be supporting evidence for the admixture event.

#### Admixture dating

To provide further insights into the historical context of the admixture events, we attempted to date some of the events based on the four regional datasets described in the previous section. We used MALDER (v.1.0) (https://github.com/joepickrell/malder)^55^—a modified version of ALDER^22^—which is able to predict the occurrence of multiple admixture events in a test population. We tested for specific admixture events between our joint population dataset and additional reference populations informed by the results from the admixture analysis. The minimum genetic distance to start curve fitting was set to 0.005 cM to account for short-range linkage disequilibrium between African populations, together with the (Rutgers v.3) recombination genetic map. Significant results were assessed based on the amplitude of the fitted linkage disequilibrium curves and the corresponding *z-*scores.

#### IBD sharing distance

To investigate the distribution of IBD segments shared between the different NC populations, we used the program Refined IBD^56^ in Beagle 4.1 (Supplementary Table 23). The merged dataset (consisting of 590,914 SNPs and 396 individuals) used in this analysis included select populations from Trypanogen, H3A-Baylor, and two previously published studies^9,14^. Default refined IBD parameters were used to estimate the shared IBD segments between pairs of individuals. The IBD segments were further filtered by implementing the program merge-ibd-segments, to remove breaks and short gaps in IBD segments (>0.6 cM in length). The output of merge-ibd-segments was used to compute the average pairwise IBD sharing between the different NC groups by using the previously described expression^57^.

#### Admixture masking

To identify the contribution of non-Bantu-speaking ancestry in the observed population structure, we estimated non-NC local ancestry in BOT using an estimate from RFMix_v2^58^. Phasing of the dataset was done using the Sanger imputation server (https://imputation.sanger.ac.uk/) and the African Genome Resource reference panel. Data from the Juǀ’hoansi, the Gǀwi, Gǁana and baKgalagadi, the ǂKhomani and Karretjie populations^14^, were used as the KS source, YRI as the Bantu-speaking source and CEU as the Eurasian source (parameters used: -forward-backward -e 2). Regions with high KS ancestry (>20%, and at least 25 SNPs) in BOT were identified and masked from the full dataset, after which the PCA was regenerated. Similarly, we identified and masked regions with more than 20% east African ancestry in BRN (identified using Tubu from Chad^59^ as the east African source population) and repeated the PCA. As the number of SNVs included in the analysis had the potential to affect the principal component estimates, we thinned the whole dataset to 150,000, linkage-disequilibrium-pruned SNVs for comparisons with the masked datasets.

#### Admixture model testing

We next tested the admixture model. We used qpGraph^20^ to test various alternative models of gene flow to identify the best possible central African NC-speaker population for admixture in southern and east African populations (see Supplementary Note 1.3 for details.)

#### Mitochondrial and Y-chromosome haplogroups

We then analysed the mitochondrial and Y-chromosome haplogroups. Haplogrep2^60^ was used to identify mitochondrial haplotypes for each individual. Y-chromosome haplogroup analysis was done using the AMY-tree algorithm and tool^61^. For each sample, the variants detected from the WGS VCF files were extracted and converted into the correct format before input into the AMY-tree program.

#### Runs of homozygosity

We also investigated runs of homozygosity (autozygosity). For the identification of runs of homozygosity (ROHs), PLINK v.1.9^49^ was used with the following parameters: a minimum of 100 SNVs with at least one SNV per 50 kb on average and a maximum of 1 heterozygous call and 5 missing calls. A window size of 100 kb was used to scan for ROHs across the genome. Following a previously published approach^62^, the ROH segments—depending on genomic length—were separated into three classes: short ROHs (<500 kb, class A), which most likely represent homozygosity for ancient haplotypes; intermediate ROHs (500 kb–1.5 Mb, class B), which are most likely the result of distant relatedness within a population; and long ROHs (>1.5 Mb, class C), which are suggestive of assortative mating. To provide a comparison of the ROH distribution across Africa in addition to the seven populations from our study, five African populations (YRI, LWK, ESN, MSL and GWD (see Supplementary Table 22 for definitions)) from the 1000G dataset and populations (BAG, ZUL and Ethiopian) from AGVP were included in the analysis. As the number of samples for both 1000G and AGVP datasets were around 100 per population, we randomly downsampled each population for these two datasets to 50 individuals per population. We generated an additional dataset with the modified PLINK parameter set (--homozyg-kb 300 and --homozyg-window-het 3) for better homogenization of the combined datasets with low and high depths of coverage. FHAT1 and FHAT2 were also estimated using PLINK v.1.9 with default parameters.

### Signatures of selection

#### Datasets

Our dataset included samples with different sequencing depths, which can adversely affect nucleotide diversity and allele frequency estimates^63^; therefore, the identification of signatures of selection was limited to samples from the H3A-Baylor dataset with a high depth of coverage. Samples that appeared as outliers for each population in the PCA and outliers in the full dataset were removed. Similarly, related individuals identified using the method described above were also excluded. Owing to a smaller sample size and high within-group diversity, the MAL group was excluded from this analysis. Genes in selected regions were identified using the Ensembl database^64^ and assessed for (predicted) functional impact using the Ensembl, OMIM^65^ and GeneCards databases^66^ (Supplementary Table 23).

#### CLR scores

CLR scores were calculated using SweepFinder (implemented in SWEED)^27^ for 10 kb non-overlapping sliding windows in each population. Genomic regions which have been previously shown to produce false-positive hits in WGS data (a custom list based on https://sites.google.com/site/anshulkundaje/projects/blacklists and a previously published study^67^) along with a 1-Mb flanking sequence on either side were excluded to minimize the effect of sequencing-related artefacts. To identify a threshold for identifying extreme outliers, we randomly sampled 10,000 10-kb regions from the 6 populations. On the basis of the distribution of CLR scores in this set, we identified CLR scores > 49.5 to correspond a *P*-value cut-off of *P* < 0.001 and took this as our significance threshold.

#### Integrated haplotype homozygosity scores

Integrated haplotype homozygosity scores (iHS) for SNVs with MAF > 0.05 were estimated using SelScan^28^ in each population. For each population, the scores were then normalized across 40 allele frequency bins. As advised in previous analyses^68,69^, instead of focusing on maximum iHS variants, we aimed to identify genomic regions with the highest fraction of extreme iHS-containing variants. For this, based on the background distribution of the normalized iHS scores in all of the populations we identified |iHS| > 2.6 to correspond to *P* < 0.01. For each 10-kb window that was scanned for the CLR analysis in a population, we measured the percentage of SNPs with outlier iHS scores (|iHS| > 2.6). The top 1% of windows with the highest percentage of outlier iHS score were considered to be outlier windows for each population.

#### PBS analysis

For the PBS analysis, we used WGR as the representative west African population, CAM as the representative central-west African population and BOT as the representative south African population. *F*_ST_ scores for exonic SNVs with MAF > 0.01 in the dataset were estimated between pairs of the representative populations as well as with CHB (downsampled to 50 individuals) from the 1000G dataset using VCFtools^70^. We then used a previously published method^30^ to estimate population PBS between WGR and BOT, between BOT and CAM, and between WGR and CAM. The SNVs with highest branch lengths (*P* < 0.001) in a population compared to the other populations (one at a time) were considered as signals.

#### Integration with GTEx

Analyses for the integration with the Genotype-Tissue Expression (GTEx) dataset were performed as follows. Chromosomal positions for selected loci falling in non-coding regions were intersected with significant *cis*-expression quantitative trait loci (*cis*-eQTLs) of 49 tissues in the GTEx project by downloading version 8 of the per-tissue *cis*-eQTL data from the GTEx portal^71^. Non-coding outlier regions were each annotated for the number of eQTLs contained for each tissue (range, 0–2). Non-coding regions with CLR scores below the fifth centile (*n* = 14,088), were then used as a ‘neutral’ (non-selected) background control dataset. For each tissue, a subset of regions equal to the number of non-coding outlier regions (*n* = 152) was randomly selected and the number of contained eQTLs tabulated. This process was repeated 1,000 times for each tissue to generate a quantitative distribution of eQTL overlaps. Then, for this initial iteration, a *t*-score was calculated for each tissue. To rank the tissues, we then repeated the initial iteration 1,000 times to generate a distribution of *t*-scores for each tissue. The same process was used to generate non-coding outlier region–eQTL distributions for each tissue in each population.

### Rare and novel variation

To minimize the effect of false SNV discovery related to the low depth of coverage and biases that arise from use of different datasets, analyses of rare and novel variation were carried out using only the data with a high depth of coverage (HC-WGS) found in the H3A-Baylor dataset. Comparisons among populations were made between countries rather than regions or ethnic groups. For each variant, we recorded the ancestral allele, its derived allele frequency both at the dataset level and for the seven populations, its predicted effect on biological function following sequence ontology terms, and the predicted effect of the mutation using SNPEff v.4.3^72^ (Supplementary Table 23).

#### Rare variant definition

We defined a rare variant as a SNV with a derived allele frequency of ≤0.01; in most populations this corresponded to a single variant event. Allele counts were orientated as ancestral or derived rather than reference or alternative to avoid biases that arise from the construction of the reference genomes. Owing to differences in sample size across populations, we subsampled 24 individuals for each population and then built the relative site frequency spectrum for each effect category.

#### Novel variants

By definition, novel variants have not been previously discovered and are generally rare and often private; however, given the dearth of data from Africa, such variants may well be common in a population or set of populations. For the purposes of this analysis, we focused on the high-coverage H3A-Baylor dataset and defined a variant as ‘novel’ if the variant was not present in the dbSNP v.150^73^, ExAC^5^ or gnomAD v.2.0.2^74^ databases, or the TryopanoGEN^18^ or SAHGP^17^ datasets or if the variant was not identified in the AGVP^4^ dataset. We also excluded SNVs that were fixed in all populations but for which the derived allele was inferred to be the reference allele, as possible technical artefacts (incorrect ancestral status).

To visualize the contribution of novel variant discovery for each population, we plotted the number of unique novel variants identified per individual within each population, as well as the number of population-specific novel variants for each population. Novel variant discovery was also represented as a cumulative function, in which we sequentially plotted the number of novel variants that were discovered each time a new population was included. To discriminate between rare and common variant discovery, we also plotted the cumulative number of novel variants discovered using common novel variants only (that is, all novel variants except the ones that were observed only once). Correlation between novel variant discovery and ancestry was calculated for the KS ancestry in BOT, RFF ancestry in CAM, east African ancestry in Berom and non-NC ancestry in MAL. Individual-based ancestry proportions were obtained from ADMIXTURE *K* = 6 results and Pearson’s correlations were determined in R.

#### Highly differentiated variants

Highly differentiated variants (HDVs) were identified by first calculating the derived allele frequency for variants for which the ancestral state was known and then assessing pairwise differences in allele frequency between populations. Although a 25% frequency difference threshold has been used to identify HDVs across different continents^1^, we opted for a more-conservative threshold of a 40% difference in the derived allele frequency, consistent with the modest population sample sizes. We visualized the distribution of HDVs by grouping the difference in derived allele frequencies between any two populations into bins representing 0.1 frequency and plotting the number of variants that fell in each bin. We also created a table of the total number of HDVs between each pairwise population comparison and the number of HDVs once we removed variants linked to *HBB* on chromosome 11 and *HLA* on chromosome 6 (two regions with a large number of HDVs related to known selection and strong linkage disequilibrium). To infer the biological relevance of HDVs, the GWAS Catalog^75^ available on 14 October 2019 was downloaded (https://www.ebi.ac.uk/gwas/api/search/downloads/alternative) under hg37 and formatted using Bedtools^76^. We then intersected HDV positions with SNP positions of genome-wide significant (*P* < 5 × 10^−8^), replicated GWAS hits within 50 kb of the HDV. The results were visualized using Circa (http://omgenomics.com/circa/) (Supplementary Table 23). Overlapping GWAS hits and their corresponding experimental trait factors were then further analysed for biological relevance (see below).

#### GWAS Catalog experimental trait factor analysis

We next performed an experimental trait factor analysis using the GWAS Catalog. Genetic variants influence changes in phenotype and physiology in different population groups. As these groups often share similar environment conditions, we expect changes that are essential to adaptation to be conserved, even though this may be constrained by genetic capacity. At a molecular level, this could lead to the divergence (variation) or convergence (conservation) of protein function or structure between groups. Experimental Factor Ontology (EFO) annotations were retrieved from the European Bioinformatics Institute (EBI) website (https://www.ebi.ac.uk/ols/ontologies/efo) and those mapping to GWAS SNVs within 50 kb of HDVs were extracted from https://www.ebi.ac.uk/gwas/docs/file-downloads.

To elucidate potential divergent EFO annotations associated with the identified HDVs, we computed Kappa Statistic similarity scores between EFO annotations based on the population in which the associated SNV was found^77^. These similarity scores were computed on a collapsed EFO at level 4 with the root of the ontology assumed to be located at the level 0, and using only ‘process’, ‘material property’ and ‘material entity’ upper level concepts. Although a similarity score threshold of 0.3 or 0.4 has been often used to identify convergent (>0.3) or divergent (<0.3) annotations, we opted for a stricter threshold of 0.2 based on the similarity score dataset of all EFO annotation pairs. Finally, we mapped different divergent EFO annotations to their associated proteins to identify enriched biological processes and molecular functions that reflect genomic variations among different population groups using the ClueGO software^78^.

#### Putative LOF variation

For the analysis of putative LOF variants, we used a local pipeline—ALOFT^79^—to provide annotations for putative protein-damaging variants in protein-coding genes, including stop-loss, stop-gain and canonical splice sites. This class of variants includes those in the penultimate and last exons of genes (predicted to escape nonsense-mediated decay), but are most similar to variants labelled as putative LOF variants in the literature. These variants were then mapped to their respective genes using BioMart^80^. Downstream analysis was performed using online databases of disease–gene associations, including DisGenet^81^, eDGAR^82^, OMIM^65^ and CTDbase^83^.

For the putative LOF burden analysis, lists of genes that are associated with or that influence influenza, HIV, malaria and hepatitis C infections were extracted using GeneCards^66^ (Supplementary Table 18). These lists are largely populated by genes involved in the host transcriptional response to infection. For each population group, we first determined the number of putative LOF variants in human genes directly implicated in the infection as defined by genecards (direct genes). The majority (75.3%) of putative LOF variants in influenza-associated direct genes were apparent population-singleton variants, with only around 13% being shared between populations (Supplementary Table 18). After extensive benchmarking (Supplementary Methods Figs. 2, 3), we chose to normalize the putative LOF burden in direct genes by dividing by the number of putative LOF variants in ‘indirect’ genes associated with the same infection. This was done to account for potential differences in mutation rate and any uncharacterized gene biases between populations. The putative LOF ratio for each population was then plotted against the mortality rates reported by WHO in 2016^33^ for the infection being surveyed. The correlation between the two values was then calculated, and its significance measured against 1,000 iterations of calculated ratios using a set of random genes similar in data size to the direct gene list for that disease.

### Medically relevant variants

The full dataset (both low- and high-coverage WGS data) was used to catalogue medically relevant variants.

#### Medically actionable variants

SNVs were first annotated against the list of genes included in the ACMG recommendations for reporting of incidental findings in clinical exome- and genome-sequencing data^34,84^. These recommendations are based on the recognition that in whole-exome sequencing or WGS, incidental or secondary findings that are unrelated to the indication for ordering the sequencing but of potential relevance to patient care may be found. We estimated the burden of ACMG gene panel version 2.0 variants, including the total number of alleles observed, median and range of number of alleles per sample, number (%) of samples that carried at least one allele, and genes in which variants were identified.

#### Clinical annotation of variants

SNVs were also annotated for clinical importance using the NCBI ClinVar database (http://www.ncbi.nlm.nih.gov/clinvar/; 20181028 version)^85^. ClinVar provides a freely available report archive of relationships between medically relevant variants and phenotypes. The terms for clinical significance reported by ClinVar are those recommended by the ACMG. These range from ‘0-Uncertain significance’ to ‘5-Pathogenic’ and include codes such as ‘255-Other’. In the present study, variants of interest were those classified by ClinVar as ‘Pathogenic’, which corresponds to ‘5-Pathogenic’ in the ASN.1 set of terms. Frequencies were estimated for the whole dataset and by country. Similar to the analysis of ACMG variants, the burden of these variants was also computed, including the total number of alleles observed, median (range) number of alleles per sample, number (%) of samples that carried at least one allele and genes in which variants were identified.

#### Variants of clinical importance to African populations

We also defined the burden of variants of particular clinical importance to African populations. Population burden and inter-population differentiation were determined for genetic variants related to (1) sickle cell anaemia (*HBB*); (2) trypanosomiasis and end-stage renal disease (*APOL1*); (3) glucose-6-phosphate dehydrogenase deficiency (*G6PD*); and (4) response to antiretroviral therapy with abacavir (HLA-B*5701).

Sickle cell anaemia is an autosomal recessive disorder determined principally by a missense mutation in the *HBB* gene (rs334; HbS). The disorder is most common in Africa, in particular west and central Africa, and remains one of the most-important monogenic disorders of clinical and public health relevance on the continent. Two cohorts from the H3A-Baylor dataset included individuals with homozygous (HbSS) sickle cell disease (CAM and FNB), and these were excluded from frequency estimates of the HbS allele.

Variants in *APOL1* are associated with various forms of kidney disease, primarily in individuals of African ancestry and is protective against severe disease caused by infection with *Trypanosoma brucei*. The two major risk variants (haplotypes) are labelled G1 (defined by rs73885319, often in complete linkage disequilibrium with rs60910145 (T > G)) and G2 (a 6-bp indel, rs143830837). The risk genotype is the recessive state for the diplotype that includes two risk variants: that is, G1/G1, G1/G2, G2/G2.

G6PD deficiency is an X-linked red cell enzymopathy that increases the risk of haemolysis in affected individuals. It is an important risk factor for neonatal jaundice and drug-related haemolysis. Male individuals have the disorder if they carry a *G6PD* mutation on their X chromosome (that is, hemizygous), whereas most affected female individuals carry mutations on both X chromosomes (homozygous). Although there are over 180 known mutations in the gene, including several deleterious mutations, most of these mutations are rare or have a low frequency. The current analysis focused on the common variants definitively associated with *G6PD* deficiency in sub-Saharan Africa, namely, the 202A/376G *G6PD* A allele (that is, the *G6PD* A-deficiency states associated with either rs1050828 (c.202G>A) or rs1050829 (c.376A>G). We also examined a set of 11 *G6PD* variants recently shown to be associated with protection from severe malaria^34^.

The rs2395029 SNP in HLA-B*5701 mediates an adverse allergic response to HIV therapy with abacavir and is found in up to 5% of individuals of European ancestry.

For each of the vignette variants, overall African frequencies and frequencies by country or ethnolinguistic grouping were estimated.

### Reporting summary

Further information on research design is available in the Nature Research Reporting Summary linked to this paper.

## Online content

Any methods, additional references, Nature Research reporting summaries, source data, extended data, supplementary information, acknowledgements, peer review information; details of author contributions and competing interests; and statements of data and code availability are available at 10.1038/s41586-020-2859-7.

## Supplementary information

Supplementary Information This file contains Supplementary Notes 1-5, Supplementary Figures 1-20, Supplementary Methods Figures 1–3 and Supplementary References.

Reporting Summary

Supplementary Tables This file contains Supplementary Methods Tables 1-2 and 23 Supplementary Tables (referred to in the main Supplementary Information file).

## Data Availability

WGS data used in this paper are available through the European Genome-phenome Archive (EGA) under study accession number: EGAS00001002976. The data include genomic (BAMs and VCFs) and minimal phenotypic data from appropriately consented individuals. In compliance with current international standards to protect participant confidentiality, the H3Africa-generated data are available to bona fide researchers within the wider scientific community through a controlled access process. Some of the DNA samples are archived in H3Africa biorepositories as part of the H3Africa Consortium agreement. To gain access to data in the EGA or biospecimens in the biorepositories, requests must be submitted to dbac@h3africa.org, or requested through the H3Africa Data and Biospecimen Catalogue (https://catalogue.h3africa.org). Requests are subject to approval by an independent H3Africa Data and Biospecimen Access Committee (DBAC). Novel SNVs identified and reported here will be deposited into dbSNP. The H3Africa Initiative is committed to providing research data generated by the H3Africa research projects to the entire research community. H3Africa research seeks to promote fair collaboration between scientists in Africa and those from elsewhere. The H3Africa Consortium Data Sharing, Access and Release Policy outlines a policy framework that places a firm focus on African leadership and capacity building as guiding principles for African genomics research. The policy and related documents are available here: https://h3africa.org/index.php/consortium/consortium-documents/.

## Code availability

Code for the implementation of PROCRUSTES is available at https://github.com/dshriner/Procrustes, licensed under the GNU General Public License v.3.0.

## References

[1] Nielsen R (2017). Tracing the peopling of the world through genomics. Nature.

[2] The 1000 Genomes Project Consortium (2015). A global reference for human genetic variation. Nature.

[3] Tishkoff SA (2009). The genetic structure and history of Africans and African Americans. Science.

[4] Gurdasani D (2015). The African Genome Variation Project shapes medical genetics in Africa. Nature.

[5] Lek M (2016). Analysis of protein-coding genetic variation in 60,706 humans. Nature.

[6] Posey JE (2019). Insights into genetics, human biology and disease gleaned from family based genomic studies. Genet. Med..

[7] Landry LG, Ali N, Williams DR, Rehm HL, Bonham VL (2018). Lack of diversity in genomic databases is a barrier to translating precision medicine research into practice. Health Aff..

[8] H3Africa Consortium (2014). Enabling the genomic revolution in Africa. Science.

[9] Patin E (2017). Dispersals and genetic adaptation of Bantu-speaking populations in Africa and North America. Science.

[10] Hanchard N (2007). Classical sickle beta-globin haplotypes exhibit a high degree of long-range haplotype similarity in African and Afro-Caribbean populations. BMC Genet..

[11] Ranciaro A (2014). Genetic origins of lactase persistence and the spread of pastoralism in Africa. Am. J. Hum. Genet..

[12] Genovese G (2010). Association of trypanolytic ApoL1 variants with kidney disease in African Americans. Science.

[13] Sabeti PC (2002). Detecting recent positive selection in the human genome from haplotype structure. Nature.

[14] Schlebusch CM (2012). Genomic variation in seven Khoe-San groups reveals adaptation and complex African history. Science.

[15] Scheinfeldt LB (2019). Genomic evidence for shared common ancestry of East African hunting-gathering populations and insights into local adaptation. Proc. Natl Acad. Sci. USA.

[16] Skoglund P (2017). Reconstructing prehistoric African population structure. Cell.

[17] Choudhury A (2017). Whole-genome sequencing for an enhanced understanding of genetic variation among South Africans. Nat. Commun..

[18] Ilboudo H (2017). Introducing the TrypanoGEN biobank: a valuable resource for the elimination of human African trypanosomiasis. PLoS Negl. Trop. Dis..

[19] Alexander DH, Novembre J, Lange K (2009). Fast model-based estimation of ancestry in unrelated individuals. Genome Res..

[20] Patterson N (2012). Ancient admixture in human history. Genetics.

[21] Semo A (2020). Along the Indian Ocean coast: genomic variation in Mozambique provides new insights into the Bantu expansion. Mol. Biol. Evol..

[22] Loh P-R (2013). Inferring admixture histories of human populations using linkage disequilibrium. Genetics.

[23] Patin E (2014). The impact of agricultural emergence on the genetic history of African rainforest hunter-gatherers and agriculturalists. Nat. Commun..

[24] Shriner D, Rotimi CN (2018). Genetic history of Chad. Am. J. Phys. Anthropol..

[25] Campbell IM (2016). Multiallelic positions in the human genome: challenges for genetic analyses. Hum. Mutat..

[26] Campbell MC, Tishkoff SA (2008). African genetic diversity: implications for human demographic history, modern human origins, and complex disease mapping. Annu. Rev. Genomics Hum. Genet..

[27] Pavlidis P, Živkovic D, Stamatakis A, Alachiotis N (2013). SweeD: likelihood-based detection of selective sweeps in thousands of genomes. Mol. Biol. Evol..

[28] Szpiech ZA, Hernandez RD (2014). selscan: an efficient multithreaded program to perform EHH-based scans for positive selection. Mol. Biol. Evol..

[29] Vitti JJ, Grossman SR, Sabeti PC (2013). Detecting natural selection in genomic data. Annu. Rev. Genet..

[30] Yi X (2010). Sequencing of 50 human exomes reveals adaptation to high altitude. Science.

[31] Retshabile G (2018). Whole-exome sequencing reveals uncaptured variation and distinct ancestry in the southern African population of Botswana. Am. J. Hum. Genet..

[32] Lim ET (2014). Distribution and medical impact of loss-of-function variants in the Finnish founder population. PLoS Genet..

[33] World Health Organization. *WHO Influenza (Seasonal): Fact Sheet* https://www.who.int/news-room/fact-sheets/detail/influenza-(seasonal) (2016).

[34] Kalia SS (2017). Recommendations for reporting of secondary findings in clinical exome and genome sequencing, 2016 update (ACMG SF v2.0): a policy statement of the American College of Medical Genetics and Genomics. Genet. Med..

[35] Manjurano A (2015). African glucose-6-phosphate dehydrogenase alleles associated with protection from severe malaria in heterozygous females in Tanzania. PLoS Genet..

[36] Howes RE, Battle KE, Satyagraha AW, Baird JK, Hay SI (2013). G6PD deficiency: global distribution, genetic variants and primaquine therapy. Adv. Parasitol..

[37] Kimuda MP (2018). No evidence for association between APOL1 kidney disease risk alleles and human African trypanosomiasis in two Ugandan populations. PLoS Negl. Trop. Dis..

[38] Rotimi CN, Jorde LB (2010). Ancestry and disease in the age of genomic medicine. N. Engl. J. Med..

[39] Phillipson DW (1974). Iron Age history and archaeology in Zambia. J. Afr. Hist..

[40] Schlebusch CM, Jakobsson M (2018). Tales of human migration, admixture, and selection in Africa. Annu. Rev. Genomics Hum. Genet..

[41] Mulindwa J (2020). High levels of genetic diversity within Nilo-Saharan populations: implications for human adaptation. Am. J. Hum. Genet..

[42] Shiroya OJE (1981). The Lugbara states — politics, economics and warfare in the eighteenth and nineteenth centuries. TransAfrican J. Hist..

[43] R Core Team. R: A Language and Environment for Statistical Computing. http://www.R-project.org/ (R Foundation for Statistical Computing, 2017).

[44] Li H, Durbin R (2009). Fast and accurate short read alignment with Burrows–Wheeler transform. Bioinformatics.

[45] McKenna A (2010). The Genome Analysis Toolkit: a MapReduce framework for analyzing next-generation DNA sequencing data. Genome Res..

[46] Li H (2009). The Sequence Alignment/Map format and SAMtools. Bioinformatics.

[47] O’Connell J (2014). A general approach for haplotype phasing across the full spectrum of relatedness. PLoS Genet..

[48] Loh PR, Palamara PF, Price AL (2016). Fast and accurate long-range phasing in a UK Biobank cohort. Nat. Genet..

[49] Chang CC (2015). Second-generation PLINK: rising to the challenge of larger and richer datasets. Gigascience.

[50] Patterson N, Price AL, Reich D (2006). Population structure and eigenanalysis. PLoS Genet..

[51] Buchmann, R. & Hazelhurst, S. *Genesis PCA and Admixture Plot Viewer*. Version 0.2.6 http://www.bioinf.wits.ac.za/software/genesis (2014).

[52] Jakobsson M, Rosenberg NA (2007). CLUMPP: a cluster matching and permutation program for dealing with label switching and multimodality in analysis of population structure. Bioinformatics.

[53] Wang C (2010). Comparing spatial maps of human population-genetic variation using Procrustes analysis. Stat. Appl. Genet. Mol. Biol..

[54] Pickrell JK, Pritchard JK (2012). Inference of population splits and mixtures from genome-wide allele frequency data. PLoS Genet..

[55] Pickrell JK (2014). Ancient west Eurasian ancestry in southern and eastern Africa. Proc. Natl Acad. Sci. USA.

[56] Browning BL, Browning SR (2013). Improving the accuracy and efficiency of identity-by-descent detection in population data. Genetics.

[57] Atzmon G (2010). Abraham’s children in the genome era: major Jewish diaspora populations comprise distinct genetic clusters with shared Middle Eastern ancestry. Am. J. Hum. Genet..

[58] Maples BK, Gravel S, Kenny EE, Bustamante CD (2013). RFMix: a discriminative modeling approach for rapid and robust local-ancestry inference. Am. J. Hum. Genet..

[59] Haber M (2016). Chad genetic diversity reveals an African history marked by multiple Holocene Eurasian migrations. Am. J. Hum. Genet..

[60] Weissensteiner H (2016). HaploGrep 2: mitochondrial haplogroup classification in the era of high-throughput sequencing. Nucleic Acids Res..

[61] Van Geystelen A, Decorte R, Larmuseau MHD (2013). AMY-tree: an algorithm to use whole genome SNP calling for Y chromosomal phylogenetic applications. BMC Genomics.

[62] Pemberton TJ (2012). Genomic patterns of homozygosity in worldwide human populations. Am. J. Hum. Genet..

[63] Fumagalli M (2013). Assessing the effect of sequencing depth and sample size in population genetics inferences. PLoS ONE.

[64] Zerbino DR (2018). Ensembl 2018. Nucleic Acids Res..

[65] Amberger JS, Bocchini CA, Scott AF, Hamosh A (2019). OMIM.org: leveraging knowledge across phenotype-gene relationships. Nucleic Acids Res..

[66] Stelzer G (2016). The GeneCards suite: from gene data mining to disease genome sequence analyses. Curr. Protoc. Bioinformatics.

[67] Pybus M (2014). 1000 Genomes Selection Browser 1.0: a genome browser dedicated to signatures of natural selection in modern humans. Nucleic Acids Res..

[68] Sabeti PC (2007). Genome-wide detection and characterization of positive selection in human populations. Nature.

[69] Pickrell JK (2009). Signals of recent positive selection in a worldwide sample of human populations. Genome Res..

[70] Danecek P (2011). The variant call format and VCFtools. Bioinformatics.

[71] GTEx Consortium (2013). The Genotype-Tissue Expression (GTEx) project. Nat. Genet..

[72] Cingolani P (2012). Using *Drosophila melanogaster* as a model for genotoxic chemical mutational studies with a new program, SnpSift. Front. Genet..

[73] Sherry ST (2001). dbSNP: the NCBI database of genetic variation. Nucleic Acids Res..

[74] Karczewski KJ (2020). The mutational constraint spectrum quantified from variation in 141,456 humans. Nature.

[75] MacArthur J (2017). The new NHGRI-EBI Catalog of published genome-wide association studies (GWAS Catalog). Nucleic Acids Res..

[76] Quinlan AR, Hall IM (2010). BEDTools: a flexible suite of utilities for comparing genomic features. Bioinformatics.

[77] Mazandu GK, Chimusa ER, Mbiyavanga M, Mulder NJ (2016). A-DaGO-Fun: an adaptable Gene Ontology semantic similarity-based functional analysis tool. Bioinformatics.

[78] Bindea G (2009). ClueGO: a Cytoscape plug-in to decipher functionally grouped gene ontology and pathway annotation networks. Bioinformatics.

[79] Balasubramanian S (2017). Using ALoFT to determine the impact of putative loss-of-function variants in protein-coding genes. Nat. Commun..

[80] Smedley D (2015). The BioMart community portal: an innovative alternative to large, centralized data repositories. Nucleic Acids Res..

[81] Piñero J (2017). DisGeNET: a comprehensive platform integrating information on human disease-associated genes and variants. Nucleic Acids Res..

[82] Babbi G (2017). eDGAR: a database of disease–gene associations with annotated relationships among genes. BMC Genomics.

[83] Davis AP (2019). The Comparative Toxicogenomics Database: update 2019. Nucleic Acids Res..

[84] ACMG Board of Directors (2015). ACMG policy statement: updated recommendations regarding analysis and reporting of secondary findings in clinical genome-scale sequencing. Genet. Med..

[85] Landrum MJ (2018). ClinVar: improving access to variant interpretations and supporting evidence. Nucleic Acids Res..

