# Author Correction: High-depth African genomes inform human migration and health

**DOI:** 10.1038/s41586-021-03286-9

**Published:** 2021-04-12

**Authors:** Ananyo Choudhury, Shaun Aron, Laura R. Botigué, Dhriti Sengupta, Gerrit Botha, Taoufik Bensellak, Gordon Wells, Judit Kumuthini, Daniel Shriner, Yasmina J. Fakim, Anisah W. Ghoorah, Eileen Dareng, Trust Odia, Oluwadamilare Falola, Ezekiel Adebiyi, Scott Hazelhurst, Gaston Mazandu, Oscar A. Nyangiri, Mamana Mbiyavanga, Alia Benkahla, Samar K. Kassim, Nicola Mulder, Sally N. Adebamowo, Emile R. Chimusa, Donna Muzny, Ginger Metcalf, Richard A. Gibbs, TrypanoGEN Research Group, Enock Matovu, Bruno Bucheton, Christiane Hertz-Fowler, Mathurin Koffi, Annette Macleod, Dieudonne Mumba-Ngoyi, Harry Noyes, Oscar A. Nyangiri, Gustave Simo, Martin Simuunza, Charles Rotimi, Michèle Ramsay, H3Africa Consortium, Ananyo Choudhury, Shaun Aron, Laura Botigué, Dhriti Sengupta, Gerrit Botha, Taoufik Bensellak, Gordon Wells, Judit Kumuthini, Daniel Shriner, Yasmina J. Fakim, Anisah W. Ghoorah, Eileen Dareng, Trust Odia, Oluwadamilare Falola, Ezekiel Adebiyi, Scott Hazelhurst, Gaston Mazandu, Oscar A. Nyangiri, Mamana Mbiyavanga, Alia Benkahla, Samar K. Kassim, Nicola Mulder, Sally N. Adebamowo, Emile R. Chimusa, Charles Rotimi, Michèle Ramsay, Adebowale A. Adeyemo, Zané Lombard, Neil A. Hanchard, Clement Adebamowo, Godfred Agongo, Romuald P. Boua, Abraham Oduro, Hermann Sorgho, Guida Landouré, Lassana Cissé, Salimata Diarra, Oumar Samassékou, Gabriel Anabwani, Mogomotsi Matshaba, Moses Joloba, Adeodata Kekitiinwa, Graeme Mardon, Sununguko W. Mpoloka, Samuel Kyobe, Busisiwe Mlotshwa, Savannah Mwesigwa, Gaone Retshabile, Lesedi Williams, Ambroise Wonkam, Ahmed Moussa, Dwomoa Adu, Akinlolu Ojo, David Burke, Babatunde O. Salako, Enock Matovu, Bruno Bucheton, Christiane Hertz-Fowler, Mathurin Koffi, Annette Macleod, Dieudonne Mumba-Ngoyi, Harry Noyes, Oscar A. Nyangiri, Gustave Simo, Martin Simuunza, Philip Awadalla, Vanessa Bruat, Elias Gbeha, Adebowale A. Adeyemo, Zané Lombard, Neil A. Hanchard

**Affiliations:** 1 Sydney Brenner Institute for Molecular Bioscience, Faculty of Health Sciences, University of the Witwatersrand, Johannesburg, South Africa; 2 Center for Research in Agricultural Genomics (CRAG), Plant and Animal Genomics Program, CSIC-IRTA-UAB-UB, Barcelona, Spain; 3 Computational Biology Division and H3ABioNet, Department of Integrative Biomedical Sciences, IDM, University of Cape Town, Cape Town, South Africa; 4 System and Data Engineering Team, Abdelmalek Essaadi University, ENSA, Tangier, Morocco; 5 Centre for Proteomic and Genomic Research (CPGR), Cape Town, South Africa; 6 South African National Bioinformatics Institute, University of the Western Cape, Bellville, South Africa; 7 Center for Research on Genomics and Global Health, National Human Genome Research Institute, National Institutes of Health, Bethesda, MD USA; 8 Department of Agriculture and Food Science, Faculty of Agriculture, University of Mauritius, Reduit, Mauritius; 9 Department of Digital Technologies,Faculty of Information, Communication & Digital Technologies, University of Mauritius, Reduit, Mauritius; 10 Department of Public Health and Primary Care, University of Cambridge, Cambridge, UK; 11 Institute of Human Virology Nigeria, Abuja, Nigeria; 12 Covenant University Bioinformatics Research (CUBRe), Covenant University, Ota, Nigeria; 13 Department of Computer and Information Sciences, Covenant University, Ota, Nigeria; 14 School of Electrical and Information Engineering, University of the Witwatersrand, Johannesburg, South Africa; 15 College of Veterinary Medicine, Animal Resources and Biosecurity, Makerere University, Kampala, Uganda; 16 Laboratory of Bioinformatics, Biomathematics and Biostatistics (BIMS), Institute Pasteur of Tunis, Tunis, Tunisia; 17 Medical Biochemistry and Molecular Biology Department, Faculty of Medicine, Ain Shams University, Abbaseya, Cairo, Egypt; 18 Department of Epidemiology and Public Health, University of Maryland School of Medicine, University of Maryland Baltimore, Baltimore, MD USA; 19 University of Maryland Greenebaum Comprehensive Cancer Center, University of Maryland School of Medicine, University of Maryland Baltimore, Baltimore, MD USA; 20 Division of Human Genetics, Department of Pathology, Faculty of Health Sciences, Institute for Infectious, Disease and Molecular Medicine, University of Cape Town, Cape Town, South Africa; 21 Human Genome Sequencing Center, Baylor College of Medicine, Houston, TX USA; 22 Department of Molecular and Human Genetics, Baylor College of Medicine, Houston, Texas USA; 23 Division of Human Genetics, National Health Laboratory Service, and School of Pathology, Faculty of Health Sciences, University of the Witwatersrand, Johannesburg, South Africa; 49 Africa Health Research Institute, Durban, South Africa; 39 Institut de Recherche pour le Développement, Montpellier, France; 40 Institute of Integrative Biology, University of Liverpool, Liverpool, UK; 41 Wellcome Trust, London, UK; 42 Jean Lorougnon Guede University, Daloa, Côte d’Ivoire; 43 Institute of Biodiversity Animal Health and Comparative Medicine, University of Glasgow, Glasgow, UK; 44 Institut National de Recherche Biomedicale, Kinshasa, Democratic Republic of Congo; 45 Faculty of Science, University of Dschang, Dschang, Cameroon; 46 School of Veterinary Medicine, University of Zambia, Lusaka, Zambia; 24 Institute of Human Virology and Greenebaum Cancer Center, University of Maryland School of Medicine, Baltimore, MD USA; 25 Navrongo Health Research Centre, Navrongo, Ghana; 26 Clinical Research Unit of Nanoro, Institut de Recherche en Sciences de la Sante, Bobo-Dioulasso, Burkina Faso; 27 Faculty of Medicine and Odontostomatology, University of Science, Techniques and Technologies of Bamako (USTTB), Bamako, Mali; 28 Service de Neurologie, Centre Hospitalier Universitaire du Point “G”, Bamako, Mali; 29 Neurogenetics Branch, National Institute of Neurological Disorders and Stroke, National Institutes of Health, Bethesda, MD USA; 30 Botswana-Baylor Children’s Clinical Centre of Excellence, Gaborone, Botswana; 31 Medical Microbiology, College of Health Sciences, Makerere University, Kampala, Uganda; 32 Baylor College of Medicine Children’s Foundation, Kampala, Uganda; 33 Department of Pathology and Immunology, Baylor College of Medicine, Houston, TX USA; 34 Department of Biological Sciences, University of Botswana, Gaborone, Botswana; 35 University of Ghana Medical School, Accra, Ghana; 36 The University of Kansas School of Medicine, Kansas City, KS USA; 37 Department of Human Genetics, University of Michigan Medical School, Ann Arbor, MI USA; 38 College of Medicine, University of Ibadan, Ibadan, Nigeria; 47 Department of Molecular Genetics, University of Toronto, Toronto, Ontario Canada; 48 Ontario Institute for Cancer Research, Toronto, Ontario Canada

**Keywords:** Evolutionary genetics, Genetic variation, Next-generation sequencing, Genetics research

Correction to: *Nature* 10.1038/s41586-020-2859-7Published online 28 October 2020

In this Article, the affiliation ‘South African National Bioinformatics Network, University of the Western Cape, Bellville, South Africa’, which is associated with authors Gordon Wells and Judit Kumuthini, should be ‘South African National Bioinformatics **Institute**, University of the Western Cape, Bellville, South Africa’. In addition, author Gordon Wells should be associated with an additional affiliation, ‘Africa Health Research Institute, Durban, South Africa’. In Fig. 1a, the circle representing the data for South Africa should be dark grey (indicating the AGVP data source) rather than light grey (indicating the 1000G data source). The Article has been corrected online.

