# Supplementary material for: High-depth African genomes inform human migration and health
Source: Nature. 2020 Oct 28;586(7831):741–8. doi: 10.1038/s41586-020-2859-7 (PMC7759466; doi:10.1038/s41586-020-2859-7)
Supplement: Supplementary file 1 — This file contains Supplementary Notes 1-5, Supplementary Figures 1-20, Supplementary Methods Figures 1–3 and Supplementary References. [file 41586_2020_2859_MOESM1_ESM.pdf]

natureresearch

https://doi.org/10.1038/s41586-020-2859-7

Supplementary information

High-depth African genomes inform human migration and health

In the format provided by the authors and unedited

SUPPLEMENTARY INFORMATION

High Depth African Genomes Inform Human Migration and Health

Ananyo Choudhury[1], Shaun Aron[1], Laura Botigué[2], Dhriti Sengupta[1], Gerrit Botha[3], Taoufik Bensellak[4], Gordon Wells[5,6], Judit Kumuthini[6], Daniel Shriner[7], Yasmina J Fakim[8,9], Anisah W Ghoorah[9], Eileen Dareng[10,11], Trust Odia[12], Oluwadamilare Falola[12], Ezekiel Adebiyi[12,13], Scott Hazelhurst [1,14], Gaston Mazandu[3], Oscar A. Nyangiri[15], Mamana Mbiyavanga[3], Alia Benkahla[16], Samar K. Kassim[17], Nicola Mulder[3], Sally N. Adebamowo[18], Emile R. Chimusa[19], Donna Muzny[20], Ginger Metcalf[20], Richard A Gibbs[20,21], TrypanoGEN Research Group[†], Charles Rotimi[7], Michèle Ramsay[1,22], H3Africa Consortium[‡], Adebowale Adeyemo[7]*, Zané Lombard[22]*, Neil A. Hanchard[21]*

SUPPLEMENTARY NOTES 3
Supplementary Note 1 - Population structure and gene flow 3
1.1 - Language classification 3
1.2 - Gene flow in Ugandan Nilo Saharan (UNS) and Berom from Nigeria (BRN) 3
1.3 - Admixture masking 4
1.4 - Admixture graph analysis 4
1.5 - Admixture dating in the Berom (BRN) 7
1.6 - Populations consisting of multiple ethnolinguistic groups 7
1.7 - Analysis of runs of homozygosity (ROH) 8
1.8 - mtDNA and Y chromosome haplogroup analysis 8
Supplementary Note 2 - Signatures of selection 8
2.1 CLR Signals for previously characterized genes 8
2.2 CLR outlier windows in non-coding regions 9
2.3 Population branch statistics (PBS) analysis 9
Supplementary Note 3 – Site-frequency-spectra and putative loss-of-function variants 11
Supplementary Note 4 – ClinVar pathogenic variants in the H3Africa dataset 11
Supplementary Note 5 - Ethics statements from participating studies 12

SUPPLEMENTARY FIGURES 14
Supplementary Figure 1 – Extended principal components (PCs) of H3Africa populations from WGS data. 14
Supplementary Figure 2 – PC based evaluation of novel WGS extended to include data from Schlebusch et al.[12] and Patin et al.[15] 15
Supplementary Figure 3 - Correlation between Genetic and Geographic Distances using PROCRUSTES. 17

Supplementary Figure 4 - Cross validation error scores for ADMIXTURE clustering. 18
Supplementary Figure 5 - PC and ADMIXTURE clustering analysis of Ugandan Nilo- Saharan (UNS)..19
Supplementary Figure 6 - Genetic affinities and ancestral composition of Berom (BRN). 20
Supplementary Figure 7 - Admixture masking based assessment of non-NC speaker gene flow..............21
Supplementary Figure 8 - Estimates of genetic affinities of the Zambian (BSZ) population.....................22
Supplementary Figure 9 - Principal component and ADMIXTURE clustering analysis of ethnolinguistic groups...23
Supplementary Figure 10 - Distribution of runs of homozygosity (ROH) segments in representative African populations. 24
Supplementary Figure 11 - Distribution of total lengths of (A), short (<500kb) (B), medium (500kb to 1.5MB) and (C) long (>1.5MB) ROH in MAL individuals...25
Supplementary Figure 12 - Distribution of mitochondrial and Y chromosome haplogroups in H3A-high coverage WGS samples. 26
Supplementary Figure 13 - Variant sharing between populations in the H3A high coverage WGS dataset. 27
Supplementary Figure 14 - Map of significant GWAS hits found in non-coding regions with outlier CLR selections scores. 28
Supplementary Figure 15 - Map of significant GTEx eQTL overlapping non-coding regions with outlier CLR scores. 29
Supplementary Figure 16 - Significant GTEx eQTL overlapping non-coding regions with outlier CLR scores by tissue..30
Supplementary Figure 17 - Significant GTEx eQTL overlapping non-coding regions with outlier CLR scores by tissue by population. 31
Supplementary Figure 18 – Site frequency spectra among high coverage WGS H3Africa populations by variant class. 32
Supplementary Figure 19 - Heatmap of frequencies of common pLoF variants shared across H3Africa populations in *metabolic disease* genes 34
Supplementary Figure 20 – ClinVar Pathogenic (level 5) and likely-pathogenic (level 4) variants in 1000 Genomes African populations...35

SUPPLEMENTARY METHODS FIGURES 36
Supplementary Methods Figure 1 - Overview of joint calling process resulting in merged VCF file. 36
Supplementary Methods Figure 2 - Comparison of Influenza "direct" and "indirect" gene characteristics. 37
Supplementary Methods Figure 3 - Benchmarking of pLOF burden ratio. 38
REFERENCES...39

SUPPLEMENTARY NOTES

Supplementary Note 1 - Population structure and gene flow

1.1 - Language classification

Over the last few thousand years, the Khoe and San peoples lived over a large area of southern and eastern Africa. The term Khoesan is widely used both as descriptor of language and people. However, over the last 20 years many linguists now find the relationship between the Khoe and San languages tenuous[1]. Some San people object to the term[2]. Although it is likely there was extended interaction between the Khoe and San people, and since colonial times there has been much more. Since the Khoe and San have distinct histories, we prefer the term Khoe and San over Khoesan. We have used the standard convention of naming Khoe and San from Southern Africa as KS and forager populations from Central African rain forests as RFF. The name and spellings of other ethnolinguistic groups are based on information supplied by data providers. We recognize that this nomenclature might have limitations and may not always reflect the manner in which some of these groups prefer to identify themselves. We apologize in advance for any discomfort or distress that might have been caused due to this. Moreover, our insights into population affinities and demographic histories are based on limited number of samples, collected from a single or a few sites. These observations therefore might not represent the ethnolinguistic groups or geographies comprehensively and any social or political extrapolation of these results should be avoided.

1.2 - Gene flow in Ugandan Nilo Saharan (UNS) and Berom from Nigeria (BRN)

To better characterize the RFF and other gene flow in UNS we conducted PC and ADMIXTURE analysis using SNP-array data from Sudanese east African populations[3]. The resulting PCA showed a relative isolation of UNS, not only in comparison to near-by Nilo-Saharan-speakers from Kenya (Masai, Kalenjin) and Ethiopia (Gumuz), but also to Nilo-Saharan-speaker groups such as Nuba from Sudan (**Supplementary Figure 5**). The ADMIXTURE analysis further intimated that the distinction between UNS and other Nilo-Saharan-speaker populations might be the result of an increased gene flow from RFF. The near absence of the Afro-Asiatic admixture that is characteristic of Nilo-Saharan-speakers from Kenya and Ethiopia, but typically not seen among Nilo-Saharan Sudanese speakers, might also have contributed to the differentiation of these populations (**Supplementary Figure 5**).

Using a similar approach based on additional data we also assessed the potential for Nilo-Saharan gene flow into the BRN[4-8]. We identified the Tubu from Chad as the most representative Nilo-Saharan contributor to this unexpected East African component of West African lineage (**Supplementary Figure 6, Supplementary Table 2**). Traces of East African ancestry originating from waves of trans-Sahelian

migrations in the last few thousand years[6,8,9] have been reported in other populations from across Nigeria, Central-African Republic, and Chad (e.g. Hausa, Fulani, Kanuri, Mada, Tubu and Sara); however, this is the first report of East-African gene flow in a large, autochthonous, central Nigerian population.

1.3 - Admixture masking

We assessed the contribution of the gene flow from non-Niger-Congo (NC) groups in differentiating between population groups by masking genomic regions without evidence of Niger-Congo ancestry in the RFMIX analyses[10] and repeating the PC analyses, similar to[11]. Masking of Khoe and San local ancestry based on data from[12]; (**Supplementary Figure 7A and 7B)** resulted in a reduced genetic distance between Batswana (BOT) and Zambian Bantu-speakers (BSZ). This also led to closer clustering of BOT on principal components analysis, suggesting that differential Khoe and San gene flow contributed to both inter- and intra-population differences in this group. Similarly, the masking of East African ancestry based on proxy data from Chad[8,13] reduced the genetic distance between BRN and other West-African populations in the PCA plots (**Supplementary Figure 7A and 7C**).

1.4 - Admixture graph analysis

To test the hypothesis of the migration of Bantu-speakers via a route that passes from Central-West Africa to Angola and Zambia we used the admixture graph technique qpGraph implemented in ADMIXTOOLS[14] to build several graphs to test the fit of the data to four main scenarios (**Supplementary Note Figure 1**): one in which BSZ is one of the sources of admixture for BOT, another in which DRC is the source of admixture, a third one in which the source of admixture is ANG following Patin et al.[15] and a fourth one in which BSZ is the source of admixture of BOT and UBS, representing the Bantu expansion to the South and East, respectively.

For the first scenario, the best model (A), with no outliers and with no poorly constructed branches, consisted of an admixture graph in which an ancestral African population splits into a South African population and another African population, af1, that in turn splits into an Eastern African and a Western African ancestral population. The Eastern African population splits into a central- and eastern African ancestral population. This Eastern African population admixed with the Western African population to give rise to another population that is one of the main donors of BSZ (96%) and CAM (44%). BOT is the product of an admixture of BSZ (70%) and the Southern African population (30%)

We next aimed to introduce DRC into the graph. Under this scenario the best fit of the data (B) was a model in which BSZ is still one of the sources of admixture of BOT (69%). Though it differentiates from the previous scenario in that af1 is the direct ancestor of CAM which is one of the donors of an intermediate

admixed population, af3, which is in turn the donor of BSZ and the direct ancestor of DRC. This model had 10 outliers. The same model with DRC being one of the sources of admixture of BOT supposes a much poorer fit to the data (C), resulting in 31 outliers and a poor fit of the CAM branch. Models in which CAM is sister clade to DRC and or BSZ also resulted in a poor fit with the data.

In the third scenario we tested the fitness of ANG being the donor of this Bantu component to BOT instead of BSZ. In this scenario we removed DRC and incorporated ANG. The best fit to the data (D) for this scenario was a cascade model, in which CAM is a donor of ANG which is in turn a donor of BSZ which is a donor of BOT, reflecting a putative Bantu expansion to the south. This model resulted in 7 outliers only, whereas a model in which ANG is the direct donor of BOT resulted in 9 outliers.

Finally, we wanted to represent a whole demographic model of the Bantu expansion to the south and east, and we therefore started from the best model from the second scenario (B and C) and included Ugandan Bantu Speakers (UBS) to the model. In agreement with our hypothesis, the best fit to the data for this scenario (E) was a model in which both UBS and BOT receive the Bantu component from BSZ, amounting to 58% for the first and 68% for the latter. BSZ is in turn the product of a complex admixture scenario involving an ancestral central African population (caf1) with 65% of their genome being sourced to CAM, and another unmixed central African population (caf2). Interestingly, the admixed caf1 would be a direct ancestor of DRC. It must be noted, though, that this model is far from a perfect fit to the data, as it results in 37 outliers, probably because the relationship between CAM and BSZ has not been properly modelled. Alternative models in which CAM is the direct donor of BSZ (F), an exclusive unadmixed central Africa population (G), and CAM being the direct donor to UBS, all have a poorer fit to the data.

1.4.1 Supplementary Note Figure 1 - Admixture graphs. Models using qpGraph to test the hypothesis that Zambia was an intermediate destination in the route of the Bantu migration from Central - West Africa to the East and South. Branches are indicated by solid black lines (adjacent numbers indicate estimated drift values in units of *f2* instances, parts per thousand), whereas admixture is indicated by coloured dashed lines (adjacent numbers indicate ancestry proportions). Sampled populations are indicated by solid circles with bold outline. Color codes in panel **A** reflect the main ancestries hypothesized in these models: in green a southern African ancestry, in blue an eastern African ancestry, in turquoise a non-Bantu western African ancestry, and in orange a Bantu west African ancestry. The following populations were used to construct the models: BOT n=48 biologically independent samples, CAM n=50 biologically independent samples, ANG n=28 biologically independent samples, DRC n=12 biologically independent samples and BSZ n=29 biologically independent samples.

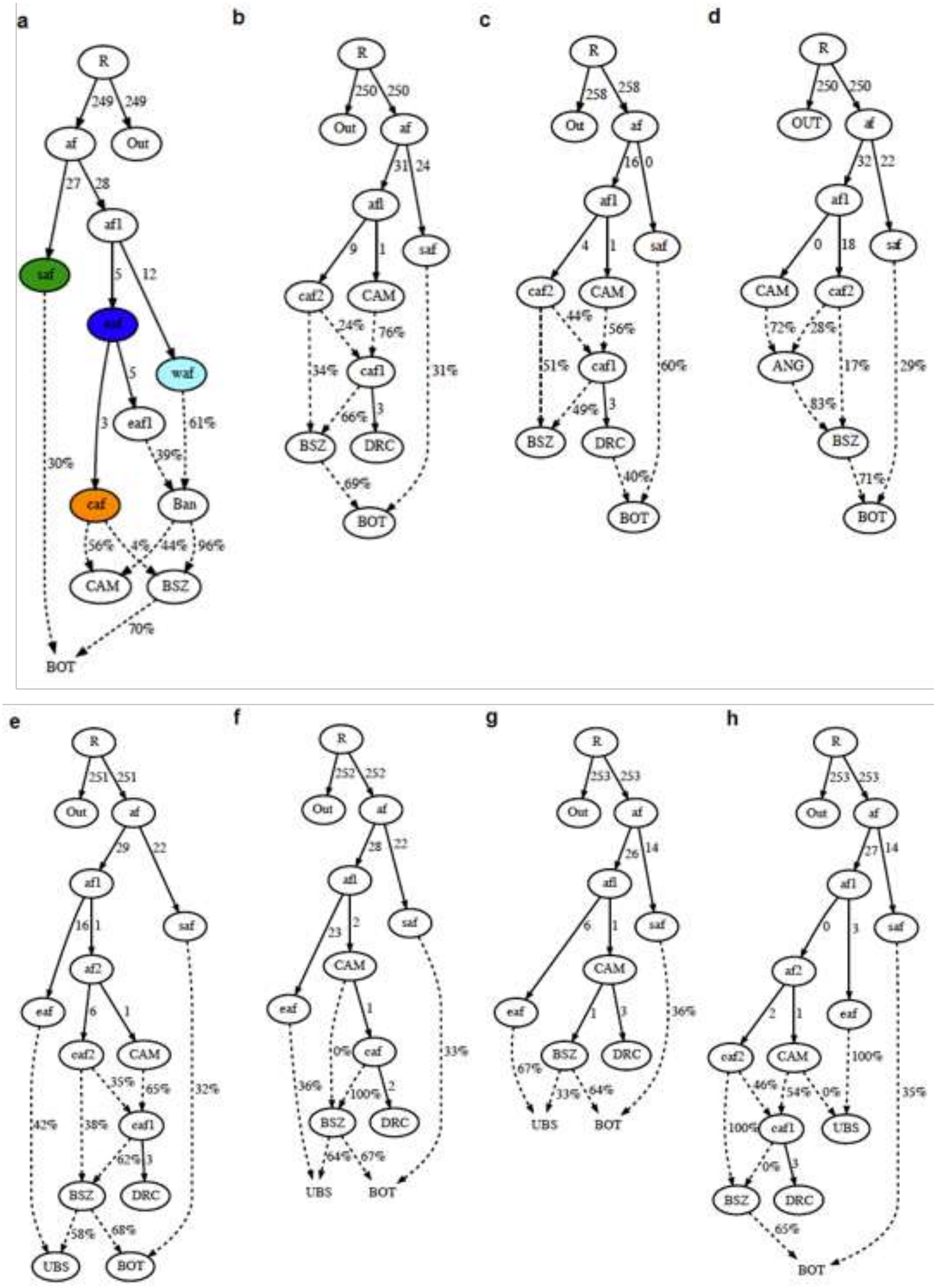
a
R
249
249
af
Out
27
28
afl
saf
5
12
eaf
waf
5
3
eafl
61%
30%
39%
caf
Ban
56%
4%
44%
96%
CAM
BSZ
70%
BOT
b
R
250
250
Out
af
31
24
afl
9
1
saf
caf2
CAM
24%
76%
34%
cafl
31%
66%
3
BSZ
DRC
69%
BOT
c
R
258
258
Out
af
16
0
afl
4
1
saf
caf2
CAM
44%
56%
51%
cafl
60%
49%
3
BSZ
DRC
40%
BOT
d
R
250
250
OUT
af
32
22
afl
0
18
saf
CAM
caf2
72%
28%
ANG
17%
29%
83%
BSZ
71%
BOT
e
R
251
251
Out
af
29
22
afl
16
1
saf
af2
eaf
6
1
caf2
CAM
35%
65%
32%
42%
38%
cafl
62%
3
BSZ
DRC
58%
68%
UBS
BOT
f
R
252
252
Out
af
28
22
afl
23
2
saf
CAM
eaf
1
0%
caf
33%
36%
100%
2
BSZ
DRC
64%
67%
UBS
BOT
g
R
253
253
Out
af
26
14
afl
6
1
saf
CAM
eaf
1
3
36%
BSZ
DRC
67%
33%
64%
UBS
BOT
h
R
253
253
Out
af
27
14
afl
0
3
saf
af2
eaf
2
1
caf2
CAM
100%
46%
54%
0%
35%
100%
cafl
UBS
0%
3
BSZ
DRC
65%
BOT

1.5 - Admixture dating in the Berom (BRN)

Historical and archaeological data suggest that habitation of the Jos-plateau, the homeland of the BRN, occurred in four distinct phases, with the first starting around 200 BC[16]; thus, our analyses implicate east African gene flow to the BRN during the first phase of habitation. This date varies from those reported for other East African gene flow by almost 1000 years, suggesting that the admixture in BRN relates to a distinct historical event. Interestingly, our dates (200-500 CE) overlap two important demographic changes in the region: first, the disintegration of the Nok culture occurred around the time of the first phase of settlement of the Jos Plateau[16,17]. The underlying reasons for this disintegration remain unclear, but climate change has been intimated[17]. A second, alternative hypothesis links the BRN to the Aturu complex, which existed in the region between 100 CE and 1,000 CE[18]. A relative dearth of information on the ethnolinguistic identity of the early settlers of the Jos or the Nok culture, makes a clear interpretation of these dates challenging, but these observations, along with oral history of the Berom referencing a large-scale migration to, and settlement in, the region, highlight an unrecognized and important, early migration pattern for additional study.

1.6 - Populations consisting of multiple ethnolinguistic groups

Four of the groups included in our study (BOT, BSZ, CAM, and MAL) were amalgams of multiple ethnolinguistic groups (**Supplementary Table 3);** therefore, we also assessed the variation in PC projection and ancestry proportions at the level of ethnolinguistic groups in each of these populations (**Supplementary Figure 9**). In Zambia (BSZ) and Cameroon (CAM), individuals from different ethnolinguistic groups showed considerable overlap in PC localization; however, ADMIXTURE analysis suggested subtle differences in ancestry proportions among the CAM groups, with the Ngoumba samples (the most Southern of the three populations) showing slightly higher RFF ancestry in comparison to Mundani and Bamileke. By contrast, ethnolinguistic groups from Mali (MAL) had much clearer differences in both PC and ADMIXTURE profiles. The Diawando, Fulani and Songhai groups, showed clear deviation from the West African PC cline consistent with their higher Eurasian and Afro-Asiatic-speaker ancestries. Conversely, the Dogon, living in the central plateau region of Mali, speak a very distinct language, often considered as an independent branch of the Niger-Congo language[19]. The Dogon had relatively little ancestry outside of the Niger-Congo group and thus localized closer to other West African populations (**Supplementary Figure 9**). This broad ethnolinguistic diversity and the relatively smaller sample size of MAL led us to exclude them from some of the downstream analyses (admixture dating and selection scans). In Botswana, ADMIXTURE analysis also showed considerable differences in Khoe-San gene flow between ethnolinguistic groups. However, the broad distribution of ethnolinguistic groups limited any systematic analysis of possible differential Khoe-San gene flow between ethnicities.

1.7 - Analysis of runs of homozygosity (ROH)

We further delineated differences in demographic and/or cultural history between our population groups by estimating and comparing the number and length of runs of homozygosity segments (**Supplementary Figure 10**). The MAL group contained the longest ROH segments, not only among populations included in our study, but also in comparison to previously sequenced African populations[11,13]; however, in agreement with the PC and the ADMIXTURE analysis, there were substantial differences in ROH length and frequencies between both individuals from different MAL ethnolinguistic groups and between individuals from the same MAL ethnic group (**Supplementary Figure 11**).

1.8 - mtDNA and Y chromosome haplogroup analysis

We compared the distribution of mitochondrial (mt) DNA haplogroups (assigned using Haplogrep2[20] and Y chromosome haplogroups (assigned using AMY-Tree[21] in our populations (**Supplementary Figure 12**). Compared to other central-west African populations in our dataset BRN had the highest frequency of L3 haplogroups (**Supplementary Figure 12A**). Similarly, the Khoe-San-related mitochondrial haplogroups (L0d and L0k) were found exclusively in Botswana[22] (**Supplementary Figure 12A**). Consistent with previous reports of predominance of the E1b1b haplogroup in the Tuareg[23] from the northern Saharan region of Africa, we also detected the highest frequency of this Y-chromosome haplogroup in MAL (**Supplementary Figure 12B)**.

Supplementary Note 2 - Signatures of selection

2.1 CLR Signals for previously characterized genes

Among the genes previously reported to be under strong selection in African populations, only one was contained within the CLR-based outlier windows (*P*<0.001). This signal consisted of multiple windows around the *HBB* region in the FNB, for which all samples were homozygous for the sickle cell mutation in *HBB*. This signal is thus likely inflated by the excess sampling of long-range haplotypes associated with the strong selection at the sickle cell allele[15]. Therefore, to detect possible signatures of selection at previously characterized genes, we used a more liberal threshold of *P*<0.01 for 'known' loci, which identified windows overlapping genes such as *SYT1*, *APOL1* and *LARGE* as positively selected in our dataset. As with some of the novel signals, the strength of selection signal at *APOL1* and *FOXP2* varied considerably between populations (**Extended Data Figure 2A**).

2.2 CLR outlier windows in non-coding regions

As identified in any selection scan, a large number of outlier signals in our study were found in non-coding regions of the genome; this included contiguous stretches (>100 kb) of non-coding regions. We excluded the HBB region in FNB, as this region was inflated in this population as a result of all individuals being homozygous for the sickle (HbS) mutation, which is known to have a strong signal of recent selection[24]. The length of these noncoding stretches as well as the strength of selection signal at these loci varied considerably between populations (**Supplementary Table 5)**; e.g. a stretch of adjacent windows on chromosome 16 was detected in all populations, whereas a stretch of windows on chromosome 7 was limited to the Berom (BRN) and Western Gur (WGR) groups only (**Figure 4A, Supplementary Table 5**).

Because most GWAS hits are found in non-coding regions, we considered whether some of our outlier non-coding loci might map to GWAS gene hits for specific disease traits. To evaluate this, we used the GWAS catalog[25] to determine genome-wide significant SNPs within our non-coding outlier loci (**Methods**). Thirty-six regions contained at least one significant GWAS hit, and these mapped to 24 catalogued disease traits (**Supplementary Table 9; Supplementary Figure 14)**. Five non-coding regions included one or more GWAS hit in two or more populations, and included genes mapped to eye morphology (*NR3C2*, *KAZALD1*), chronic kidney disease (*RF00019 - AC026320.2*), uterine fibroids (*RNU6-931P - RN7SKP199*), and variable red cell indices (*CDA*); the latter 3 traits have a higher prevalence among individuals of African ancestry[26-28].

Recent studies have also emphasized the effect of non-coding variants on gene regulation, especially transcription[29]. Of the non-coding outlier loci, 88 (58%) had at least one expression quantitative trait locus (eQTL) among the 49 GTEx tissues (**Supplementary Figure 15**). Whole blood, EBV-transformed lymphocytes, skin (exposed and unexposed), and heart (left ventricle) had significantly more eQTLs than expected (t-score >1.65, P <0.05, T-distribution, df=999, **Methods, Supplementary Figure 16)**. This was true across all the populations surveyed, except Batswana (BOT) and Western Gur speakers (WGR) (**Supplementary Figure 17**). eQTLs in whole blood and skin, given their relative ease of sampling, are well-characterized relative to other tissues, and this may have driven the preponderance of observed regions with eQTLs in these tissues. The relevance of other non-coding outlier regions may become clearer as catalogues of eQTLs in other tissues improve and include more diverse populations.

2.3 Population branch statistics (PBS) analysis

Given major differences in environment, diet, pathogenic load and non-Niger-Congo gene flow in these populations, we used a Population Branch Statistic (PBS)-based approach[30] to detect genomic regions that have been selected only in Southern, Central and Western Africa. This analysis, as implemented in previous

studies, was restricted to protein coding exons[30,31] and included SNVs with a minimum MAF of 1% across all populations. Considering Botswana (BOT) as the representative south African population, West African Gur speakers (WGR) as the representative west African population and the Chinese Han from Beijing (CHB) from the 1000 Genomes Project[7] as the outlier population, we identified exons showing longer branch length in BOT (BOT-WGR-CHB), corresponding to an empirical P< 0.001 (**Extended Data Figure 2B**, **Supplementary Table 11).** With the exception of *CCM2* (associated with blood vessel development, heart development, inner ear development and stress-activated MAPK cascade) and *MCRS1* (associated with DNA repair), which were represented by signals from more than one exon, other exons with outlier scores corresponded to single genes. The latter group included *TYR* - a gene involved with pigmentation that has been reported to be under selection in a recent study of southern African populations[32], *VDAC3* (associated with Hepatitis B infection), and *LAMA5* (associated with Human papillomavirus infection). To further bolster these observations, we also estimated the PBS between BOT and CAM (representing central west Africa) with CHB as outlier (BOT-CAM-CHB) (**Extended Data Figure 2B , Supplementary Table 12**) and focused on signals found in both analyses. The latter group included *PLAT*, encoding the tissue plasminogen activator (TPA), which has a broad range of roles, most notably tissue homeostasis. Other genes detected as outliers in the BOT-CAM-CHB analysis (P< 0.001) and having a high PBS in BOT-WGR-CHB (P< 0.01) included viral immunity related genes such as *HM13* (associated with Hepatitis C), *CD44* (associated with Epstein-Barr virus infection), and *VPS37C* (involved in viral protein processing).

Use of the PBS statistic for identifying selection signals unique to West Africa (comparing WGR to BOT (WGR-BOT-CHB) and CAM (WGR-CAM-CHB) detected multiple exons with outlier scores in *MPHOSPH9* (**Supplementary Table 13**). Little is known about this gene, but the strength of its selection signature argues for additional study, especially in a West African context. Other signals detected in this analysis include *TRAF1* (involved in regulation of apoptosis), *CORO1C* (involved in phagocytosis), *DHX58* (involved in response to viruses), *FAN1* (involved in DNA repair) and *EPX* (involved in defence response to nematodes) (**Supplementary Table 13**). Comparison of signals between WGR-BOT-CHB and WGR-CAM-CHB showed five common signals (including *FAN1* and *RAB5C*) suggesting that these genes are strong candidates for West-African specific selection (**Extended Data Figure 2B**).

Six regions were outliers (P<0.001) in both CAM-BOT-CHB and CAM-WGR-CHB analyses (**Extended Data Figure 2B; Supplementary Table 14**) and thus suggestive of Central-West African selection. One of these signals, *SERPINA1* has been linked to several key biological functions, including blood coagulation, inflammation, and response to chromate, cytokine, estradiol, hypoxia, lead, and triglycerides. Studies have suggested a specific inhibitory role of this protein in a protozoan (*Cryptosporidium parvum*) infection, and suppression of bacterial infection by *Pseudomonas aeruginosa* and *Staphylococcus*

aureus[33,34]. Moreover, a specific 20-residue fragment of the encoded protein (C-terminal peptide, residues 377–396, referred to as VIRIP) has been shown to bind to the gp41 fusion peptide of Human Immunodeficiency Virus, Type 1 (HIV-1) and prevent the virus from entering target cells, suggesting a role of this protein in inhibiting HIV-1 infection[35].

Supplementary Note 3 – Site-frequency-spectra and putative loss-of-function variants

Population genetics theory predicts that low frequency alleles will be relatively enriched for deleterious mutations as a consequence of purifying selection. Accordingly, the proportion of singleton putative loss-of-function (pLoF; 50%) and damaging variants (35%) was significantly more than that for variants predicted to have little effect on the resulting protein (25%) (**Methods**; Wilcoxon signed-rank test $P<1x10^{-7}$ for all populations). As observed with novel variants and HDVs, pLoF variants were most abundant among participants from BOT (**Extended Data Figure 3B**), likely again reflecting their KS ancestry and consistent with exome sequencing reports from the same population[36]. Putatively deleterious SNVs were also relatively enriched among doubleton and tripleton variants (**Supplementary Figure 18**), which, given the modest sample sizes at the population-level, suggested the potential for common protein-damaging variants in the populations surveyed. To provide additional context for this, we focused on population-shared pLoF variants, which are more likely to be common. The majority of shared pLoF variants were shared across all populations (**Extended Data Figure 3B**) rather than with just neighbouring groups, suggesting that they are particularly old variants. Shared-all pLoFs were enriched in genes from five disease-gene categories, including metabolic disorders (**Extended Data Figure 3C**). Several genes in the latter category are known to cause recessive Mendelian disorders (e.g. *TPK1* - Thiamine episodic encephalopathy, MIM#614458; *PFKM* - Glycogen storage disease type 7, MIM#232800; **Supplementary Figure 19**); however, the appreciable allele frequencies of many of the constituent pLoFs make it unlikely that these are truly disease-causing (see below), and might instead represent artefacts, neutral variants, or confer a second, as yet undiscovered, biological function.

Supplementary Note 4 – ClinVar pathogenic variants in the H3Africa dataset

The 262 unique variants annotated as pathogenic or likely pathogenic with a minor allele frequency below 5% in gnomAD populations included a reportedly pathogenic missense variant (c.(653C>T), p.(Thr218Ile)) in *LDLRAP1 (MIM# 605747*), causative of autosomal recessive familial hypercholesterolemia, that was found at 12.5% in BOT and 8% in CAM and DRC (but was absent from African 1000G populations except LWK; **Supplementary Figure 20**), as well as a missense variant (c.(233A>C); p.(Asn78Thr)) in *ZEB1* (MIM# 189909; autosomal dominant Fuchs endothelial corneal dystrophy 6) that has a frequency between 6.1% and 9.7% in west African populations (**Figure 4C**,

Extended Data Figure 4A; Supplementary Figure 20). Although corroborating epidemiological data are limited, the incidence of these diseases in African populations is not known to be correspondingly high[37,38]. We interpret the high allele frequencies observed as suggestive of variant classification errors, with inaccurate inferences of pathogenicity in ClinVar and other databases.

Supplementary Note 5 - Ethics statements from participating studies

The H3Africa Consortium (H3A-Baylor) Samples

Benin: Ethics approval for the study was granted by the Sainte-Justine Research Center Ethics Committee and by the Faculté des Sciences de la Santé of the University of Abomey-Calavi in Benin, West Africa.

Berom (Nigeria): Berom samples were collected as part of the ACCME cohort, which is located in Nigeria. Ethical approval to conduct this study was obtained from the National Health Research Ethics Committee in Nigeria.

Botswana: Approvals for the study were obtained from the institutional review boards of each of the participating institutions in the Collaborative African Genomics Network (CAfGEN), including the Health Research and Development Committee of the Ministry of Health in Botswana, and the University of Botswana in Gabarone, Botswana.

Cameroon: The study was approved by the University of Cape Town, Faculty of Health Sciences Human Research Ethics Committee (HREC REF: 661/2015), Cape Town, South Africa; and the National Ethics Committee of the Ministry of Public Health, Yaounde, Republic of Cameroon (No. 033/CNE/ DNM/07). All patients older than 18 years signed consent forms, while informed consent was given by the parents or guardians for participants younger than 18 years old, in accordance with the declaration of Helsinki.

Mali: Approved by the Ethical Committee at the Faculty of Medicine and Odontostomatology (FMOS), University of Bamako.

AwiGen Collaborative Centre of the H3Africa Consortium samples (Ghana, Burkina Faso):
The AWI-Gen study protocol, information sheet and informed consent documents, tailored to the local context and including translation into various local languages, was approved by the Human Research Ethics Committee of the University of the Witwatersrand (Protocol Number: M121029). In addition, each of the HDSS centres obtained ethics approval according to their respective institution and country-specific rules and regulations.

The TrypanoGEN Collaborative Centre of the H3Africa Consortium

Ethical approval was provided by the national ethics councils of each of the TrypanoGEN participating countries: Cameroon (2013/364/L/CNERSH/SP), Democratic Republic of Congo (No 1/2013), Guinea (1-22/04/2013), Cote d'Ivoîre (2014/No 38/MSLS/CNER-dkn), Malawi (1213), Uganda (HS 1344), and Zambia (011-09-13).

Southern African Human Genome Programme (SAHGP)

The study was approved by the Human Research Ethics Committee (HREC; Medical) of the University of the Witwatersrand, Johannesburg (Protocol number: M120223).

SUPPLEMENTARY FIGURES

Supplementary Figure 1 – Extended principal components (PCs) of H3Africa populations from WGS data. Sampled populations are shown alongside existing African WGS data from AGVP[11], 1000 Genomesproject[7] and SAHGP[39]; n=1,253 biologically independent samples. (A) PC3 and PC4; (B) PC5 and PC6.

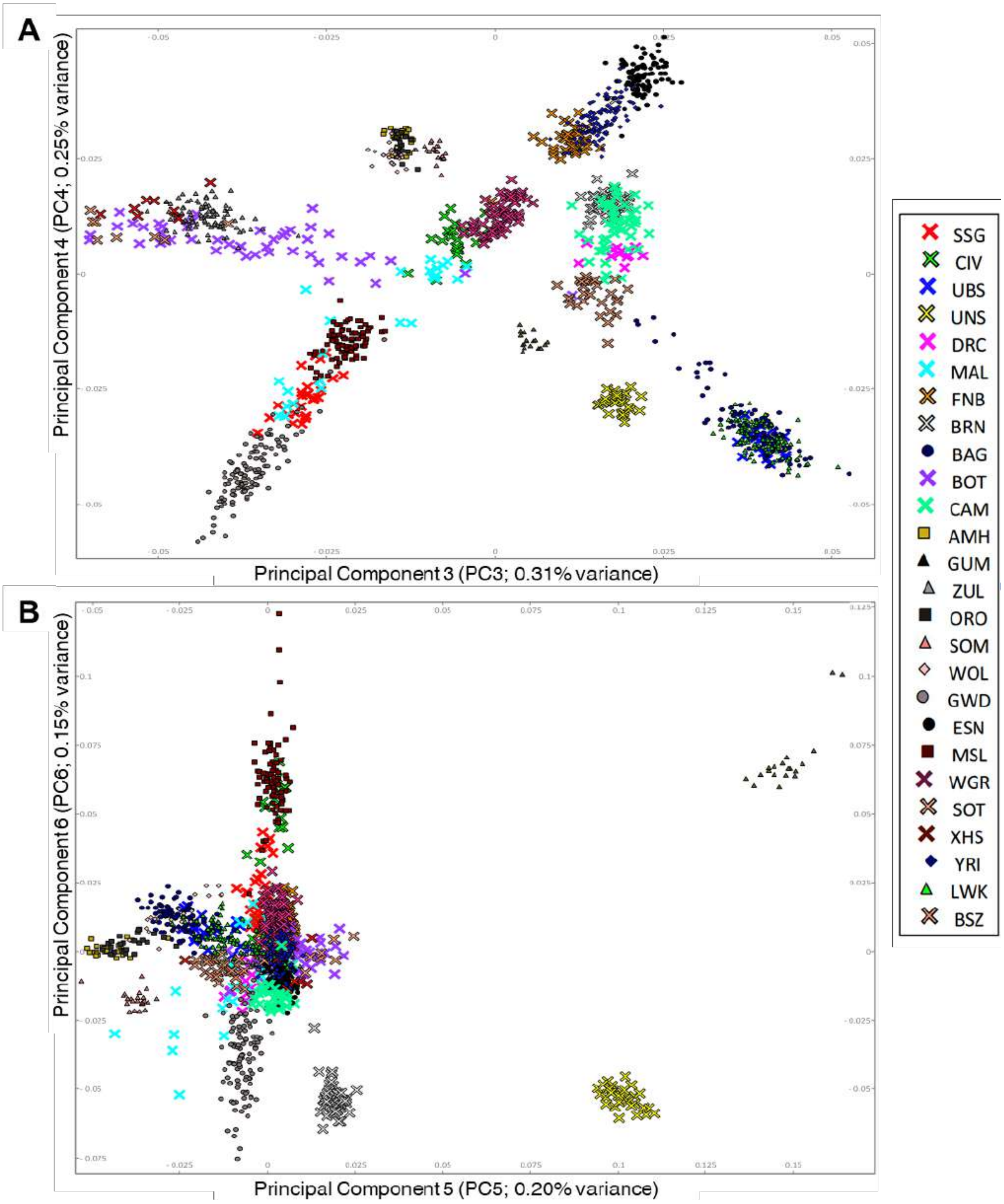

Supplementary Figure 2 – PC based evaluation of novel WGS extended to include data from Schlebusch et al.[12] and Patin et al.[15].

Comparison dataset includes data from Supplementary Figure 1 n=2,838. (**A**) PC1 and PC2 (**B**) PC3 and PC4 (**C**) PC5 and PC6.

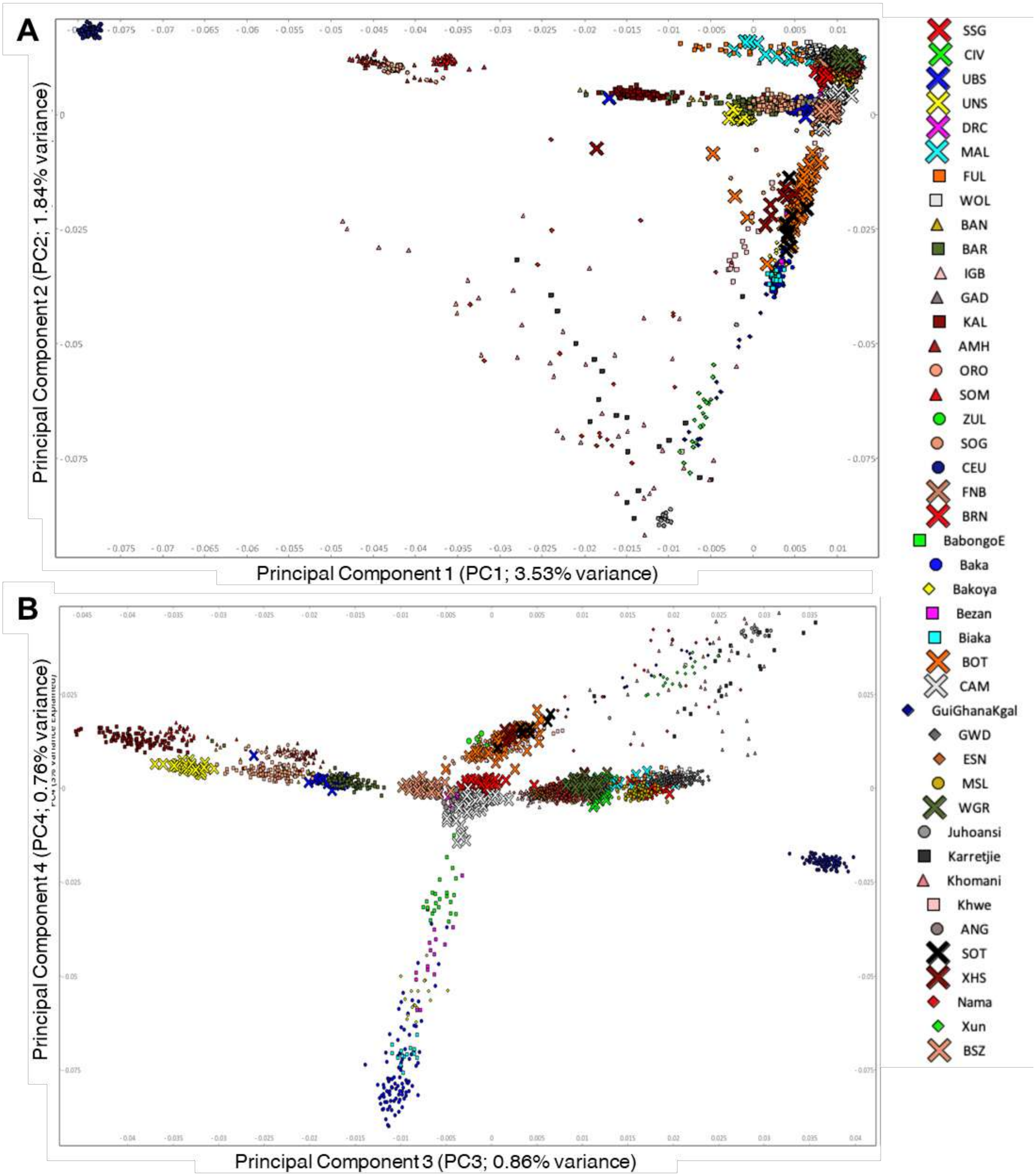

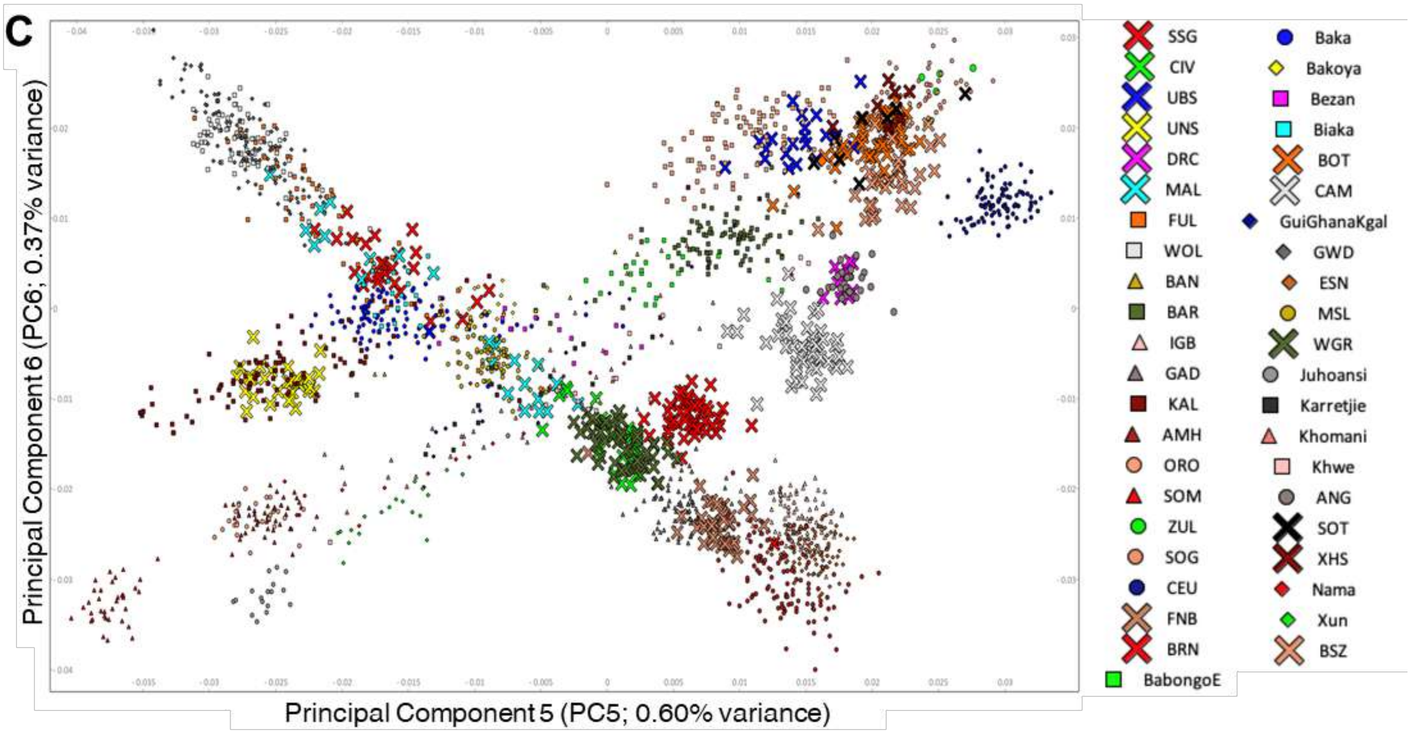
C
Principal Component 6 (PC6; 0.37% variance)
Principal Component 5 (PC5; 0.60% variance)
SSG
CIV
UBS
UNS
DRC
MAL
FUL
WOL
BAN
BAR
IGB
GAD
KAL
AMH
ORO
SOM
ZUL
SOG
CEU
FNB
BRN
BabongoE
Baka
Bakoya
Bezan
Biaka
BOT
CAM
GuiGhanaKgal
GWD
ESN
MSL
WGR
Juhoansi
Karretjie
Khomani
Khwe
ANG
SOT
XHS
Nama
Xun
BSZ

Supplementary Figure 3 - Correlation between Genetic and Geographic Distances using PROCRUSTES.

(**A**) Heatmap of pairwise F_{ST} estimates in joint dataset; (**B**) Principal Coordinates Analysis based on F_{ST} for all populations (n=26 samples from 16 countries); (**C**) Principal Coordinates Analysis based on great circle distances between country midpoints for all populations; (**D**) Principal Coordinates Analysis based on F_{ST} for NC speakers (n=20 samples from 15 countries); (**E)** Principal Coordinates Analysis based on great circle distances between country midpoints for NC speakers only.

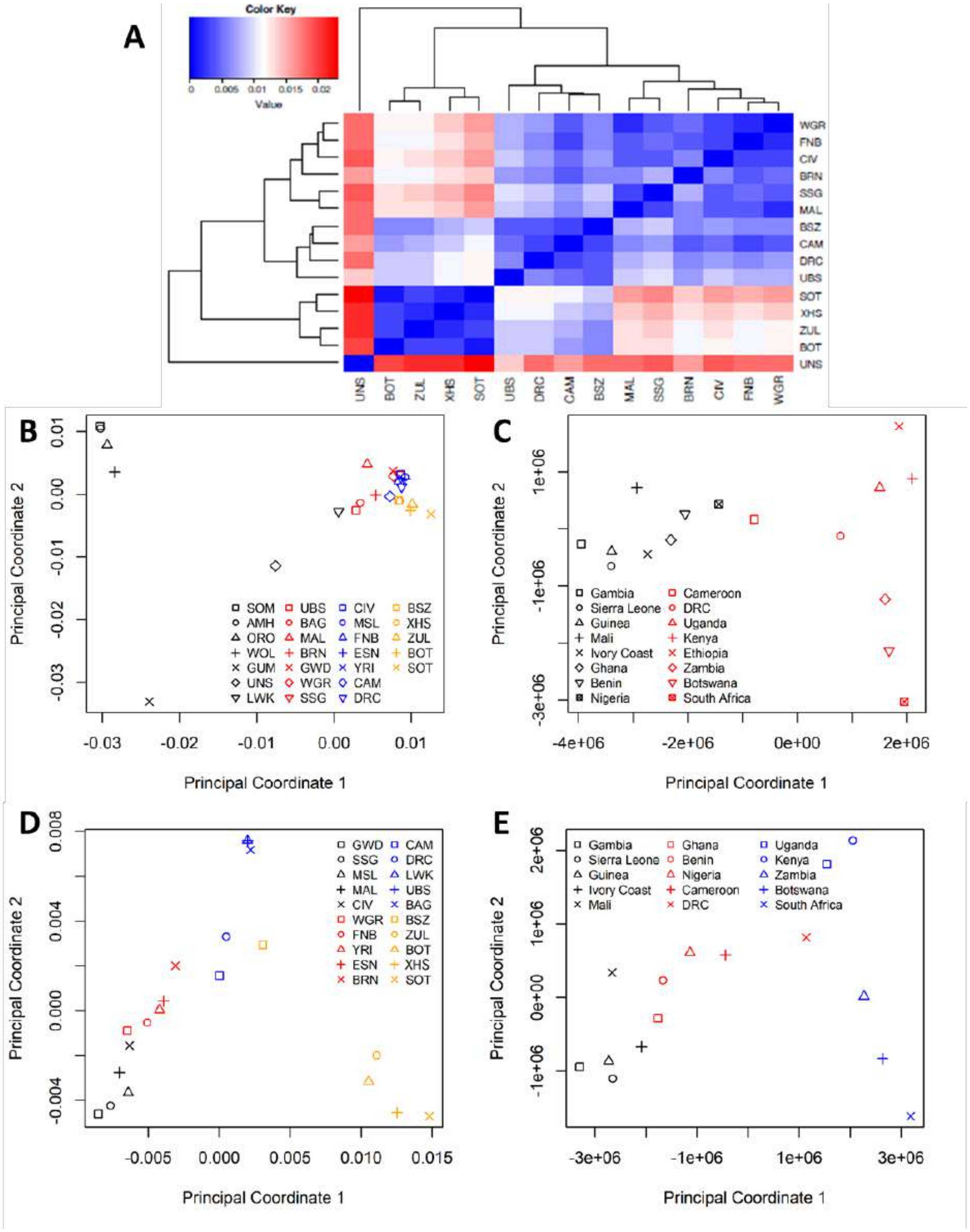

Supplementary Figure 4 - Cross validation error scores for ADMIXTURE clustering.

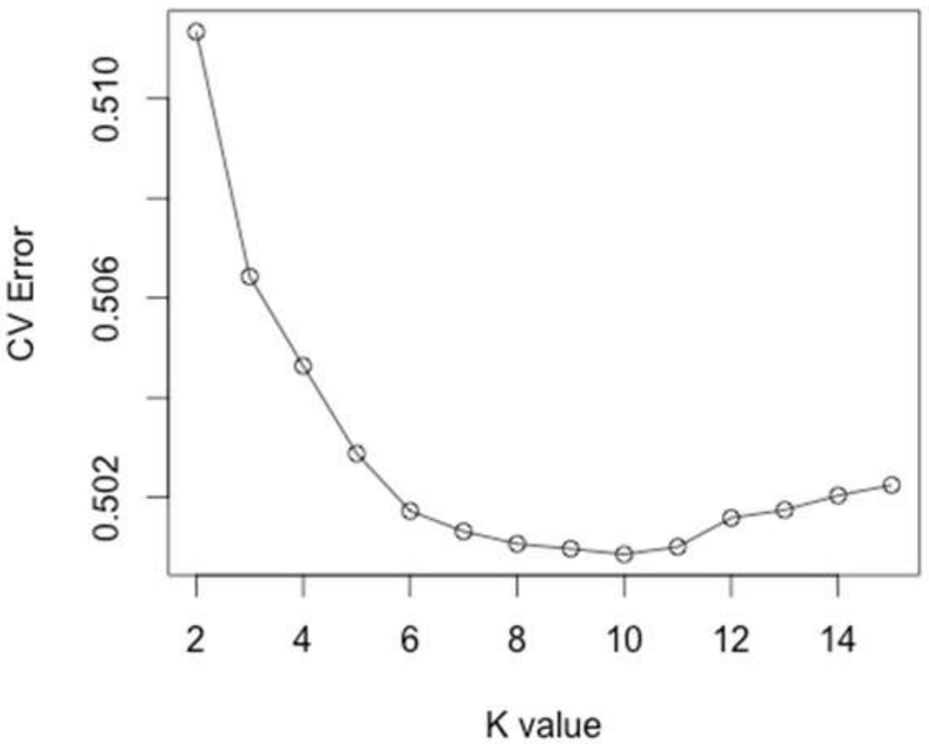

Supplementary Figure 5 - PC and ADMIXTURE clustering analysis of Ugandan Nilo- Saharan (UNS).

East-African populations from Sudan[3] are used as reference. (**A**) Locations of Ugandan Nilo-Saharan speakers (UNS) (Lugbara) and Bantu-speakers (UBS) (Basoga) in Uganda; (**B**) PCA showing UNS to localize independently of NS-speakers from Kenya (represented by Masai, Kalenjin), Ethiopia (Gumuz) and Sudan (Nuba, Bari, Dinka, Gemar, Nuba, Nuer, Shilluk, and Zagawa); Amhara, n=42; Baria, n=5; BRN, n=44; Danagla, n=15; Dinka, n=16; DRC, n=12; Gaalien, n= 15; Gemar; n= 7; Hadendowa; n=11; Hausa, n=6; Kalenjin, n=40; Kikuyu, n=40; Mahas, n=15; Nuba, n=16; Nuer, n=15; Oromo, n=26; Shilluk, n=16; UBS, n=21; UNS, n=30; Zagawa, n=6. AA=Afro-asiatic speakers; NS=Nilo-Saharan speakers; BS= Bantu-speakers; (**C**) ADMIXTURE analysis (at *K*=5) shows the UNS to harbour the highest Rain Forest Forager (RFF) ancestry among NS-speaker populations (includes Mbuti (n=13) from Patin *et al.*[15].). Maps were created using R[40]. Country border data was obtained from: http://thematicmapping.org/downloads/world_borders.php

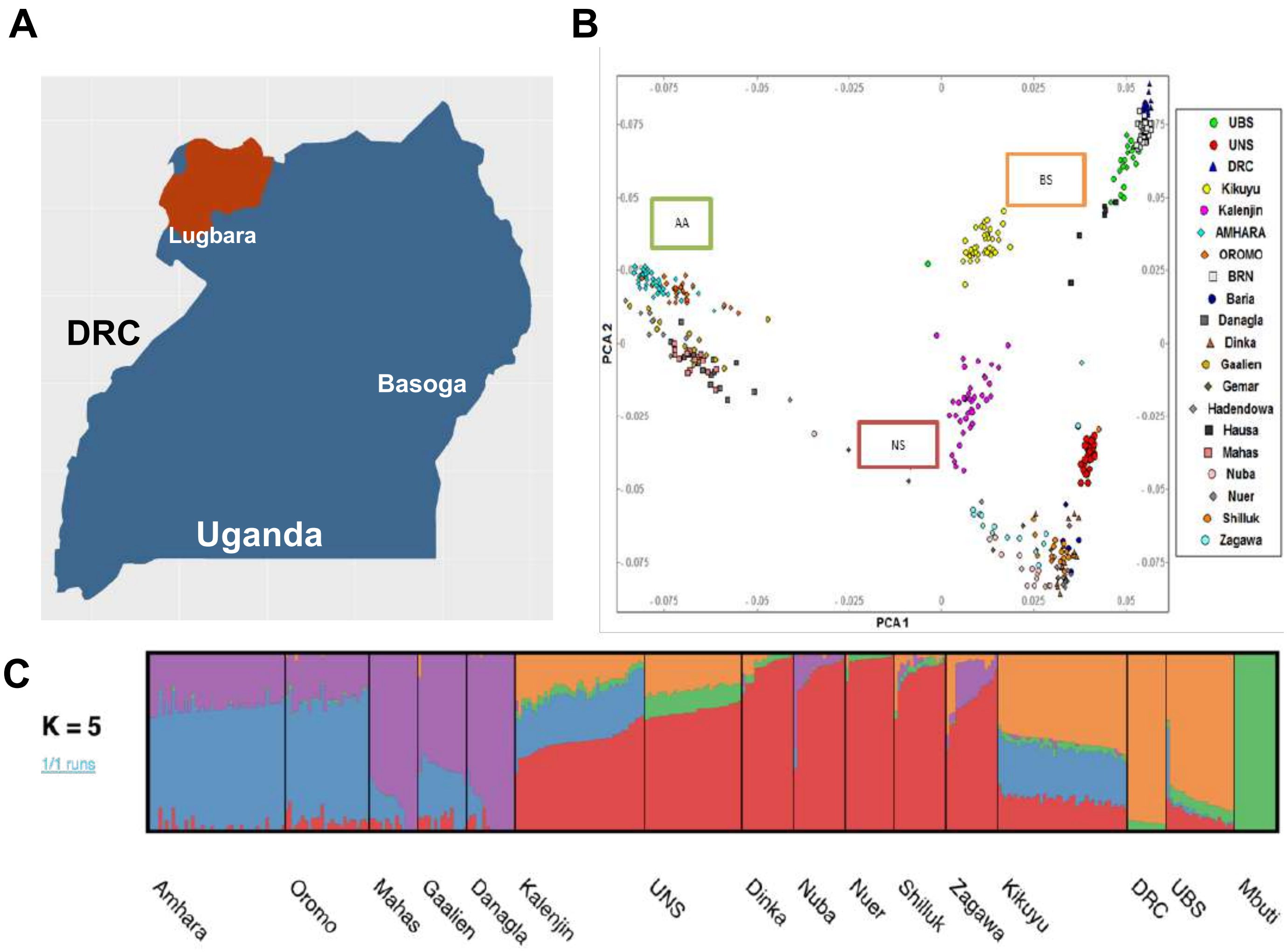

Supplementary Figure 6 - Genetic affinities and ancestral composition of Berom (BRN). Additional West African data[4,6-8,41] are included to facilitate estimation. (**A**) Principal component analysis, in concordance with geographic distributions, shows the BRN to localize between Yoruba and Hausa; (**B**) Ancestry proportions derived from ADMIXTURE analysis (at *K*=9) shows a higher East African ancestry component in BRN compared to groups such as Hausa and Fulani; (**C**) Geographic location within Nigeria of BRN and other Nigerian populations included in our analysis. Sample sizes: Berom (n=44), Esan (n=99), Yoruba (n=108), Hausa (n=12), Mada (n=12), Bulala (n=15), Fulani (n=25), Sara (n=62), Tubu (n=73), Anuak (n=22), Gumuz (n=17), Oromo (n=21), and South Sudanese (n=23). Maps were created using R[40]. Country border data was obtained from: http://thematicmapping.org/downloads/world_borders.php

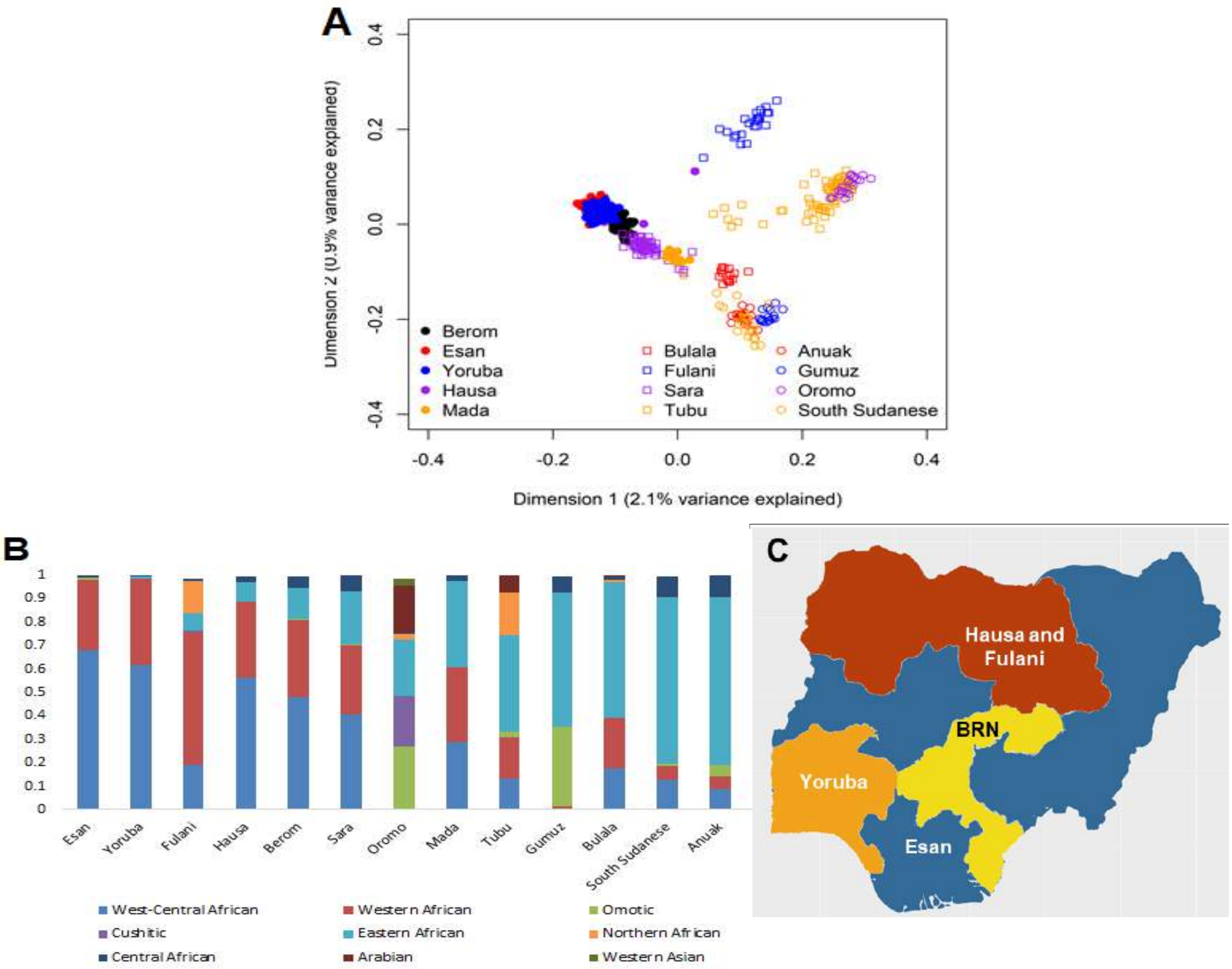

Supplementary Figure 7 - Admixture masking based assessment of non-NC speaker gene flow. (**A**) Principal component analysis showing WGS data based on a randomly downsized set (~150K SNPs) (**B**) PCA in which regions with >20% KS ancestry (identified using RFMIX version 2) are masked (**C**) PCA with regions with high NS-speaker ancestry (>20%) masked. Sample sizes: BOT n=41; FNB n=37; CAM n=45; BRN n=44; WGR n=54; BSZ n=29; DRC n=12; UNS n=30; UBS n=21.

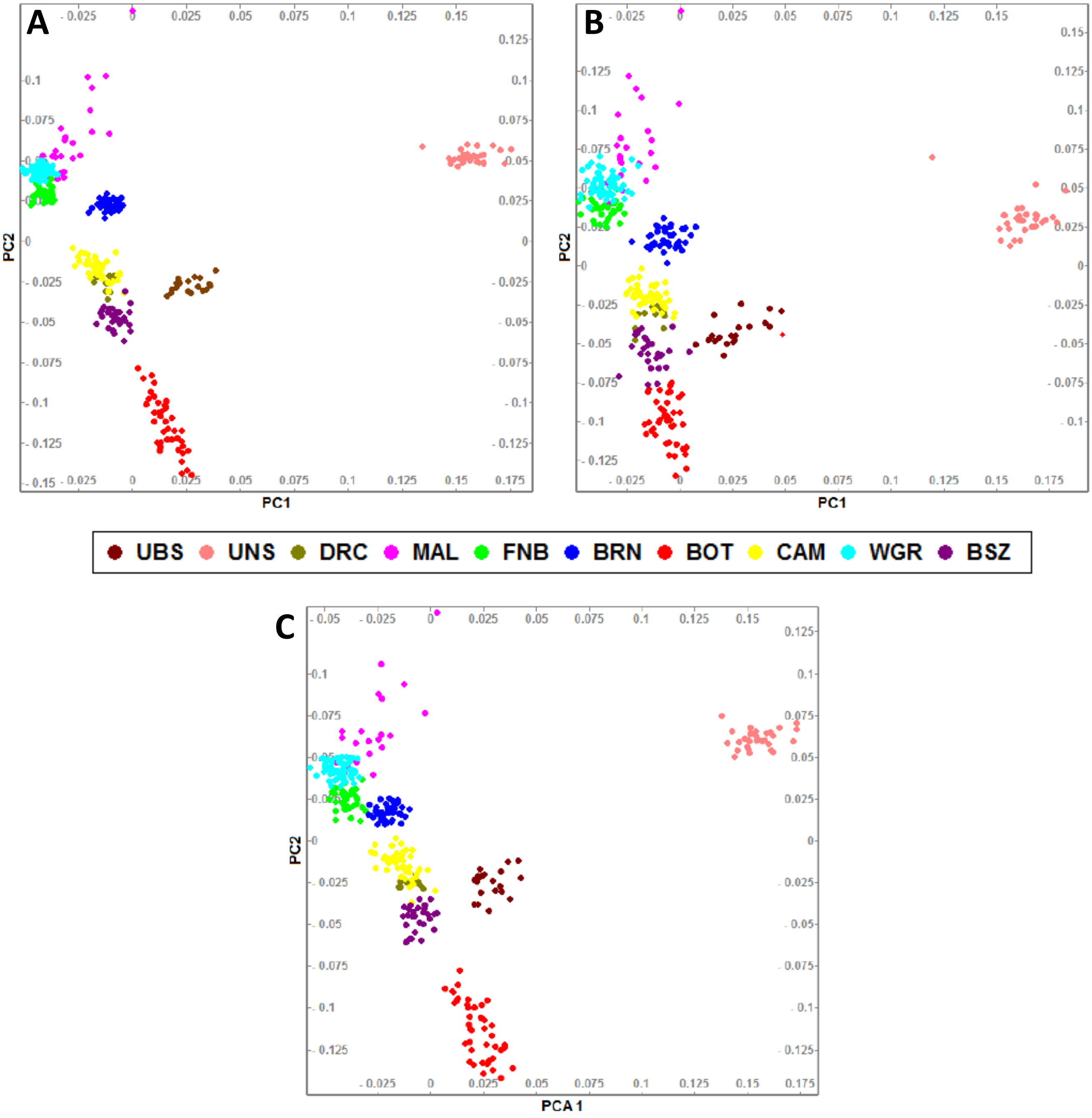

Supplementary Figure 8 - Estimates of genetic affinities of the Zambian (BSZ) population

(**A**) Principal component analysis shows BSZ in comparison to other Central-African populations (such as DRC and ANG), is closer to both South and East African Bantu-speakers (**B**) Heatmap summarizing level of IBD sharing between populations. (**C**) IBD sharing distance relative to BOT (**D**) IBD sharing distance relative to UBS. (**E)** IBD sharing distance relative to BSZ. Sample sizes: BOT n=41; FNB n=37; CAM n=45; BRN n=44; WGR=54; BSZ n=29; DRC n=12; UNS n=30; UBS n=21; MAL n=24; ANG n=41; YRI n=100. Boxplots show the median of the distribution (central line), with the top and bottom of the box indicating the third quartile (Q3) and the first quartile (Q1), respectively. Whiskers extend to minimum (Q1- 1.5 IQR) and maximum (Q1+ 1.5 IQR).

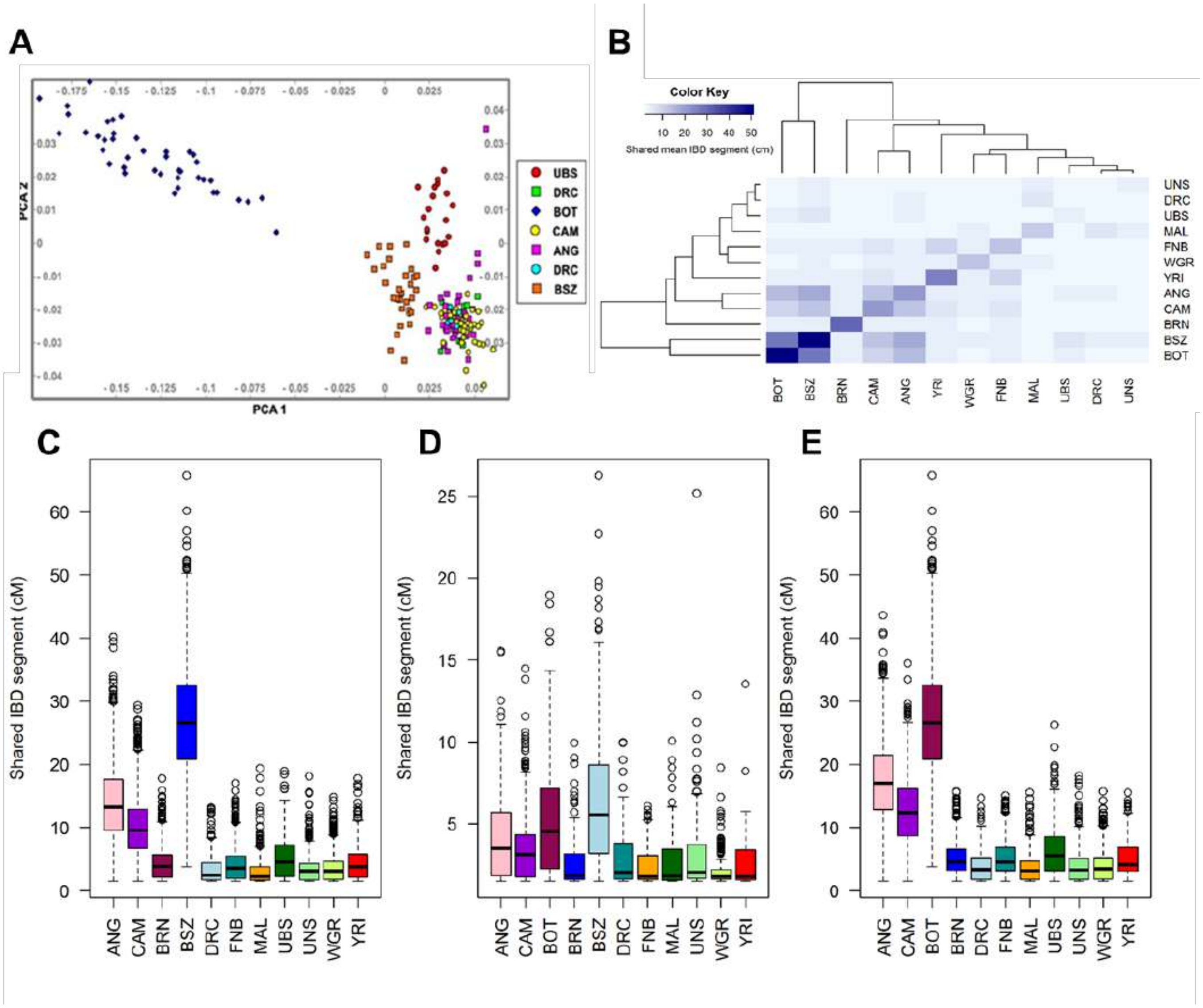

Supplementary Figure 9 - Principal component and ADMIXTURE clustering analysis of ethnolinguistic groups

(**A**) PC analysis at the ethnolinguistic level (n=429 independent samples). Ethnic groups constituting a population are shown in the same colour but different shapes. BOT is shown as a single group for clarity. ADMIXTURE plots showing varying ancestral compositions in ethnolinguistic groups comprising (**B**) CAM (**C**) BSZ (**D**) MAL and (**E**) BOT. The colour codes for B, C, D, and E are the same as in Supplementary Figure 4.

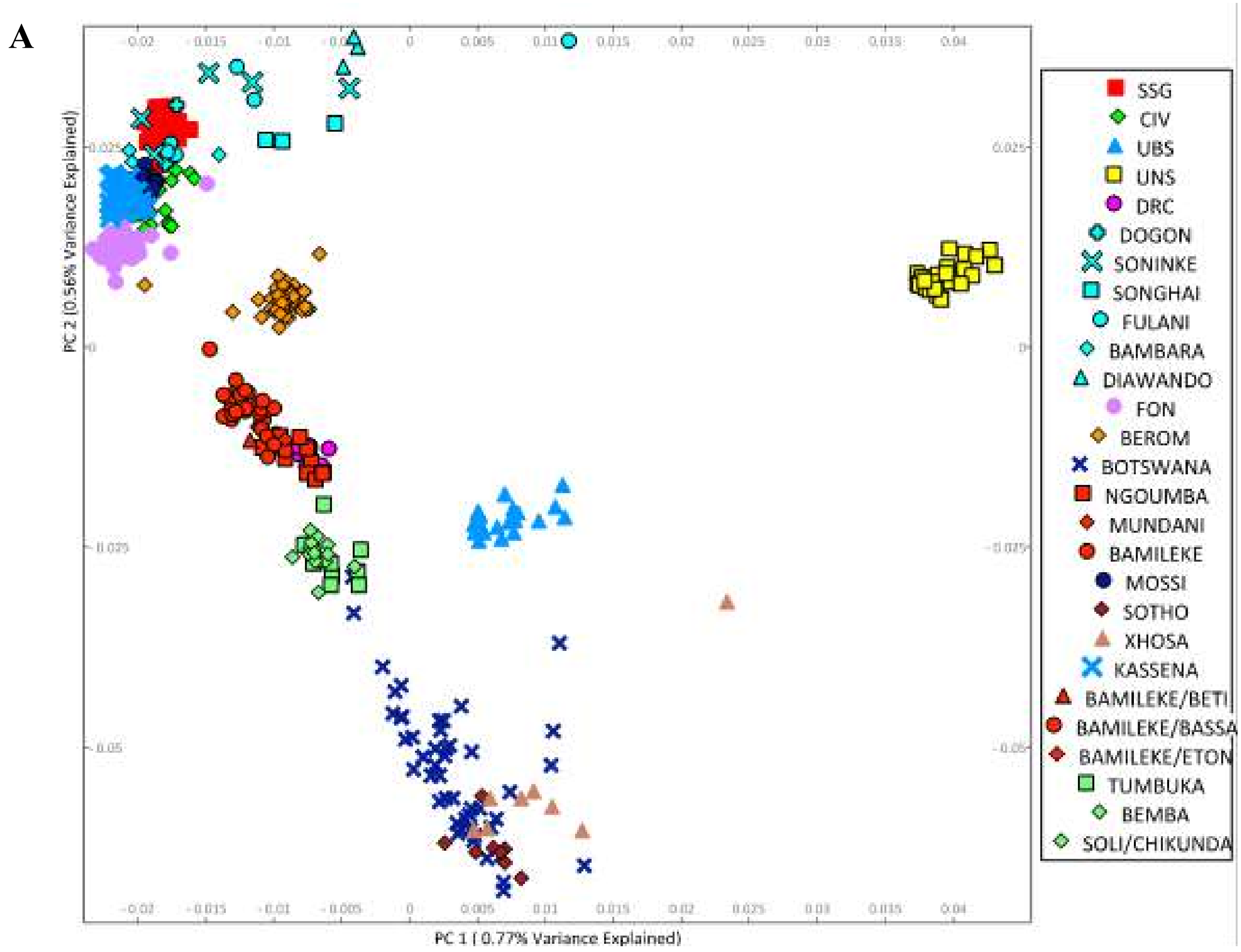

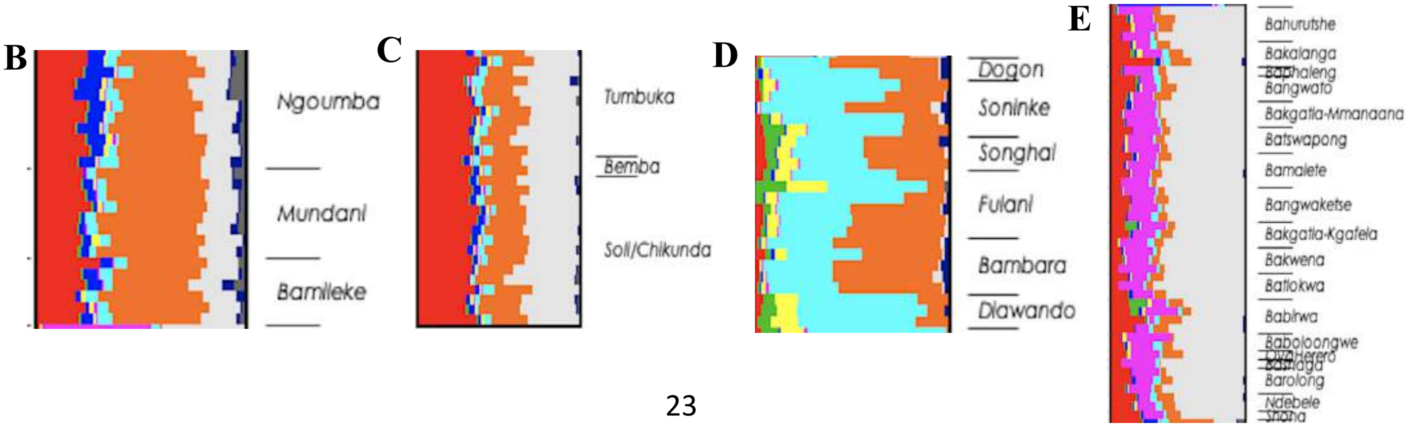

Supplementary Figure 10 - Distribution of runs of homozygosity (ROH) segments in representative African populations.

(**A**) Total ROH length and number of segments in African populations (estimated with default PLINK parameters), the Somali (SOM) is based on AGVP and Yoruba (YRI) is based on the KGP dataset. A modified PLINK parameter set (--homozyg-kb 300 + --homozyg-window-het 3) was used to obtain a better homogenization between low- and high-depth datasets. (**B**) Total ROH length and number of segments based on modified PLINK parameters. Only a subset of representative populations have been shown for clarity.

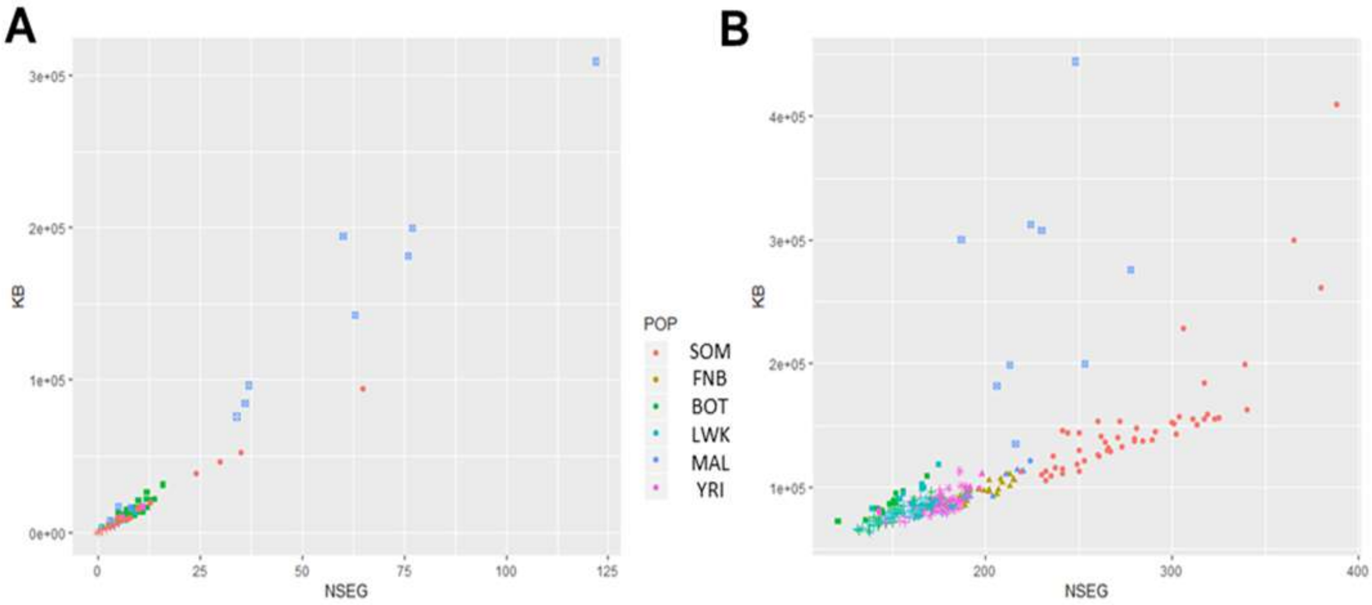

Supplementary Figure 11 - Distribution of total lengths of (A), short (<500kb) (B), medium (500kb to 1.5MB) and (C) long (>1.5MB) ROH in MAL individuals.

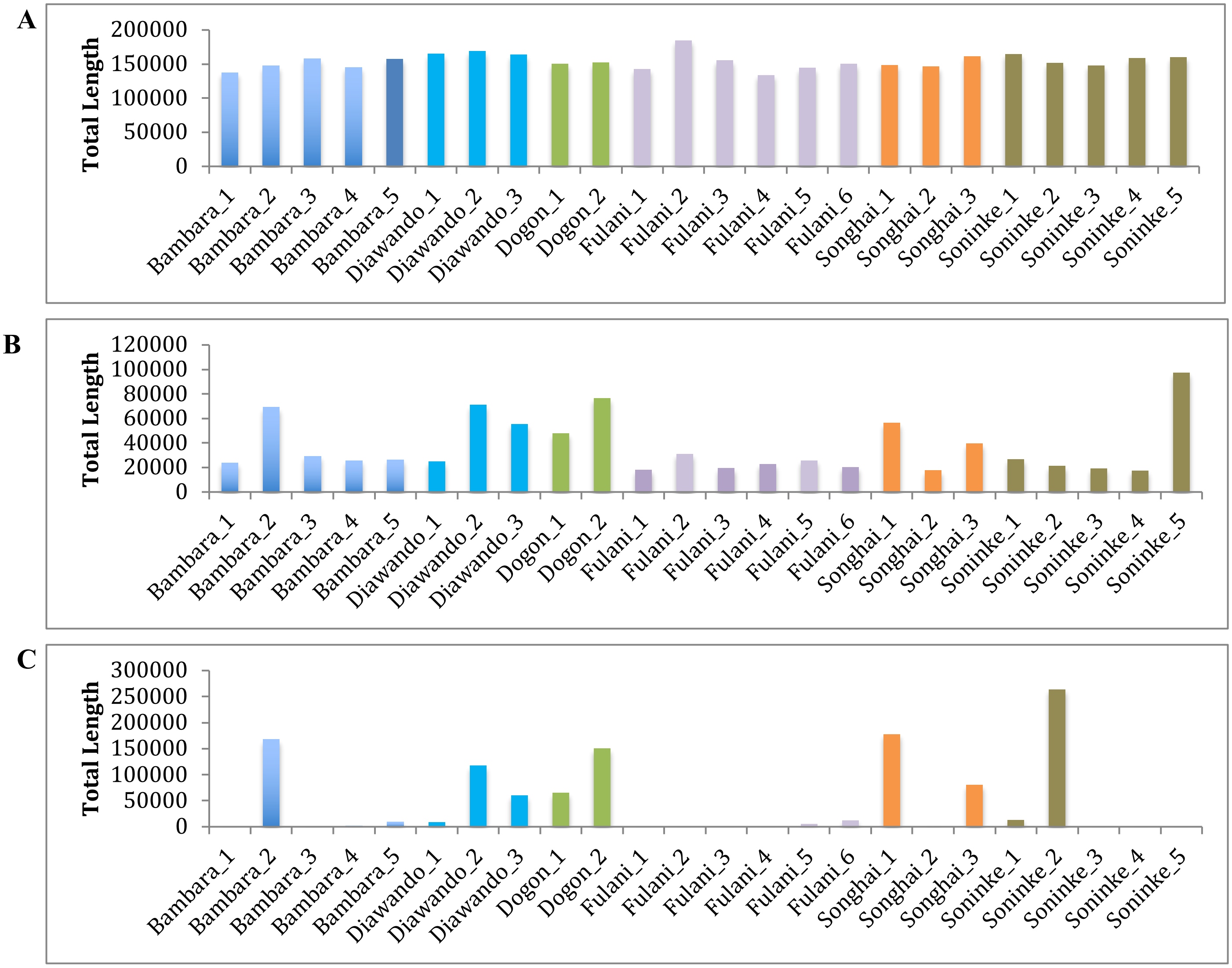

Supplementary Figure 12 - Distribution of mitochondrial and Y chromosome haplogroups in H3A-high coverage WGS samples.

Pie charts show the relative frequencies of (**A**) Mitochondrial haplogroups (**B**) Y-chromosome haplogroups in the populations surveyed. All samples from BRN were female. Maps were created using R[40]. Country border data was obtained from: http://thematicmapping.org/downloads/world_borders.php

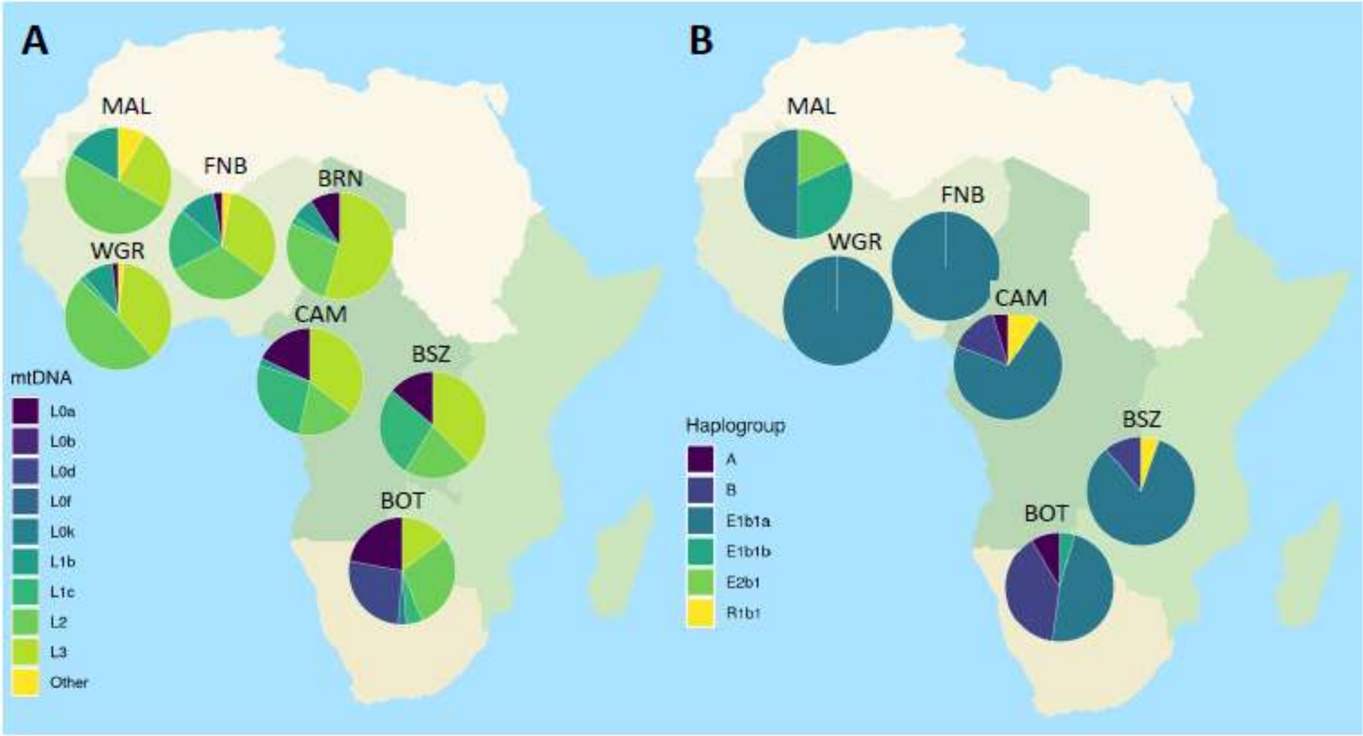

Supplementary Figure 13 - Variant sharing between populations in the H3A high coverage WGS dataset.

Each bar in the bar plots shows the number of variants shared between the populations; sharing is indicated by black dots linked by a solid line in the bottom of the bar plot (upset plot). Each possible population grouping is displayed in the upset plot. On Y axis, the horizontal bars show the total number of variants in each population that are shared with other population(s); (**A**) Novel variants; (**B**) putative loss-of function variants (pLOFs).

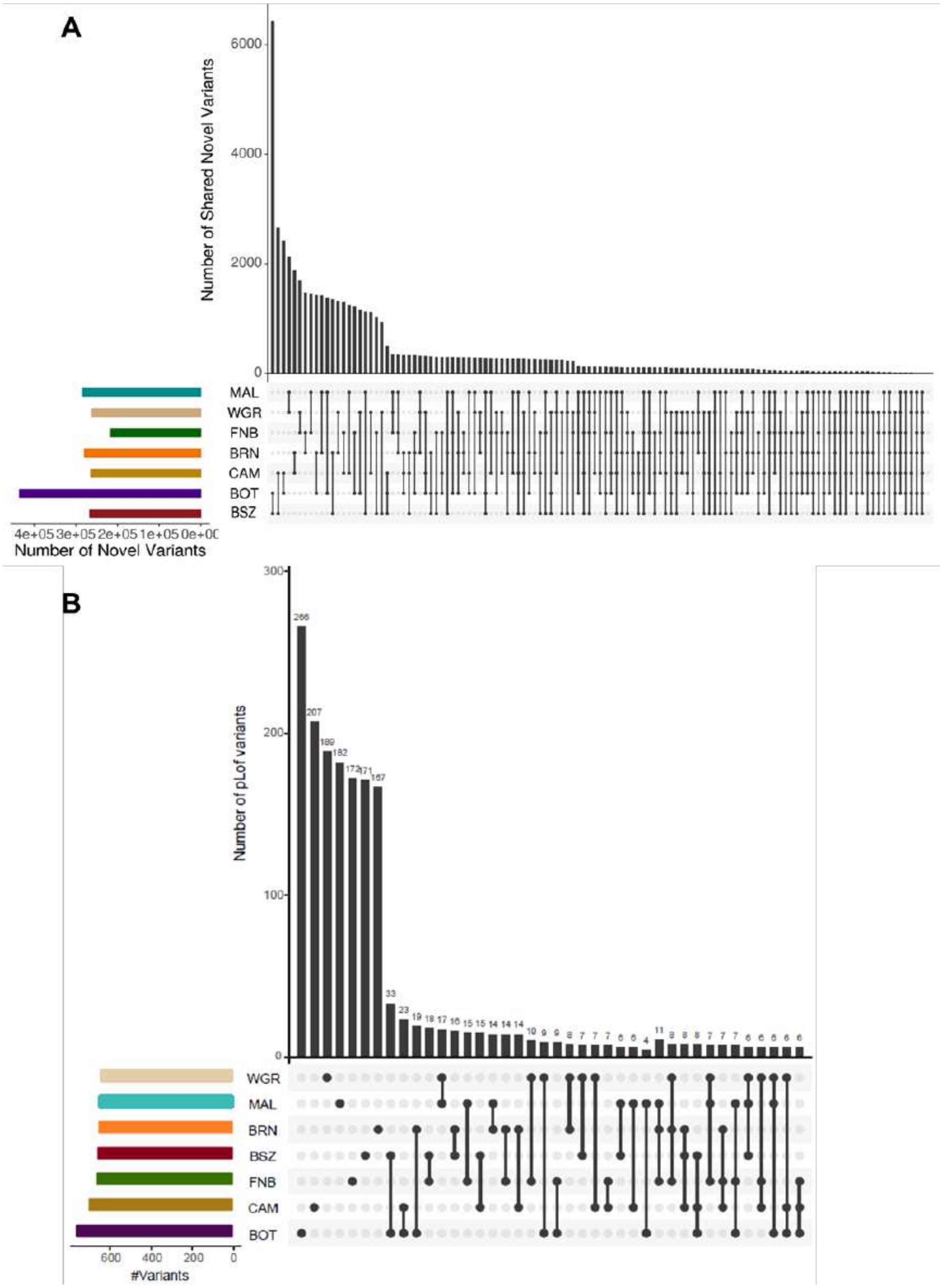

Supplementary Figure 14 - Map of significant GWAS hits found in non-coding regions with outlier CLR selections scores.

Each ring is a different population of sample size n=24 (from outer ring: green – FNB; purple – BOT; Brown – CAM; Orange – BRN; yellow – WGR; red – BSZ), and each dot represents a genome-wide significant GWAS SNP (*P*< 0.5 x 10^{-8}) from the GWAS catalogue found in a non-coding outlier region. Highlighted genes had SNPs that were observed in more than two populations.

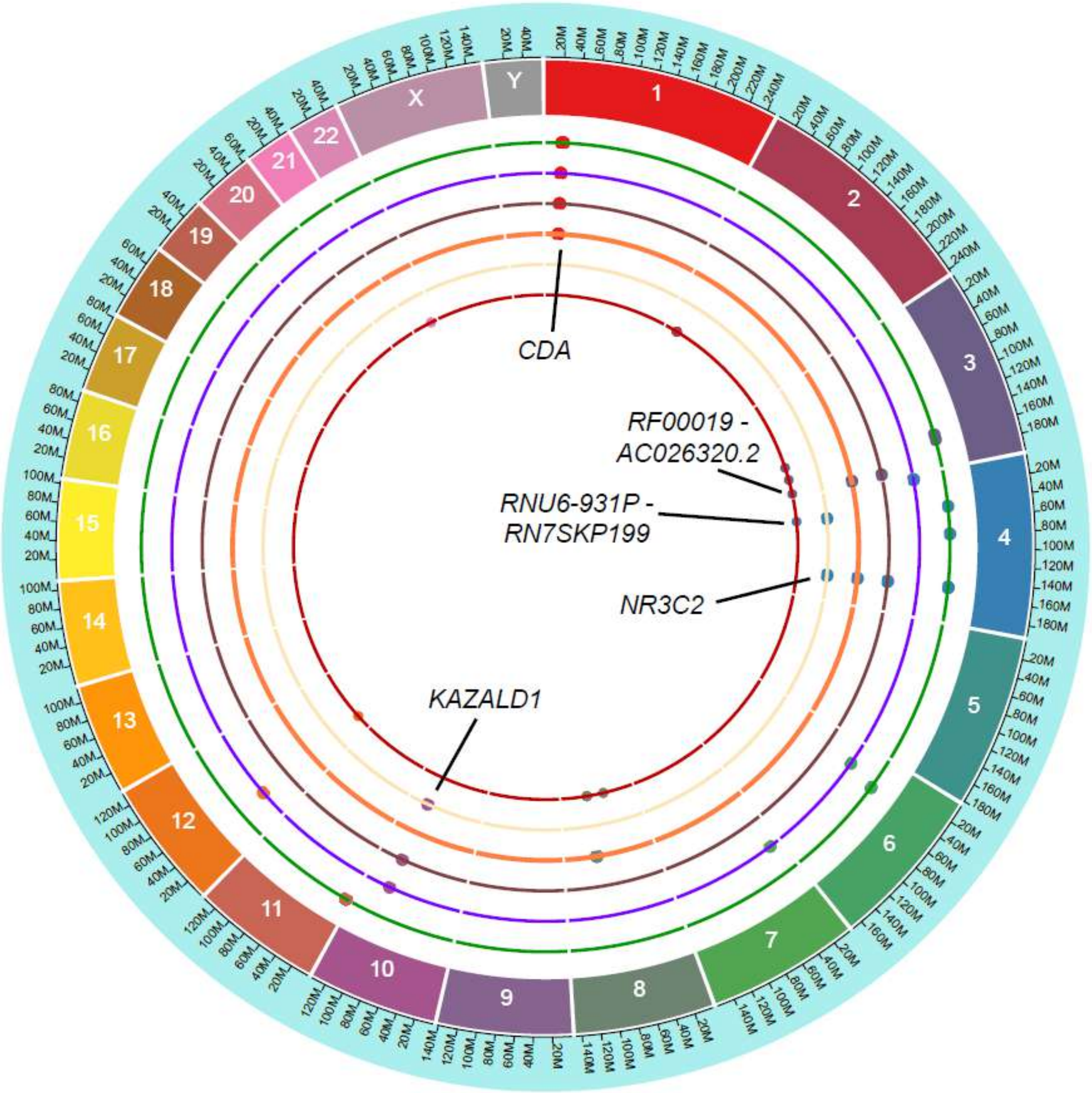

Supplementary Figure 15 - Map of significant GTEx eQTL overlapping non-coding regions with outlier CLR scores.

Chromosome regions (external ring) with non-coding outlier CLR scores (*P* <0.001, n=152) from the 7 HC-WGS populations (each with n=24 samples) are shown as columns in the middle segment (in between the inner and outer segments). Each GTEx tissue is colour-coded according to the tissue colour designations in GTEx. A coloured square indicates at least one significant eQTL in that tissue. The inner segment shows the number of tissues in which an eQTL was found for non-coding sites with at least one overlapping eQTL (ranges from 1 to 49).

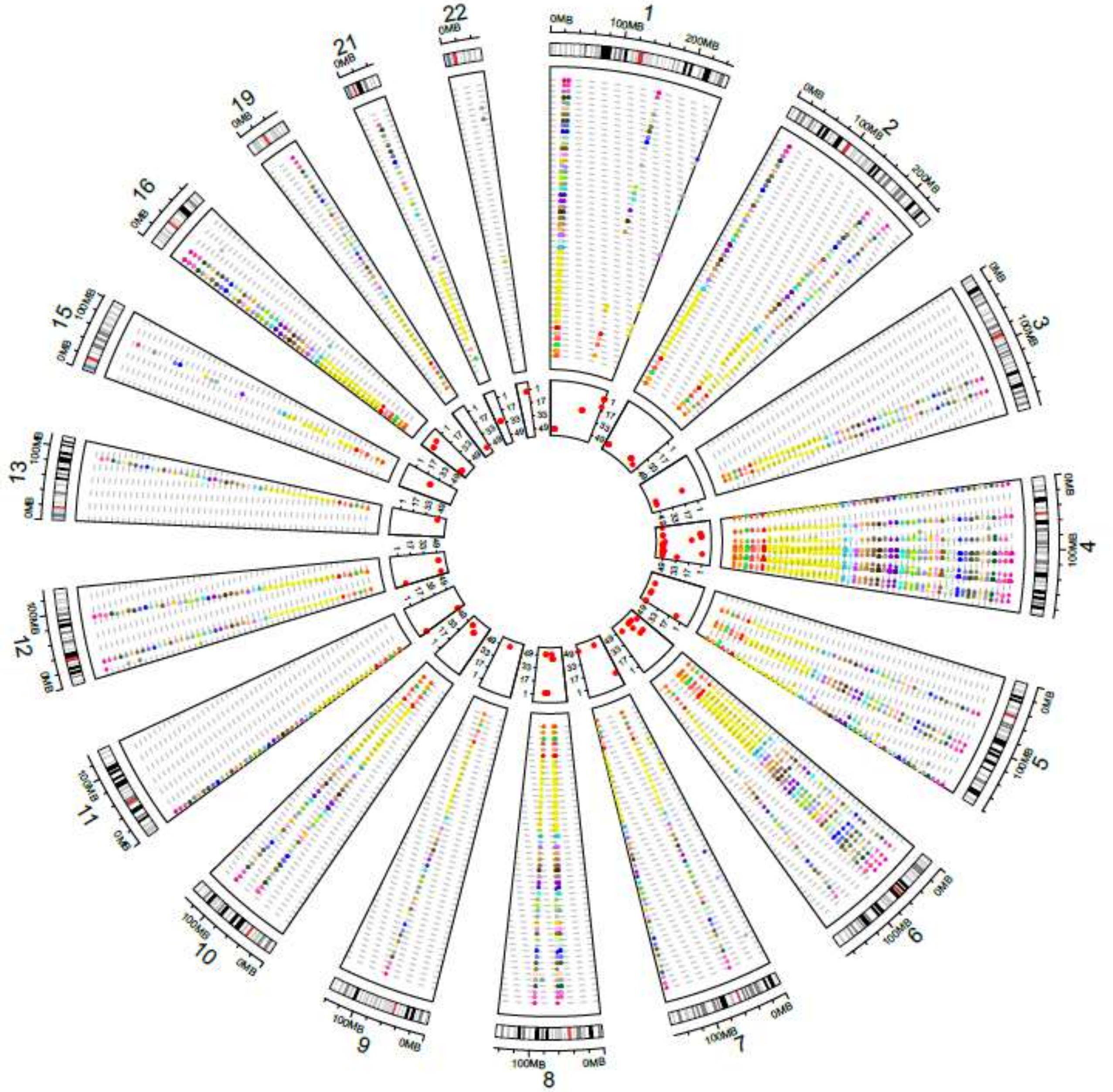

Supplementary Figure 16 - Significant GTEx eQTL overlapping non-coding regions with outlier CLR scores by tissue.

(A) Boxplots of random distributions of 10kb non-coding regions with CLR scores < 5th centile (n=14,088 independent regions) overlapping GTEx eQTLs for each GTEx tissue. The observed number of eQTLs in non-coding CLR outlier regions (n=152 regions) is shown as a red dot. **(B)** *t* scores per tissue from permuted data. Pink bars indicate the top 5 tissues with non-coding outlier CLR scores showing an excess number of eQTLs. Boxplots show median value (center box line) with whiskers representing the limits of the highest (4th, upper) and lowest (1st, lower) quartile of data; distribution outliers are shown as dots.

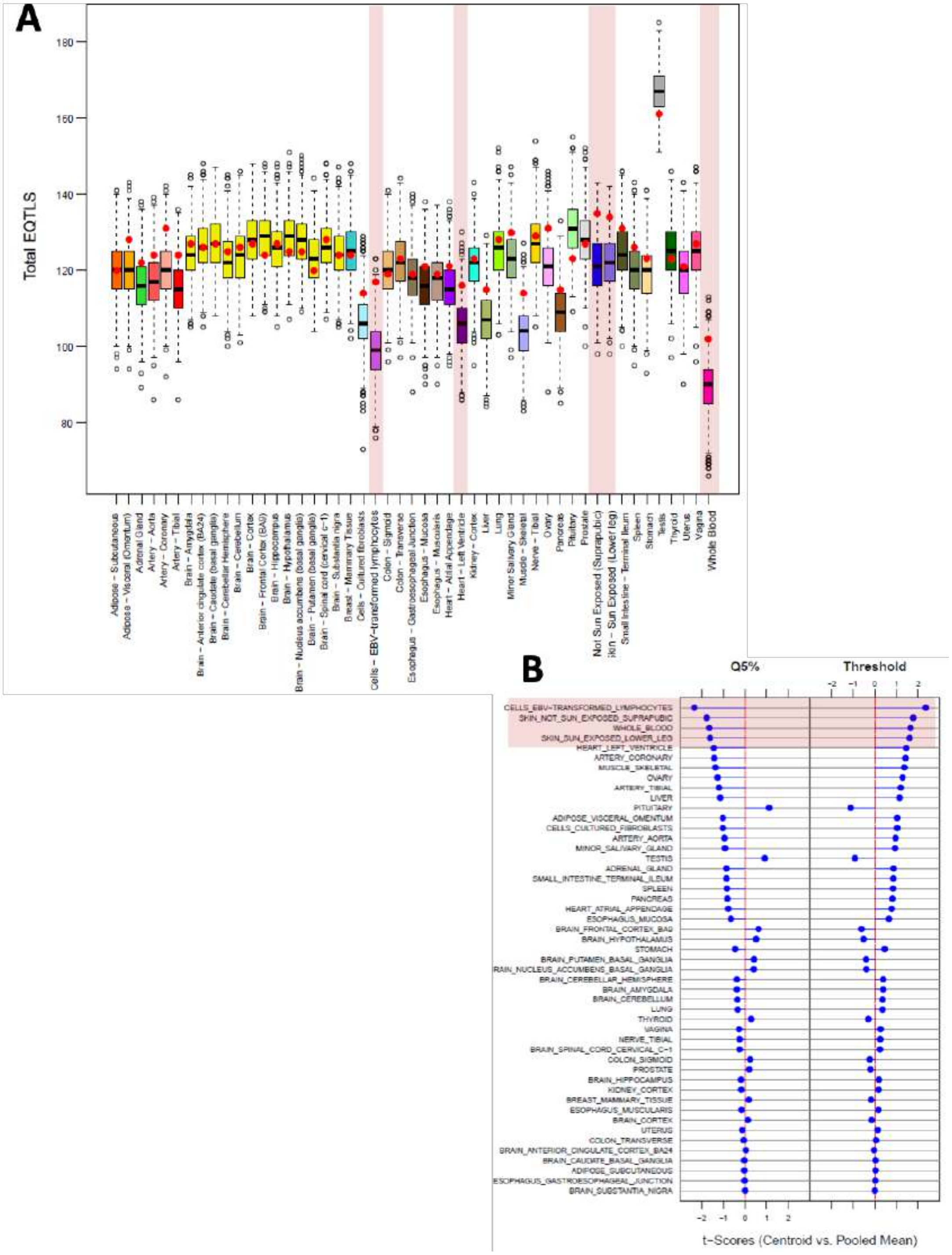

Supplementary Figure 17 - Significant GTEx eQTL overlapping non-coding regions with outlier CLR scores by tissue by population.

(**A**) For each population (numbers per **Supplementary Methods Table 1**), values were plotted as in **Supplementary Figure 16**; The overlap between populations for the implicated eQTLs is shown for whole blood (**B**), exposed skin (**C**), and left heart ventricle (**D**). Boxplots show median value (center box line) with whiskers representing the highest (4th, upper) and lowest (1st, lower) quartile of data; distribution outliers are shown as black dots.

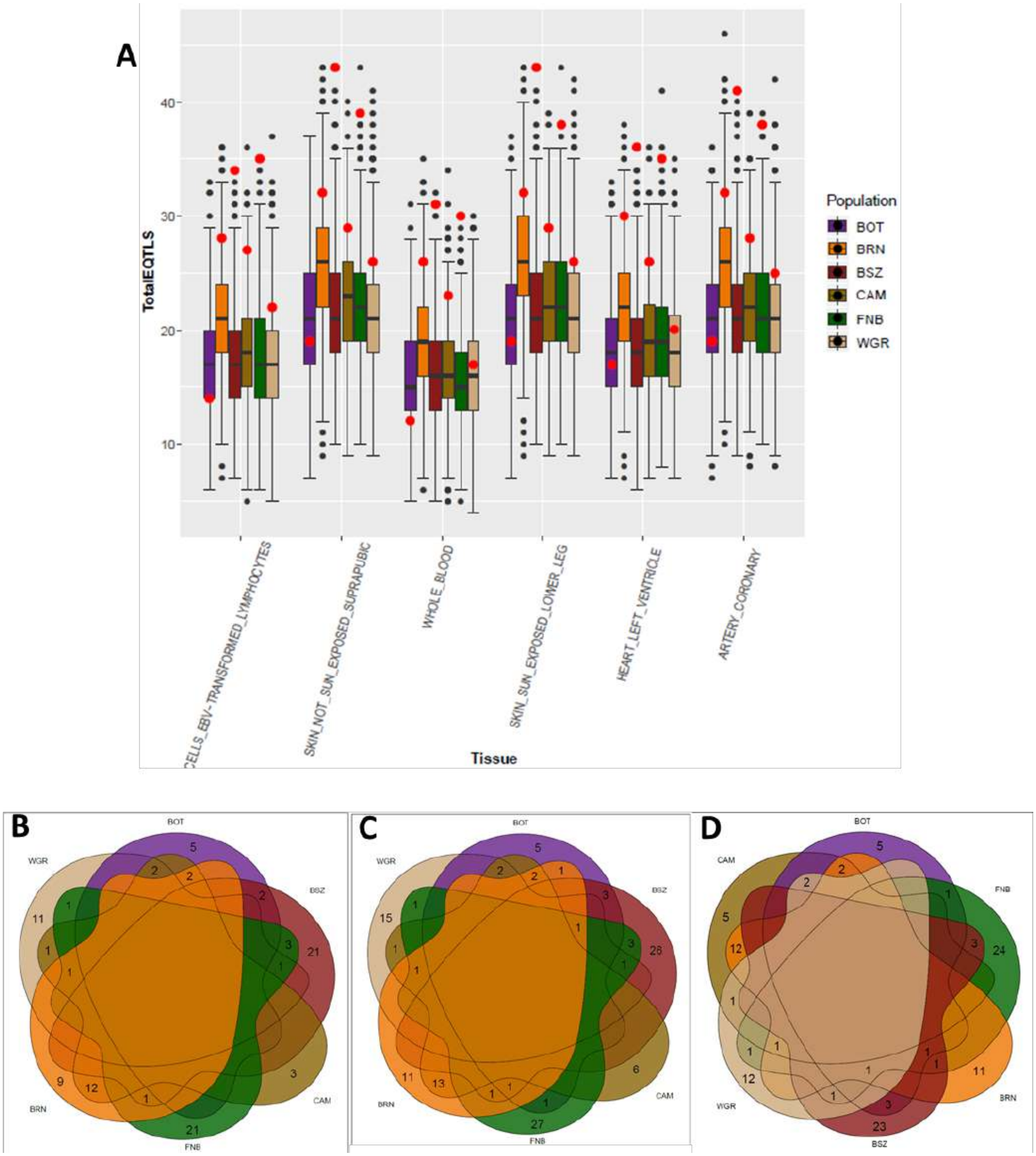

Supplementary Figure 18 – Site frequency spectra among high coverage WGS H3Africa populations by variant class.

Site frequency spectra for putative loss-of-function (LOF) variants, variants with a "high" (predicted damaging) and "low" (predicted benign) effect on the resulting protein are shown for each population group.

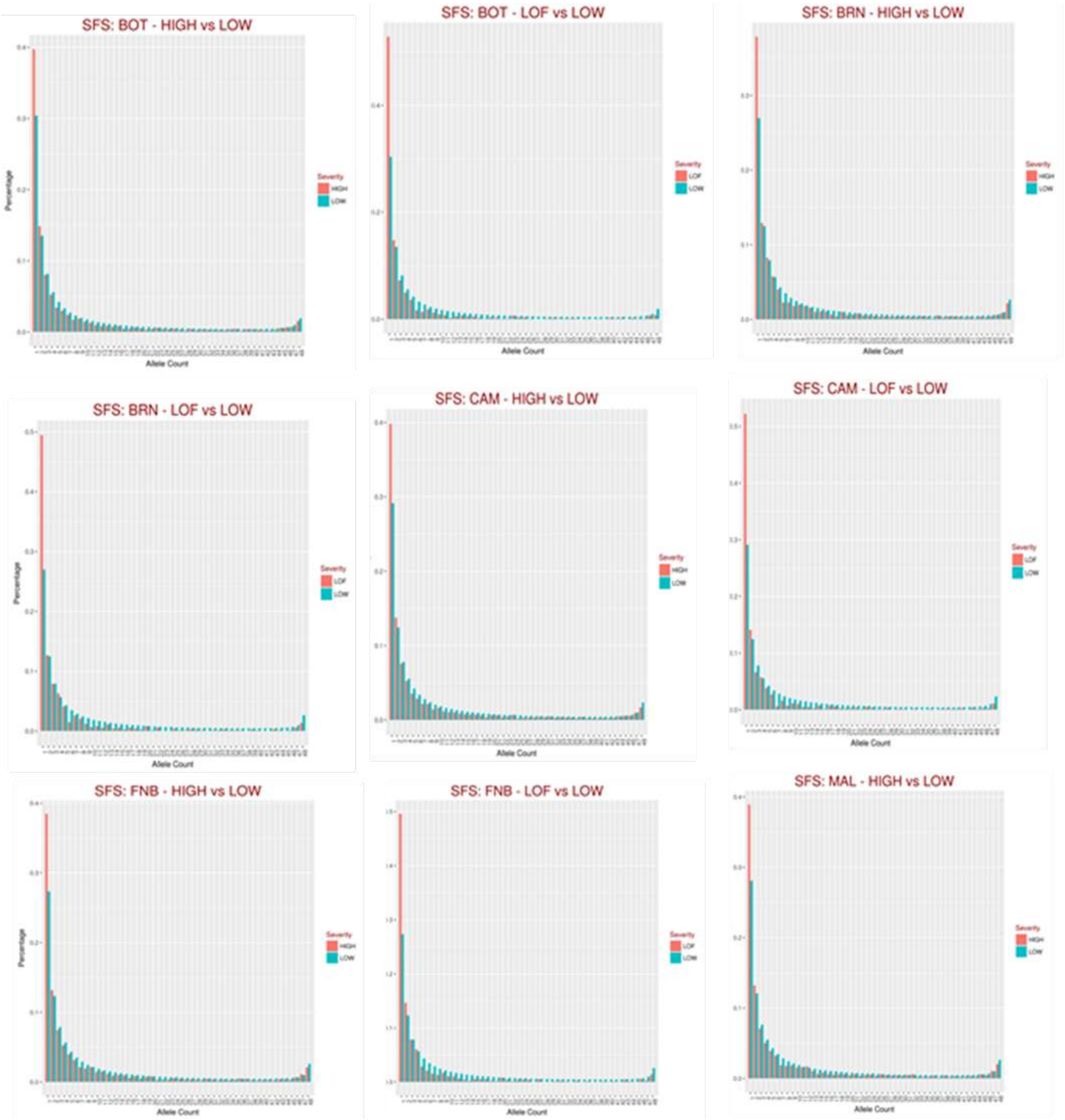

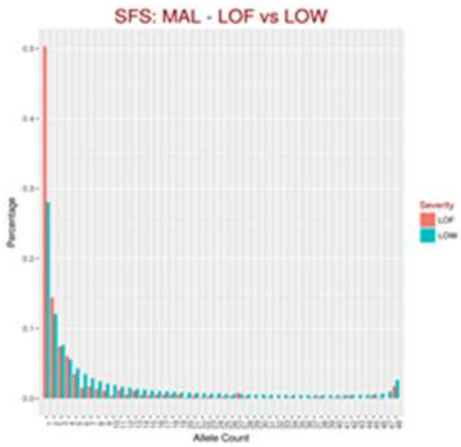
SFS: MAL - LOF vs LOW
Percentage
Allele Count
Severity
LOF
LOW

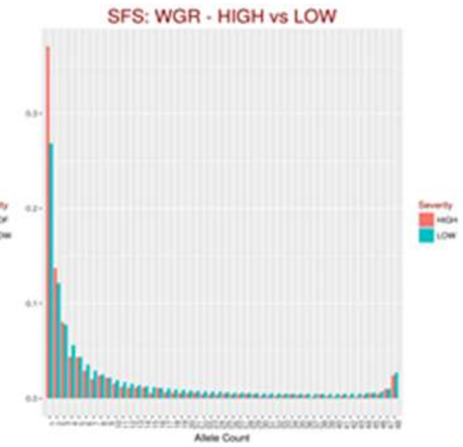
SFS: WGR - HIGH vs LOW
Allele Count
Severity
HIGH
LOW

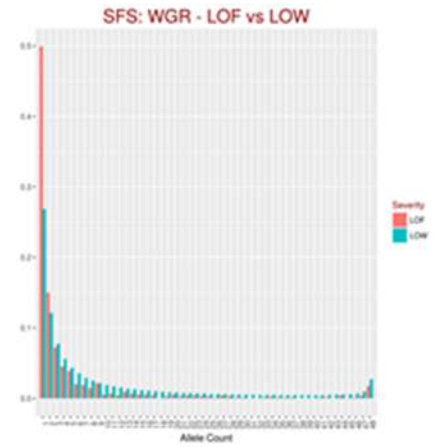
SFS: WGR - LOF vs LOW
Allele Count
Severity
LOF
LOW

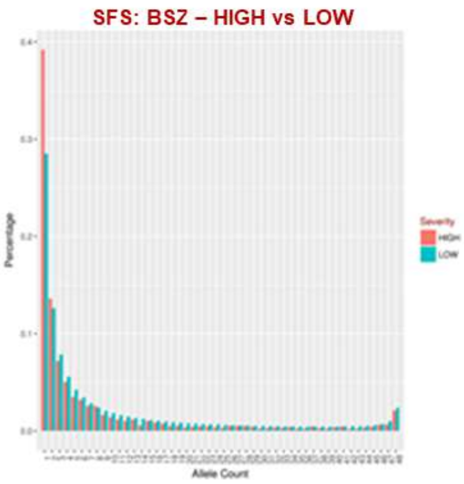
SFS: BSZ – HIGH vs LOW
Percentage
Allele Count
Severity
HIGH
LOW

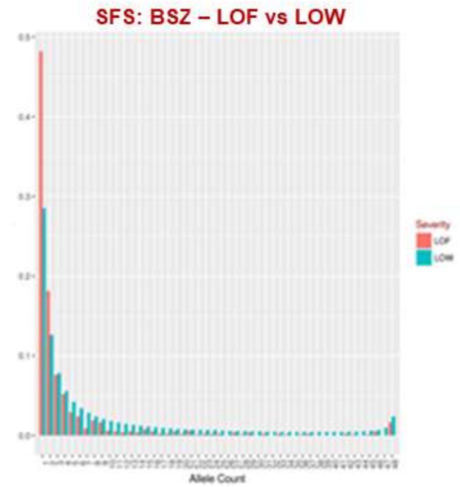
SFS: BSZ – LOF vs LOW
Allele Count
Severity
LOF
LOW

Supplementary Figure 19 - Heatmap of frequencies of common pLoF variants shared across H3Africa populations in *metabolic disease* genes

Also see **Extended Data Figure 3C**. pLoF variants with minor allele frequency >0.25 are shown**.**

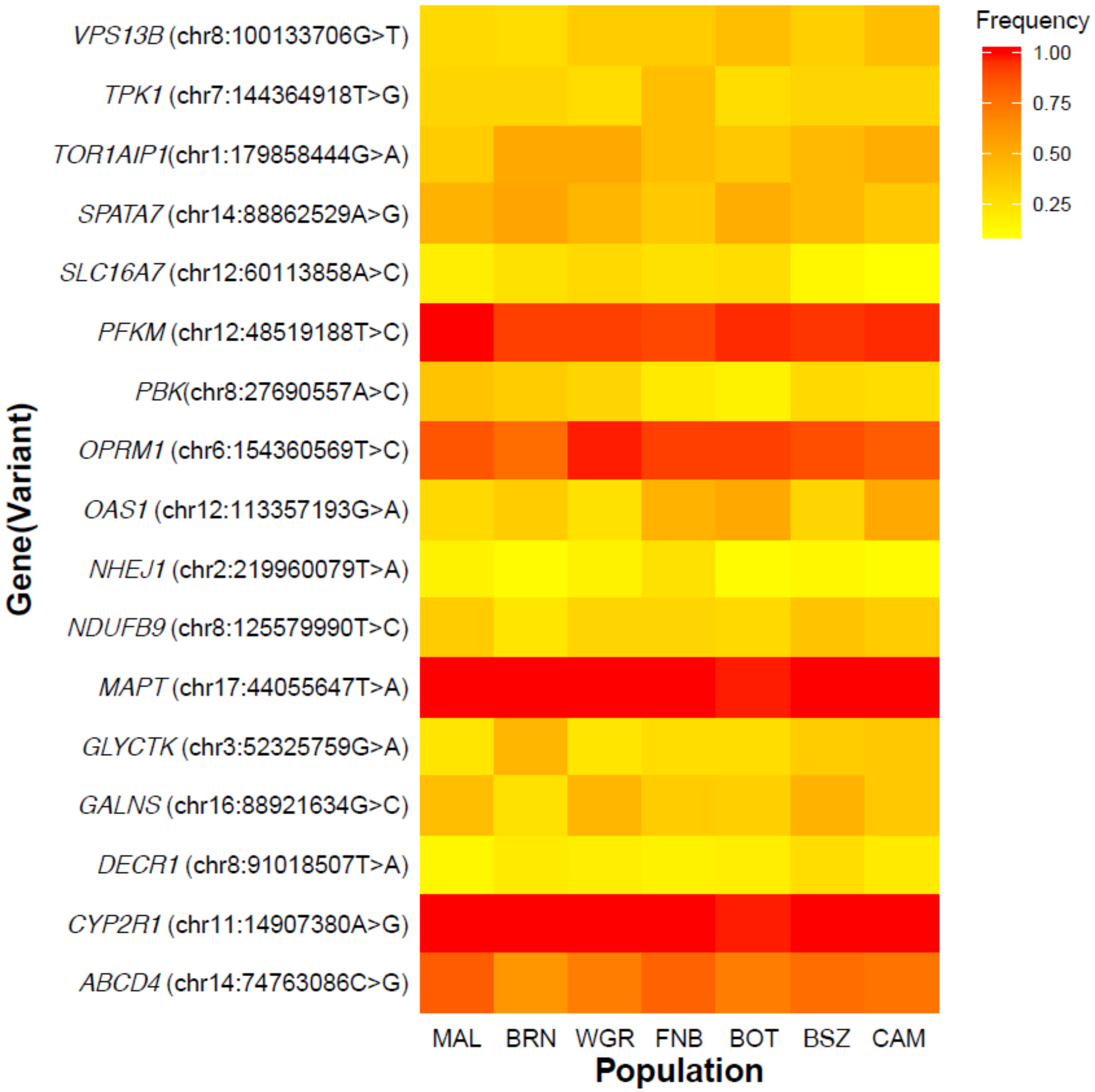

Supplementary Figure 20 – ClinVar Pathogenic (level 5) and likely-pathogenic (level 4) variants in 1000 Genomes African populations.

Distribution of allele frequencies for each variant (n=200) across all groups (left) and for each population (right) are shown. For comparison, the frequency of variants in *ZEB1* (c.(233A>C); *LDLRAP1* (c.(653C>T)), and *GATA4* (rs3735819) in Figures 4C and Extended Figure 4A are shown. ACB – African Caribbeans in Barbados, ASW – Americans of African Ancestry in SW USA; ESN – Esan in Nigeria; GWD – Gambian in Western Divisions in the Gambia; LWK- Luhya in Webuye, Kenya; MSL- Mende in Sierra Leone; YRI – Yoruba in Ibadan, Nigeria. Boxplots show median value (center box line) with whiskers representing the limits of the highest (4th, upper) and lowest (1st, lower) quartile of data; distribution outliers are shown as dots.

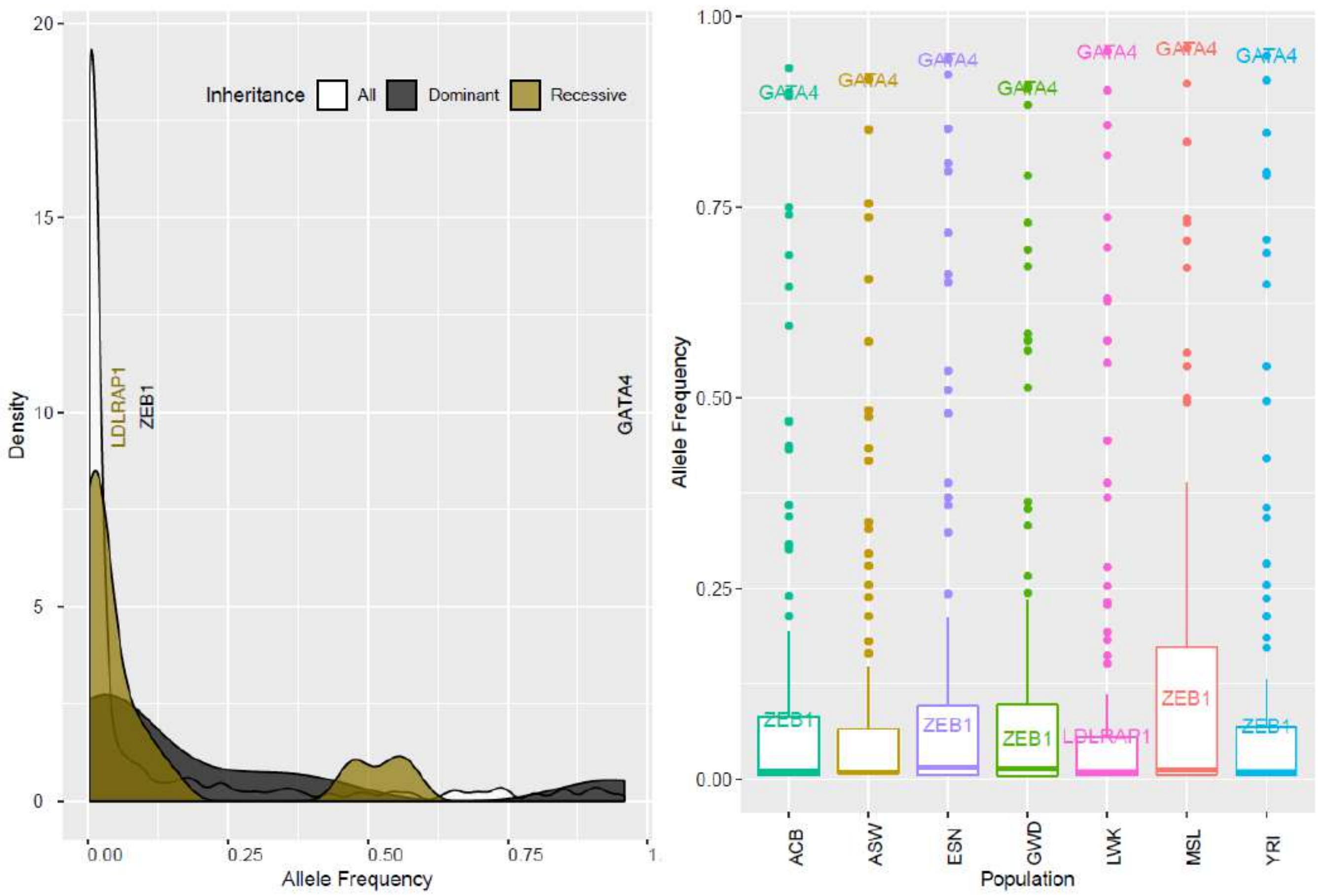

SUPPLEMENTARY METHODS FIGURES

Supplementary Methods Figure 1 - Overview of joint calling process resulting in merged VCF file. H3A-Baylor - high depth-of-coverage data from the H3Africa Consortium; TryopanoGEN - medium coverage data from the Trypanosomiasis Genomics Network of the H3Africa Consortium; SAHGP - Southern African Human Genome Programme.

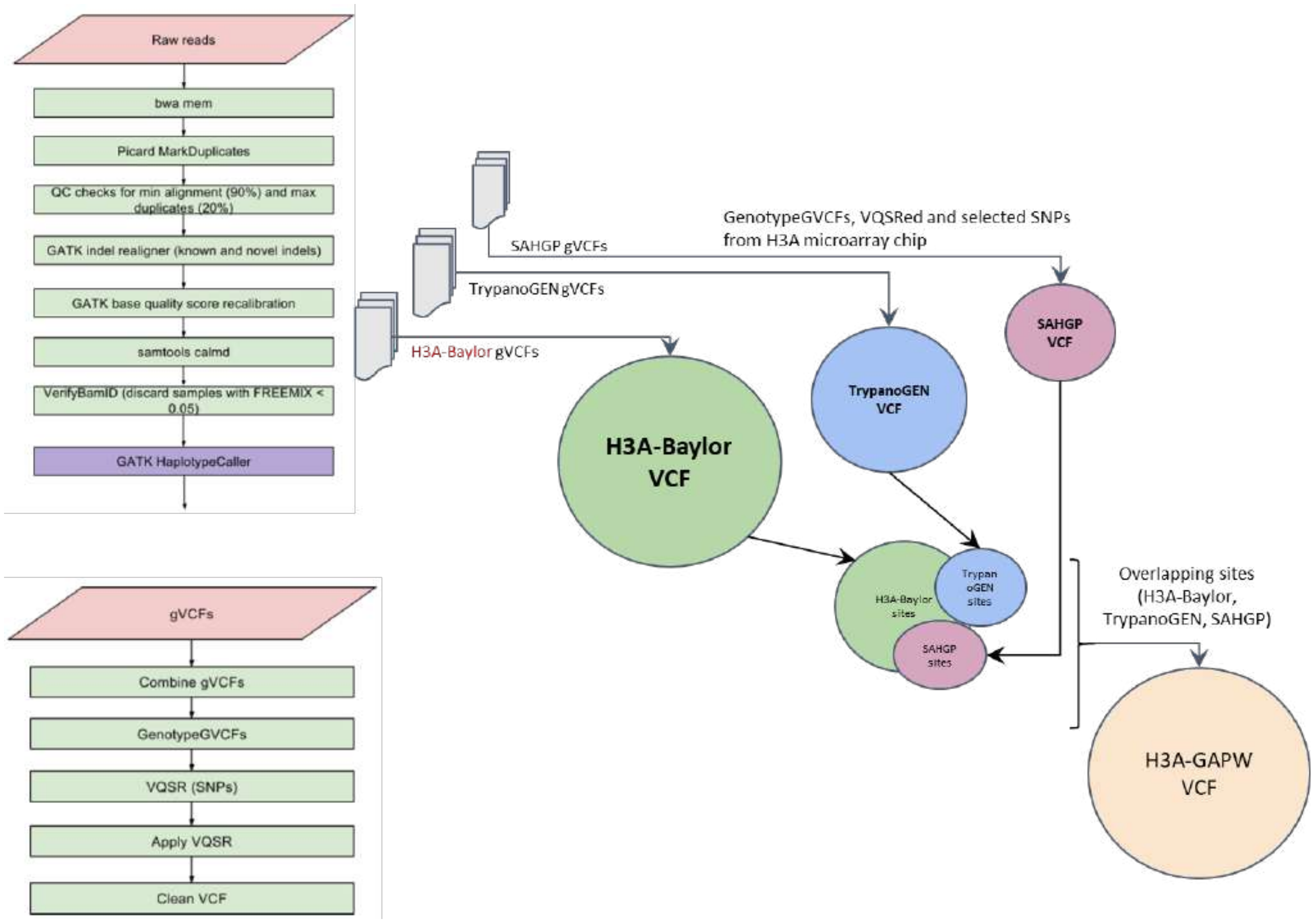

Supplementary Methods Figure 2 - Comparison of Influenza “direct” and “indirect” gene characteristics.

(**A**) Percent GC content and (**B**) gene lengths for genes with direct (n=181) and indirect (n=1,842) involvement in Influenza from GeneCards. For GC content comparisons, the two distributions had similar variance (two-sided F test = 0.95, $P = 0.68$) with mean values that were marginally different *a priori* (two-sided Welch 2-sample t-test, $P = 0.034$), but not significantly so after random sampling to account for the differences in the number of genes (100 random genes sampled from each gene set 1000 times and means compared using one-sided Welch t-test, mean $P = 0.30$). For gene length comparison the two distributions had similar means (two-sided Welch t test, t = 0.66, df = 204.19, $P = 0.51$), with unequal variances (two-sided F test=0.79, P=0.047), indicating no significant difference *a priori* or after similar random sampling (mean $P = 0.23$). Boxplots show median value (center box line) with whiskers representing the limits of the highest (4th, upper) and lowest (1st, lower) quartile of data; distribution outliers are shown as dots.

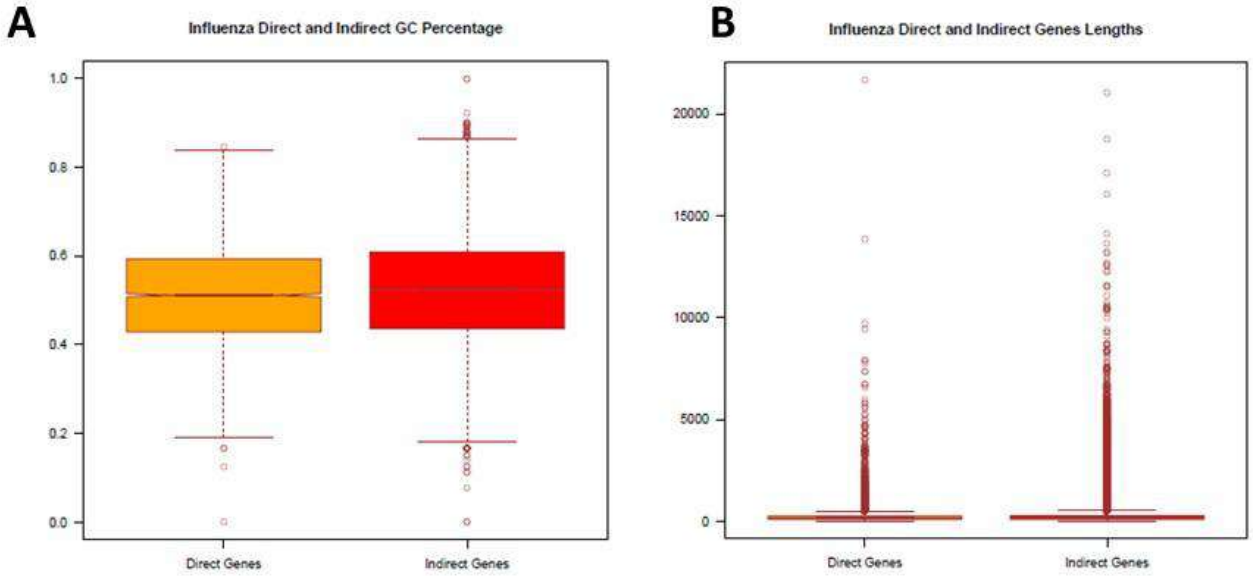

Supplementary Methods Figure 3 - Benchmarking of pLOF burden ratio.

Benchmarks were performed using number of genes with pLOFs among genes with direct (n=181) or indirect (n=1,842) involvement in Influenza from Genecards (**Supplementary Table 18**) for each population (n=24 each population). **(A)** Ratio of pLoF-containing indirect genes for given population to pLoF-containing indirect genes in all populations; **(B)** Number of direct genes with pLoFs for given population only; **(C)** Ratio of pLoF-containing direct genes in given population to total number of genes with pLoFs; **(D)** Ratio of pLoF-containing direct genes to pLoFs-containing indirect genes in given population; (**E**) Ratio of pLoF-containing direct genes in given population to number of direct genes in any other population; (**F**) Ratio of any pLoF-containing gene in given population to number of pLoF-containing genes in any other population**.** Red lines are the line of correlation using a linear model and the shaded areas the associated 95% confidence intervals.

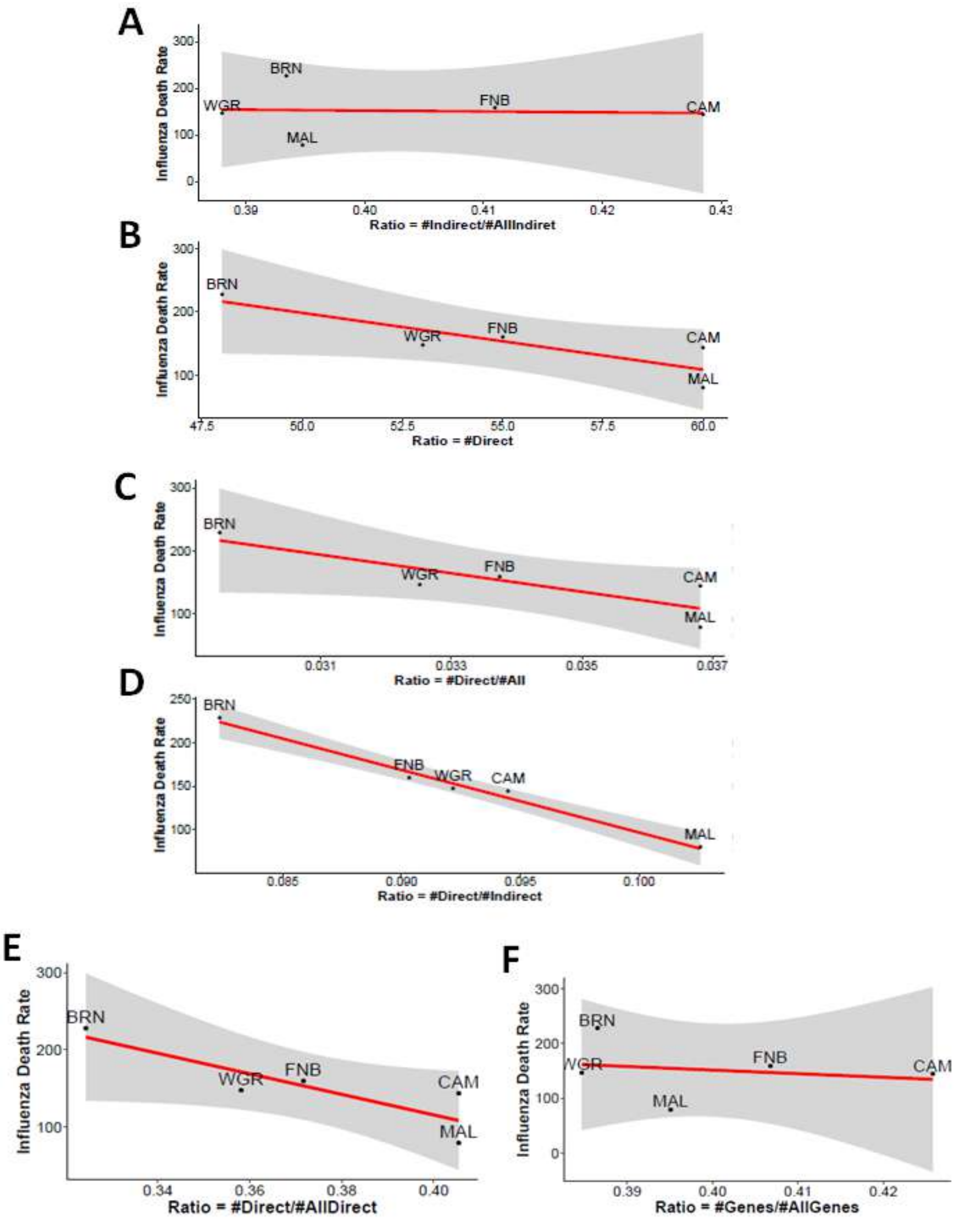

REFERENCES

1 Guldemann, T. in *Beyond 'Khoisan'* (eds Tom Güldemann & A. M. Fehn) (John Benjamins Publishing Company, 2014).

2 Chennells, R. & A, S. in *Ethics Dumping. SpringerBriefs in Research and Innovation Governance.* (eds Schroeder D. *et al.*) 15-22 (Springer, 2017).

3 Hollfelder, N. *et al.* Northeast African genomic variation shaped by the continuity of indigenous groups and Eurasian migrations. *PLoS Genetics* **13**, e1006976-e1006976 (2017).

4 Bryc, K. *et al.* Genome-wide patterns of population structure and admixture in West Africans and African Americans. *Proceedings of the National Academy of Sciences* **107**, 786-791 (2010).

5 Pagani, L. *et al.* Genomic analyses inform on migration events during the peopling of Eurasia. *Nature* **538**, 238-242 (2016).

6 Triska, P. *et al.* Extensive Admixture and Selective Pressure Across the Sahel Belt. *Genome Biology and Evolution* **7**, 3484-3495 (2015).

7 1000 Genomes Project Consortium *et al.* A global reference for human genetic variation. *Nature* **526**, 68-74 (2015).

8 Haber, M. *et al.* Chad Genetic Diversity Reveals an African History Marked by Multiple Holocene Eurasian Migrations. *American Journal of Human Genetics* **99**, 1316-1324 (2016).

9 Tishkoff, S. A. *et al.* The genetic structure and history of Africans and African Americans. *Science* **324**, 1035-1044 (2009).

10 Maples, B. K., Gravel, S., Kenny, E. E. & Bustamante, C. D. RFMix: a discriminative modeling approach for rapid and robust local-ancestry inference. *American Journal of Human Genetics* **93**, 278-288 (2013).

11 Gurdasani, D. *et al.* The African Genome Variation Project shapes medical genetics in Africa. *Nature* **517**, 327-332 (2015).

12 Schlebusch, C. M. *et al.* Genomic Variation in Seven Khoe-San Groups Reveals Adaptation and Complex African History. *Science* **338**, 374-379 (2012).

13 Auton, A. *et al.* Global distribution of genomic diversity underscores rich complex history of continental human populations. *Genome Research* **19**, 795-803 (2009).

14 Patterson, N. *et al.* Ancient admixture in human history. *Genetics* **192**, 1065-1093 (2012).

15 Patin, E. *et al.* Dispersals and genetic adaptation of Bantu-speaking populations in Africa and North America. *Science* **356**, 543-546 (2017).

16 Plateau Indigenous Development Association Network. *The history, ownership, establishment of Jos and misconceptions about the recurrent Jos conflicts*. (DAN-SiL Press, 2010).

17 Breunig, P. *Nok: African sculpture in archaeological context*. (Africa Magna Verlag, 2014).

18 Fwatshak, S. U. Reconstructing the origins of the peoples of plateau state: questioning the "we are all settlers" theory. *Journal of the Historical Society of Nigeria* **16**, 122-140 (2005).

19 Shoup, J. A. *Ethnic Groups of Africa and the Middle East: An Encyclopedia (Ethnic Groups of the World)*. (ABC-CLIO, 2011).

20 Weissensteiner, H. *et al.* HaploGrep 2: mitochondrial haplogroup classification in the era of high-throughput sequencing. *Nucleic Acids Research* **44**, W58-W63 (2016).

21 Van Geystelen, A., Decorte, R. & Larmuseau, M. H. D. AMY-tree: An algorithm to use whole genome SNP calling for Y chromosomal phylogenetic applications. *BMC Genomics* **14**, 101-101 (2013).

22 Chan, E. K. *et al.* Revised timeline and distribution of the earliest diverged human maternal lineages in southern Africa. *PLoS ONE* **10**, e0121223 (2015).

23 Pereira, L. *et al.* Linking the sub-Saharan and West Eurasian gene pools: maternal and paternal heritage of the Tuareg nomads from the African Sahel. *European Journal of Human Genetics* **18**, 915-923 (2010).

24 Hanchard, N. *et al.* Classical sickle beta-globin haplotypes exhibit a high degree of long-range haplotype similarity in African and Afro-Caribbean populations. *BMC Genetics* **8**, 52-52 (2007).
25 MacArthur, J. *et al.* The new NHGRI-EBI Catalog of published genome-wide association studies (GWAS Catalog). *Nucleic Acids Research* **45**, D896-D901 (2017).
26 Patel, K. V. *et al.* Haemoglobin concentration and the risk of death in older adults: differences by race/ethnicity in the NHANES III follow-up. *British Journal of Haematology* **145**, 514-523 (2009).
27 Catherino, W. H., Eltoukhi, H. M. & Al-Hendy, A. Racial and ethnic differences in the pathogenesis and clinical manifestations of uterine leiomyoma. *Seminars in Reproductive Medicine* **31**, 370-379 (2013).
28 Laster, M., Shen, J. I. & Norris, K. C. Kidney Disease Among African Americans: A Population Perspective. *American Journal of Kidney Disease* **72**, S3-S7 (2018).
29 GTEx Consortium. The Genotype-Tissue Expression (GTEx) project. *Nature Genetics* **45**, 580-585 (2013).
30 Yi, X. *et al.* Sequencing of 50 human exomes reveals adaptation to high altitude. *Science* **329**, 75-78 (2010).
31 Owers, K. A. *et al.* Adaptation to infectious disease exposure in indigenous Southern African populations. *Proceedings of the Royal Society B: Biological Sciences* **284** (2017).
32 Crawford, N. G. *et al.* Loci associated with skin pigmentation identified in African populations. *Science* **358**, eaan8433-eaan8433 (2017).
33 Janciauskiene, S. M. *et al.* The discovery of alpha1-antitrypsin and its role in health and disease. *Respiratory Medicine* **105**, 1129-1139 (2011).
34 Brenaut, P. *et al.* Contribution of mammary epithelial cells to the immune response during early stages of a bacterial infection to Staphylococcus aureus. *Veterinary Research* **45**, 16 (2014).
35 Forssmann, W. G. *et al.* Short-term monotherapy in HIV-infected patients with a virus entry inhibitor against the gp41 fusion peptide. *Science Translational Medicine* **2**, 63re63 (2010).
36 Retshabile, G. *et al.* Whole-Exome Sequencing Reveals Uncaptured Variation and Distinct Ancestry in the Southern African Population of Botswana. *American Journal of Human Genetics* **102**, 731-743 (2018).
37 Mosepele, M. *et al.* Pre-clinical carotid atherosclerosis and sCD163 among virally suppressed HIV patients in Botswana compared with uninfected controls. *PLoS ONE* **12**, 1-14 (2017).
38 Matthaei, M. *et al.* Changing Indications in Penetrating Keratoplasty: A Systematic Review of 34 Years of Global Reporting. *Transplantation* **101**, 1387-1399 (2017).
39 Choudhury, A. *et al.* Whole-genome sequencing for an enhanced understanding of genetic variation among South Africans. *Nature Communications* **8**, 1-12 (2017).
40 R: A language and environment for statistical computing. (R Foundation for Statistical Computing, Vienna, Austria., 2017).
41 Pagani, L. *et al.* Ethiopian genetic diversity reveals linguistic stratification and complex influences on the Ethiopian gene pool. *American Journal of Human Genetics* **91**, 83-96 (2012).
