# Supplementary material for: High-depth African genomes inform human migration and health
Source: Nature. 2020 Oct 28;586(7831):741–8. doi: 10.1038/s41586-020-2859-7 (PMC7759466; doi:10.1038/s41586-020-2859-7)
Supplement: Supplementary file 2 — Reporting Summary [file 41586_2020_2859_MOESM2_ESM.pdf]

nature research

Corresponding author(s): Adebowale Adeyemo, Zane Lombard, Neil A.Hanchard

Last updated by author(s): 2020 - 04 - 03

Reporting Summary

Nature Research wishes to improve the reproducibility of the work that we publish. This form provides structure for consistency and transparency in reporting. For further information on Nature Research policies, see Authors & Referees and the Editorial Policy Checklist.

Statistics

For all statistical analyses, confirm that the following items are present in the figure legend, table legend, main text, or Methods section.

n/a	Confirmed	
☐	☒	The exact sample size (n) for each experimental group/condition, given as a discrete number and unit of measurement
☒	☐	A statement on whether measurements were taken from distinct samples or whether the same sample was measured repeatedly
☐	☒	The statistical test(s) used AND whether they are one- or two-sided<br>*Only common tests should be described solely by name; describe more complex techniques in the Methods section.*
☒	☐	A description of all covariates tested
☐	☒	A description of any assumptions or corrections, such as tests of normality and adjustment for multiple comparisons
☐	☒	A full description of the statistical parameters including central tendency (e.g. means) or other basic estimates (e.g. regression coefficient) AND variation (e.g. standard deviation) or associated estimates of uncertainty (e.g. confidence intervals)
☐	☒	For null hypothesis testing, the test statistic (e.g. F, t, r) with confidence intervals, effect sizes, degrees of freedom and P value noted<br>*Give P values as exact values whenever suitable.*
☒	☐	For Bayesian analysis, information on the choice of priors and Markov chain Monte Carlo settings
☒	☐	For hierarchical and complex designs, identification of the appropriate level for tests and full reporting of outcomes
☒	☐	Estimates of effect sizes (e.g. Cohen's d, Pearson's r), indicating how they were calculated

Our web collection on statistics for biologists contains articles on many of the points above.

Software and code

Policy information about availability of computer code

Data collection

No software was used for data collection.

Data analysis

(More detailed software information can be found in Supplementary Table 23)
Bedtools 2.25.0
ClinVar 01-09-2017
OMIM 01-10-2017
HapMap Phase III
BWA Software Package 0.7.10,0.7.17
PICARD 1.119,2.18.11
Samtools 0.1.19,1.9
GATK Tools 3.3, 3.5, 4.0.8.0
dbSNP Build 150
VerifyBamID 1.1.1, 1.1.3
Eagle 2.0
SHAPEIT 2.17
PLINK 1.90
ADMIXTURE 1.3.0
GENESIS 0.2.6
CLUMPP 1.1.2
ADMIXTOOLS 1.0
TREEMIX 1.1.3
VCFTools 0.1.15
Selscan 1.1.0b
Ensembl 01-10-2017
GeneCards 01-10-2017

gnomAD 2.0.2
exomeAD 2.0.3
BioMart 2.34.2
eDGAR 10-11-2016
HGVD 2.3
GWAS Catalogue v.2019-10-14
VEP 90
GTEx 7
MarViN r1 https://github.com/Illumina/MarViN
EIGENSOFT v.7.2.1
MALDER v.1.0
Beagle 4.1.
RFMix_v2
SNPEff 4.3-3
ExAC r2.01
Haplogrep2 2.1.1.
AMY-tree 2.0
ClueGO v.2.5.1
ALOFT 1.0
DisGenet 6.0
eDGAR database release 10/11/2016
CTDbase update 2019
SWEED 3.3.1
dbPHSP https://jjwanglab.org/dbphsp

For manuscripts utilizing custom algorithms or software that are central to the research but not yet described in published literature, software must be made available to editors/reviewers. We strongly encourage code deposition in a community repository (e.g. GitHub). See the Nature Research guidelines for submitting code & software for further information.

Data

Policy information about availability of data

All manuscripts must include a data availability statement. This statement should provide the following information, where applicable:

- Accession codes, unique identifiers, or web links for publicly available datasets
- A list of figures that have associated raw data
- A description of any restrictions on data availability

WGS data from the H3Africa projects used in this study have been deposited in the European Genome-phenome Archive (EGA) with accession numbers:

H3AfricaChipDesign: Study EGAS00001002976
TrypanoGEN1: Data Set EGAD00001004393
TrypanoGEN2: Data Set EGAD00001004220
MALSIC: Data Set EGAD00001004557
AWI-GEN: Data Set EGAD00001004448
CAfGEN: Data Set EGAD00001004533
ELSI: Data Set EGAD00001004316
NEEDI: Data Set EGAD00001004334
ACCME: Data Set EGAD00001004505

DNA samples are archived in H3Africa biorepositories as part of the H3Africa Consortium agreement. The Data and biospecimens are available on request through the Data and Biospecimen Access Committee of the H3Africa consortium. Novel SNVs identified and reported here will be deposited into the database of Single Nucleotide Polymorphisms (dbSNP).

Field-specific reporting

Please select the one below that is the best fit for your research. If you are not sure, read the appropriate sections before making your selection.

☒ Life sciences ☐ Behavioural & social sciences ☐ Ecological, evolutionary & environmental sciences

For a reference copy of the document with all sections, see nature.com/documents/nr-reporting-summary-flat.pdf

Life sciences study design

All studies must disclose on these points even when the disclosure is negative.

Sample size

No statistical method was used to determine sample size. Sample size was maximised to include as wide a diversity of African populations and ethnolinguistic groups as possible. For specific analysis methods, subsets of the data (e.g. high depth of coverage data) were used to ensure robust conclusions.

Data exclusions

Exclusion criteria were pre-established on the basis of relatedness and sequencing quality. Samples were excluded based on sequencing-quality control metrics generated prior to any downstream analysis; this included poor mapping of reads to the reference genome, missingness thresholds, and familial relatedness. Single nucleotide variants were excluded based on excessive missingness, deviations from

Hard-Weinberg equilibrium, call ambiguity and low minor allele frequencies. In addition, where a genetically homogeneous set of individuals was required for an analysis, possible outliers were identified and removed.

Replication	Results were not externally replicated.
Randomization	The experiments were not randomized.
Blinding	Investigators were not blinded to the allocation during analyses, since this it not an association study.

Reporting for specific materials, systems and methods

We require information from authors about some types of materials, experimental systems and methods used in many studies. Here, indicate whether each material, system or method listed is relevant to your study. If you are not sure if a list item applies to your research, read the appropriate section before selecting a response.

Materials & experimental systems

n/a	Involved in the study	
☒	☐	Antibodies
☒	☐	Eukaryotic cell lines
☒	☐	Palaeontology
☒	☐	Animals and other organisms
☐	☒	Human research participants
☒	☐	Clinical data

Methods

n/a	Involved in the study	
☒	☐	ChIP-seq
☒	☐	Flow cytometry
☒	☐	MRI-based neuroimaging

Human research participants

Policy information about studies involving human research participants

Population characteristics

There are no covariate-relevant population characteristics. Individuals were chosen to represent a broad geographical and ethnolinguistic diversity and were sampled from three ongoing genomics consortium projects on the continent - the H3Africa Consortium, the Southern African Human Genome Programme (SAHGP), and the Trypanosomiasis Genomics Network of the H3Africa Consortium (TryopanoGEN).

Recruitment

The participants in the study were previously recruited to individual cohorts as part of ongoing genomics projects on the continent:
(i) Samples from 519 individuals from 8 projects in the H3Africa consortium were submitted for consideration. Individuals were predominantly recruited as control individuals from the respective studies, with the exception of those from case-only studies in Cameroon (Sickle cell), Botswana (HIV), Mali (families with neurological disease), and Benin (sickle cell). Individuals with high relatedness (PIHAT> 0.18) were removed from the analysis. Individuals comprising the Berom from Nigeria were recruited as controls for a cervical cancer study, and thus are all female.
(ii) A total of 300 (control) individuals were recruited from five countries (Uganda, Zambia, Democratic Republic of the Congo, Cameroon and Ivory Coast) participating in the TrypanoGEN project to study the host and parasite genetics and genomics of trypanosomiasis infection across Africa.
(iii) 16 (unaffected) individuals were recruited by the Southern African Human Genome Programme. These individuals met inclusion criteria: male, over the age of 18 years, four grandparents who speak the same language as the participant, not known to be related to the other participants in the study, and willing to provide broad informed consent (including consent to share data and DNA for future studies approved by the HREC (Medical)). Two main Bantu-speaking ethnolinguistic groups were included: The Sotho (Sotho-Tswana speakers; n=8) were recruited from in and around the town of Ventersburg in the Free State Province and the Xhosa speakers (Nguni language; n=7) were recruited from the Eastern Cape Province. One individual was a Zulu speaker (Nguni language) from Johannesburg.

Ethics oversight

Ethics approval (country and institutional) was obtained by each PI of the individual studies. We attach further details of each study's approval, which will be included in the online methods and supplementary materials section of the paper (detailed information was not included in initial submission).

Note that full information on the approval of the study protocol must also be provided in the manuscript.
